# Endothelial glycocalyx thickness in cats with naturally occurring trauma or non-traumatic illness: an exploratory study

**DOI:** 10.3389/fvets.2026.1751034

**Published:** 2026-02-23

**Authors:** Ivayla D. Yozova, Neroli Thomson, Mitsuhiro Irie, Rebecca Owen, John S. Munday

**Affiliations:** 1 School of Veterinary Science, Massey University, Palmerston North, New Zealand; 2 Shikoku Veterinary Medical Center, Miki, Japan

**Keywords:** APPLE_fast_, ATT, capillaries, feline, GlycoCheck^TM^, length of hospitalisation, microcirculation, perfused boundary region

## Abstract

**Introduction:**

Endothelial glycocalyx damage contributes to morbidity and mortality in critical illness. In this exploratory study in cats with trauma and non-traumatic illness, the endothelial glycocalyx thickness was estimated using sidestream dark field videomicroscopy and the Glycocheck™ software, with the primary aims of assessing feasibility, describing of Glycocheck™ microcirculatory parameters and exploring potential effects of illness severity and IV fluid administration.

**Methods:**

This was a prospective, single center, observational study. Recorded variables included age, weight, diagnosis, length of hospitalization (LOH), packed cell volume (PCV) and IV fluid administration. Each patient was assigned a fast Acute Patient Physiologic and Laboratory Evaluation score (APPLE_fast_) and for cats with trauma - Animal Trauma Triage score (ATT). Within 24 h from admission, cats were anesthetized, and images from the sublingual mucosal vessels obtained for Glycocheck™ analysis. The perfused boundary region (PBR), an inverse estimate for endothelial glycocalyx thickness, was calculated for vessels with diameter of 5–25, 5–9, 10–19 and 20–25 μm, respectively. Normality was assessed using Shapiro–Wilk test and histograms. The effects of APPLE_fast_, LOH, ATT, PCV, IV fluid administration, and illness group on PBR were analysed using generalised linear models. Distribution of vascular segment counts was assessed using Friedman’s test and Wilcoxon rank-signed test.

**Results:**

Nineteen cats were included, 11 with trauma. Success rate for measurements was 95%. Survival to discharge was 95%. Mean ± standard deviation for PBR 5–25 was 2.60 ± 0.22 μm, PBR 5–9: 1.38 ± 0.15 μm, PBR 10–19: 3.02 ± 0.22 μm and PBR 20–25: 3.03 ± 0.38 μm, within previously established tolerance intervals. There were no statistically significant effects of group, LOH, ATT, APPLE_fast_, PCV and IV fluids on any PBR measurements. Vascular segment counts across the 5–25 μm range were not equally distributed (*p* < 0.001).

**Conclusion:**

This exploratory study demonstrates the feasibility of assessing endothelial glycocalyx thickness in hospitalized sick cats. While no effects of clinical variables on PBR were identified, the study highlights important methodological considerations and provides valuable insights to guide future, larger-scale investigations.

## Introduction

Microcirculatory abnormalities are a recognised contributor to increased morbidity and mortality in critically ill people (1–3). More recently, damage to the endothelial glycocalyx (EG) has been proposed as a key factor to these disturbances (4–7) and found to correlate with illness severity in people with trauma (8, 9), sepsis (3, 10) and kidney disease (11, 12).

Endothelial glycocalyx degradation contributes to microvascular dysfunction and the progression to multi-organ failure. The underlying mechanisms are multifactorial. Systemic inflammatory states (such as sepsis, severe trauma and ischemia reperfusion injury) lead to cytokine release (13), which in turn stimulates matrix metalloproteinases (MMPs) and reactive oxygen species (ROS) synthesis. These enzymatic and oxidative mediators cleave major structural EG components, such as syndecans and heparan sulfate, from the endothelial surface (14). The resulting fragments enter the circulation, where they act as alarmins, amplifying inflammation through further cytokine release (15, 16). Concurrently, EG degradation exposes proteoglycans on the endothelial surface layer, activating coagulation (6). Excessive coagulation leads to microthrombosis, impairing oxygen and nutrient delivery to the tissues (4). Catecholamine surges in shock states further exacerbate EG damage by stimulating MMP and ROS production, a phenomenon known as shock-induced endotheliopathy (17). Similarly in kidney disease, circulating uremic toxins contribute to EG damage, although the exact mechanism is not fully understood (12, 18, 19).

Beyond propagating inflammation and coagulation, EG damage increases vascular permeability, leading to interstitial oedema, further impairing oxygen delivery and contributing to multi-organ dysfunction (20). These effects can be compounded by intravenous (IV) fluid administration, which is a cornerstone of supportive care in both human and veterinary critical care (21, 22), despite the risk for iatrogenic fluid overload (5, 7). Numerous experimental and clinical studies have investigated the impact of various regimens of IV fluid administration on the EG with conflicting results (23–32). Whether IV fluids independently cause damage to the EG or exacerbate pre-existing injury remains debated (5, 7, 33).

In veterinary medicine, clinical studies investigating EG injury are limited. Some studies have reported increased circulating EG constituents in septic adult horses (34) and foals (35), while marginal increases have been observed in cats with hemotropic mycoplasma (36). Other studies have found no changes in cats with head trauma (37) or dogs undergoing cardio-pulmonary bypass (38). However, reliance on shed EG constituents as biomarkers has inherent limitations: their concentrations are influenced by variable synthesis rates, turnover, and renal clearance (7, 39–43), making them challenging to interpret in the clinical setting.

An alternative method for *in vivo* assessment of the microcirculation is sidestream dark field videomicroscopy (SDFV), which can be combined with the proprietary software GlycoCheck™ to estimate EG thickness (44). This technique has been applied in numerous human studies, primarily in sepsis, to explore its effects on the microvasculature (12, 44–51). In veterinary research, GlycoCheck™ has been thus far used predominantly in the experimental setting (29, 52–57), except for one clinical study in dogs undergoing cardio-pulmonary bypass (38). The technique has been used to establish tolerance intervals (prediction-based intervals suited for repeated measurements or devices rather than population-derived biomarkers) in a healthy population of cats (56) and to evaluate the effects of IV fluid administration in healthy cats under experimental conditions ((55), abstract version (57), full manuscript in review). To date, however, no studies have assessed GlycoCheck™ parameters in cats with naturally occurring disease.

Considering the challenges of translating experimental protocols to the clinical setting, the aims of this exploratory study were to assess feasibility of obtaining GlycoCheck™ measurements in hospitalized cats, identify potentially suitable clinical cohorts, and determine optimal data collection timepoints for future multi-center studies. In addition, this study sought to examine potential associations of GlycoCheck™ parameters, markers of disease severity and IV fluid administration, with results intended to inform power calculations and guide design of future research.

## Materials and methods

### Study population

This was a prospective, single center, observational exploratory study, approved by Massey University’s Animal Ethics Committee (MUAEC Protocol 21/77). Cats admitted to the Massey University Companion Animal Hospital with trauma or non-traumatic illness were enrolled between March 2023 and March 2024 after obtaining written owner consent. The study adhered to the Animal Research Reporting of *In Vivo* Experiments guidelines (58). Cats were eligible if they were admitted for trauma or had signs of non-traumatic systemic illness on initial diagnostic work up and had sedation or general anesthesia planned within 24 h of admission (after stabilization, if required) for diagnostic or therapeutic procedures. Cats were excluded if they were deemed too unstable to undergo sedation or general anesthesia at the primary clinician’s discretion or were < 3 months of age.

### Data collection

Recorded variables included age, weight, diagnosis (or suspected diagnosis if definitive diagnosis not available), length of hospitalization (LOH) and outcome (survival to discharge). Variables related to IV fluids administration included whether the cats received IV fluids prior to data collection, IV fluid type, IV fluid challenge (IV fluid bolus administered to assess fluid responsiveness by evaluating changes in hemodynamic variables before and after infusion) volume (ml) during resuscitation, IV fluid hourly rate (ml/h), total volume (ml) and duration (h). Each cat was assigned a feline Acute Patient Physiologic and Laboratory Evaluation fast score (APPLE_fast_; score range 0–50) with data obtained within 24 h of admission (59). In addition, cats with trauma were assigned an Animal Trauma Triage score (ATT; score range 0–18) (60). Wherever multiple entries were available, the most adverse value was used for scoring as performed during the original development of the APPLE score (59) and the closest to admission for the ATT score (60). Packed cell volume (PCV) was measured as part of the APPLE_fast_ score. Serum amyloid A was also measured as a marker for systemic inflammation.

### Image acquisition

All image acquisitions were performed by one of two experienced operators with the animals sedated enough to obtain jaw relaxation or under general anesthesia. Cats were placed in lateral recumbency, and the base of the tongue exposed such as the sublingual mucosa was accessible for the videomicroscope as previously described (56). A hand-held SDFV camera connected to a laptop with GlycoCheck™ software (MicroVascular Health Solutions, USA) was placed in contact with the sublingual mucosa by the operator. Three measurements per datapoint were obtained in accordance with current recommendations (61, 62).

### GlycoCheck™ analysis

**Figure 1:**
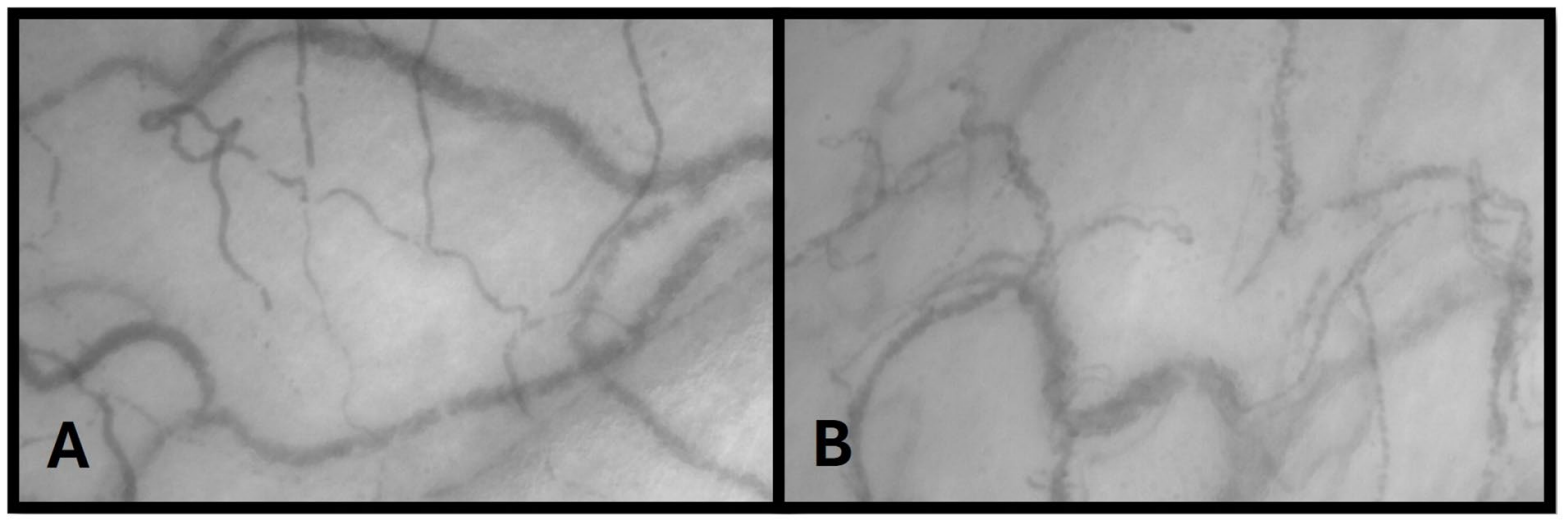
Sidestream dark field videomicroscopy image obtained from the sublingual region of a cat with trauma **(A)** and with sepsis **(B)**. Capillaries appear dark on a bright background. Note: these are intended for general visualization and not detailed microscopic comparison.

The method for measuring and calculating GlycoCheck™ parameters has been previously described (56). Briefly, the SDFV camera emits green light at 540 nm wavelength which is absorbed by hemoglobin in circulating red blood cells (RBC). Therefore, vessels appear dark on a bright background (Figure 1).

A video recording is displayed on the screen, and the operator can perform a real-time quality check including focus, pressure and motion artifacts (63). Furthermore, the software assesses and adjusts for motion and focus and records images only if optimal conditions are present. Each measurement is performed from approximately 10 video recordings containing 40 frames each (44). The software automatically identifies vessels that are less than 30 μm in diameter by analysing the contrast between the RBC and the background. The software then subdivides vessels into major (10 μm) and subsequently minor (0.5 μm) vascular segments in length and further determines the percentage of minor segments within a major segment with sufficient contrast for analysis.

A total of approximately 3,000 major vascular segments including vessels ranging from ~ 3–30 μm diameter are recorded for analysis during each measurement. The counts for major vascular segments of each analyzed diameter are additionally recorded. Major segments with > 60% of minor segments with sufficient contrast are considered valid vascular segments (VVS). Valid vessel density (VVD) per mm^2^ is then expressed by multiplying 10 μm by the number of valid vascular segments identified.

Next, RBC column widths are assessed for minimal column width, column position, and signal-to-noise ratio. From the measured column widths, the software then calculates the percentage of RBC filling (%RBC filling) based on the proportion of time a particular VVS contains RBC throughout all recordings per site. The median RBC column width (Median P50) is calculated using the intensity profiles of the dynamic lateral positions of RBCs in all measured column widths. Finally, a linear regression curve is fitted to the Median P50 slope to derive the perfused diameter of vascular segments.

From that, the perfused boundary region (PBR), an inverse estimate of EG thickness, is calculated as the distance between the Median P50 and the perfused diameter [(Perfused diameter – median RBC column width)/2]. Reported GlycoCheck™ parameters, therefore, include VVD, %RBC filling, Median P50 and PBR for vessels with diameter from 5 to 25 μm and for subgroups of vessels with diameters of 5–9 μm, 10–19 μm and 20–25 μm. Higher PBR is, therefore, an indication for a thinner EG and lower PBR indicates a thicker EG.

### Statistical analysis

This study was exploratory, and no formal power-driven sample size calculation was required. Nonetheless, an approximate sample-size estimate was conducted to satisfy ethical requirements and to demonstrate feasibility. Based on similar studies in critically ill people, the difference in PBR 5–25 μm between live and dead cats was estimated to be least 0.4 μm (50). The standard deviation in a previous study of healthy cats was 0.26 μm (56). Thus, with an effect size of d = 0.4/0.26 = 1.54, this study would require at least 8 cats per group (alive vs. dead) to detect that difference, with a power of 80% and α = 0.05. Based on an approximate mortality rate of 20% for critically ill cats, 40 cats would need to be enrolled to ensure sufficient cats that die from their disease (20% of 40 ≂ 8). This estimate remains speculative in the absence of appropriate species-specific prior data to inform the calculations. Therefore, it should be interpreted as illustrative rather than inferential.

Triplicates from measured GlycoCheck™ parameters were averaged for further analysis as recommended in previous reports in healthy and critically ill people (61, 62). Normality was analyzed using Shapiro–Wilk test and direct visualization with histograms (64, 65). Normally distributed data was presented as mean ± standard deviation and not normally distributed data as median (interquartile range) (66). Equivalent linear models were initially fitted as sensitivity analyses and yielded comparable effect directions (67). However, generalized linear models with a Gamma distribution and log link were retained for inference due to the strictly positive nature of GlycoCheck™ parameters and improved residual behaviour (67). These were used to evaluate associations between clinical variables and PBR, as well as other GlycoCheck™ parameters. One model included APPLE_fast_, LOH, and clinical group (trauma or non-traumatic) as predictors. A second model was restricted to cats that received IV fluids and included total IV fluid volume, rate and duration as predictors. A third model assessed the effect of ATT within the trauma subgroup. Reported 95% confidence intervals were derived from profile likelihood. Statistical significance was defined as *p < 0.05*. Linear models with single predictors were used for *post hoc* power and sample-size calculations, as reliable analytical power calculations for Gamma-distributed GLMs are not readily available. These provide approximate estimates for illustrative quantification. Vascular segment count distributions were compared across diameter categories using Friedman’s test and Wilcoxon-signed ranking tests. All statistical analyses were conducted in R (Team, 2023) (67).

## Results

### Clinical data

Nineteen cats were included for analysis, 11 with trauma and 8 with non-traumatic illness. The cats with non-traumatic illness included two with sepsis (pyothorax diagnosed based on history, physical exam findings and clinic-pathological abnormalities consistent with systemic inflammation and a pleural effusion cytology and culture), two with mucometra progressing toward pyometra with mild systemic signs, three with severe azotemia (two with complicated urethral obstruction and one with acute kidney injury of unknown origin), and one with mediastinal lymphoma. Individual patient data is presented in Table 1. Of the cats with trauma, five were presumed to have sustained road traffic injuries, two were injured in dog attacks, one in a cat fight, one after being caught under a garage door, and one had an unknown cause. Trauma severity was classified as severe (ATT ≥ 3) in nine cats (68).

**Table 1:** Individual patient data: diagnoses, outcomes, severity of illness scores, and selected clinical variables.

ID	Diagnosis	Detail	LOH	Outcome	APPLE_fast_	ATT	PCV	SAA
1	Trauma	Subcutaneous emphysema, Pneumothorax, Pneumomediastinum, Pneumoretroperitoneum	4	Discharged	17	4	45	-
2	Azotemia	Urethral obstruction	9	Discharged	17	-	39	128
3	Pyothorax	Pyothorax	10	Discharged	24	-	48	-
4	Pyothorax	Pyothorax	7	Discharged	15	-	28	77
5	Trauma	RTA, tail pull injury	7	Discharged	19	4	33	-
6	Trauma	RTA, stifle instability, open wounds	2	Discharged	13	5	39	102
7	Trauma	Dog attack, extensive degloving injuries	0	Euthanized (financial)	15	6	-	60
8	Trauma	Caught under garage door	1	Discharged	17	4	39	10
9	Trauma	RTA, Bilateral femoral Fx	4	Discharged	13	4	18	196
10	Gastrointestinal hemorrhage, Acute kidney injury	Severe anemia, melena, azotemia following NSAIDs	7	Discharged	8	-	13	61
11	Trauma	Fibula Fx	2	Discharged	13	1	28	75
12	Trauma	RTA, ileum Fx, open wounds	6	Discharged	21	6	37	85
13	FeLV Lymphoma	Mediastinal mass, pleural effusion	1	Discharged	24	-	34	1
14	Mucometra	Progressing to pyometra	1	Discharged	16	-	24	10
15	Trauma	Dog attack, wounds, urethral tear	5	Discharged	23	4	21	90
16	Trauma	RTA; TBI, mandibular symphyseal Fx, lung contusions	3	Discharged	22	5	46	-
17	Azotaemia	Urethral obstruction	4	Discharged	14	-	40	54
18	Trauma	Multiple cat bites	0	Discharged	15	2	36	76
19	Mucometra	Progressing to pyometra	1	Discharged	18	-	23	11

APPLE_fast_, feline acute patient physiologic and laboratory analysis score (fast) (score range 0–50); ATT, animal trauma triage score (score range 0–18); Fx, fracture; LOH, length of hospitalization (days); NSAIDs, non-steroidal anti-inflammatory drugs; PCV, packed cell volume (%); RTA, road traffic accident; SAA, serum amyloid A (mg/L); TBI, traumatic brain injury.

One cat was excluded for incomplete GlycoCheck™ analysis and another for missing clinical data. Success rate for obtaining measurements was 95%. Survival to discharge was 95%. One cat was euthanized due to financial constraints after trauma. Two cats had measurements obtained after >24 h from admission (36 and 40 h respectively, due to delays in anesthesia planning). Six cats were considered anemic (PCV < 28%). Summary statistics for recorded demographic and clinical variables are presented in Table 2 and per group in Supplementary Table 1.

**Table 2:** Demographic and clinical variables for 11 cats with trauma and eight cats with non-traumatic disease enrolled for GlycoCheck™ analysis.

Variable	Value
Age (years)	2.00 (0.83–8.84)^#^
Weight (kg)	4.26 (2.90–5.31)^#^
LOH (days)	3.89 ± 3.07
APPLE_fast_	17.05 ± 4.29
ATT^⸹^	2.00 (0–4.00)^#^
SAA (mg/L)^⸸^	69.10 ± 51.30
PCV (%)^¶^	32.8 ± 10.1
IV fluid challenge (ml)*	50 (40.75–57.5)^#^
IV fluid rate (ml/h)*	11.65 ± 7.38
IV fluids duration (h)*	18.7 ± 11.90
IV fluids total (ml)*	126 (115–333.25)^#^

APPLE_fast_, feline acute patient physiologic and laboratory analysis score (fast) (score range 0–50); ATT, animal trauma triage score (score range 0–18); LOH, length of hospitalization; PCV, packed cell volume; SAA, serum amyloid A.

#Not normally distributed and presented as Median (Interquartile Range); ⸹From subset of cats with trauma (*n* = 11); ⸸*n* = 15; ¶*n* = 18; *From subset of cats that received IV fluids (*n* = 10).

Ten cats received IV fluids with four of these cats receiving IV fluid challenges as part of their initial stabilization plan. The IV fluid type consisted almost exclusively of Lactated Ringer’s solution (LRS). One cat received a blood transfusion. No synthetic colloids were used. All anesthetized cats received total IV anesthesia. No vasoactive agents were used during image acquisition. Type of anesthetic intervention, anesthetic agents, timing of data collection in relation to anesthesia and related procedures are presented in Supplementary Table 2.

Normally distributed data included PCV, LOH, APPLE_fast_, IV fluid rate and duration, and not normally distributed data – age, weight, ATT, IV fluid challenge and IV fluid total volume. A statistical report is included as Supplementary material.

### GlycoCheck™ data

**Figure 2:**
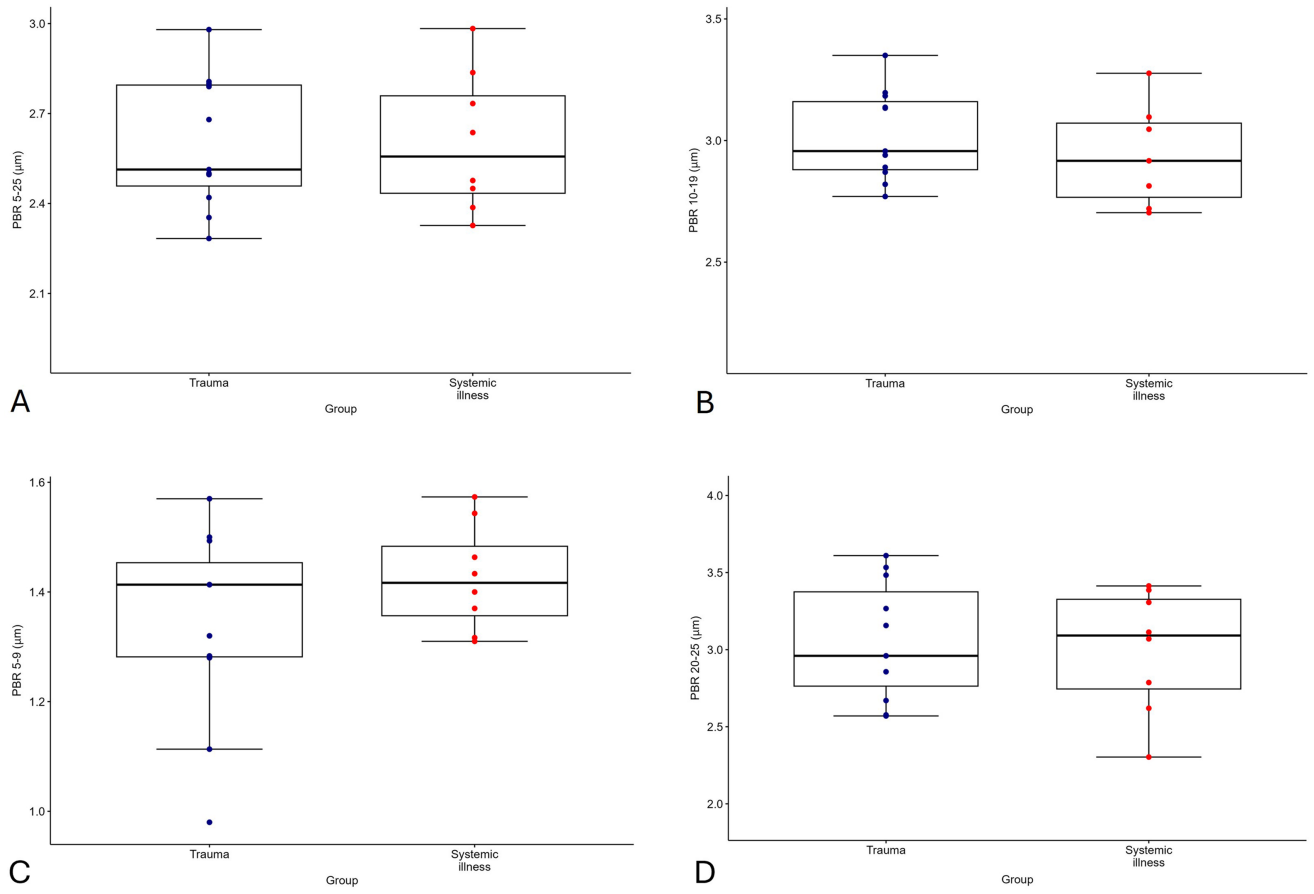
Box plot of perfused boundary region (PBR) values for vessels 5–25 μm **(A)**, 5–9 μm **(B)**, 10–19 μm **(C)**, and 20–25 μm **(D)** in diameter, showing individual data points for cats with trauma (*n* = 11, navy) and non-traumatic systemic illness (*n* = 9, red). The x-axis is scaled according to species-specific tolerance intervals.

GlycoCheck™ parameters VVD, %RBC filling, Median P50, and PBR were normally distributed. Summary statistics for GlycoCheck™ parameters are presented in Table 3 and per group in Supplementary Table 1 and visualized as box plots in Figure 2.

**Table 3:** Glycocheck™ parameters for 11 cats with trauma and eight cats with non-traumatic disease compared to previously established tolerance intervals.

Parameter	Mean	SD	95% CI	TI^56^
VVD μm/mm^2^	385.75	112.18	331.69–439.82	73.33–333.33
RBC filling %	59.00	8.00	55.00–62.00	59.85–85.07
Median P50 μm	8.26	1.12	7.71–8.79	5.63–8.59
PBR 5–25 μm	2.60	0.22	2.50–2.71	1.89–3.00
PBR 5–9 μm	1.38	0.15	1.31–1.45	0.97–1.58
PBR 10–19 μm	3.02	0.22	2.91–3.12	2.11–3.48
PBR 20–25 μm	3.03	0.38	2.85–3.22	1.87–4.02

PBR, perfused boundary region; RBC, red blood cells; TI, tolerance intervals; SD, standard deviation; VVD, valid vessel density.

There were no statistically significant effects of group (trauma or non-traumatic disease), LOH, ATT (for trauma group) and APPLE_fast_ on GlycoCheck™ parameters. For the 10 patients receiving IV fluids, none of the IV fluid variables had a statistically significant effect on PBR. IV fluid rate had a statistically significant positive effect on VVD (mean ratio = 1.07, 95% CI 1.02–1.13, *p* = 0.038) and a negative effect on Median P50 (mean ratio = 0.98, 95% CI 0.97–0.99, *p* = 0.031). IV fluid duration showed a negative effect on Median P50 (mean ratio = 0.98, 95% CI 0.97–0.99, *p* = 0.018), while the total volume had a positive effect on Median P50 (mean ratio = 1.001, 95% CI 1.000–1.002, *p* = 0.027). Packed cell volume had a significant effect on VVD (mean ratio = 1.02, 95% CI 1.00–1.03, *p* = 0.044), but not on other GlycoCheck™ parameters. Sample size estimates for effects on selected clinical variables on PBR 5–25 μm are presented in Table 4. The PBR in all cats were within previously established tolerance intervals for the species (56).

**Table 4:** Sample size estimates required to detect associations between PBR 5–25 μm and selected clinical variables in linear models with a single predictor for illustrative purposes only.

Variable	Model R^2^	Cohen f^2^	Total sample size	Number of observations
PCV	0.011	0.011	729	19
LOH	0.005	0.005	1,564	19
APPLE_fast_	0.029	0.030	256	19
ATT^¶^	0.029	0.030	256	11
IV fluids rate*	0.153	0.181	46	10

Calculations assume a power of 0.80 and a significance level of 0.05. Effect sizes are expressed as model R^2^ and corresponding Cohen’s f^2^. Total sample size indicates the estimated number of subjects needed; the number of observations reflects the current dataset.

APPLE_fast_, feline acute patient physiologic and laboratory analysis score (fast) (score range 0–50); ATT, animal trauma triage score (score range 0–18); IV fluids rate – intravenous fluid rate (ml/kg/h); LOH – length of hospitalization (days); PCV, packed cell volume (%).

^¶^From subset of cats with trauma (*n* = 11).

*From subset of cats that received IV fluids (*n* = 10).

**Figure3:**
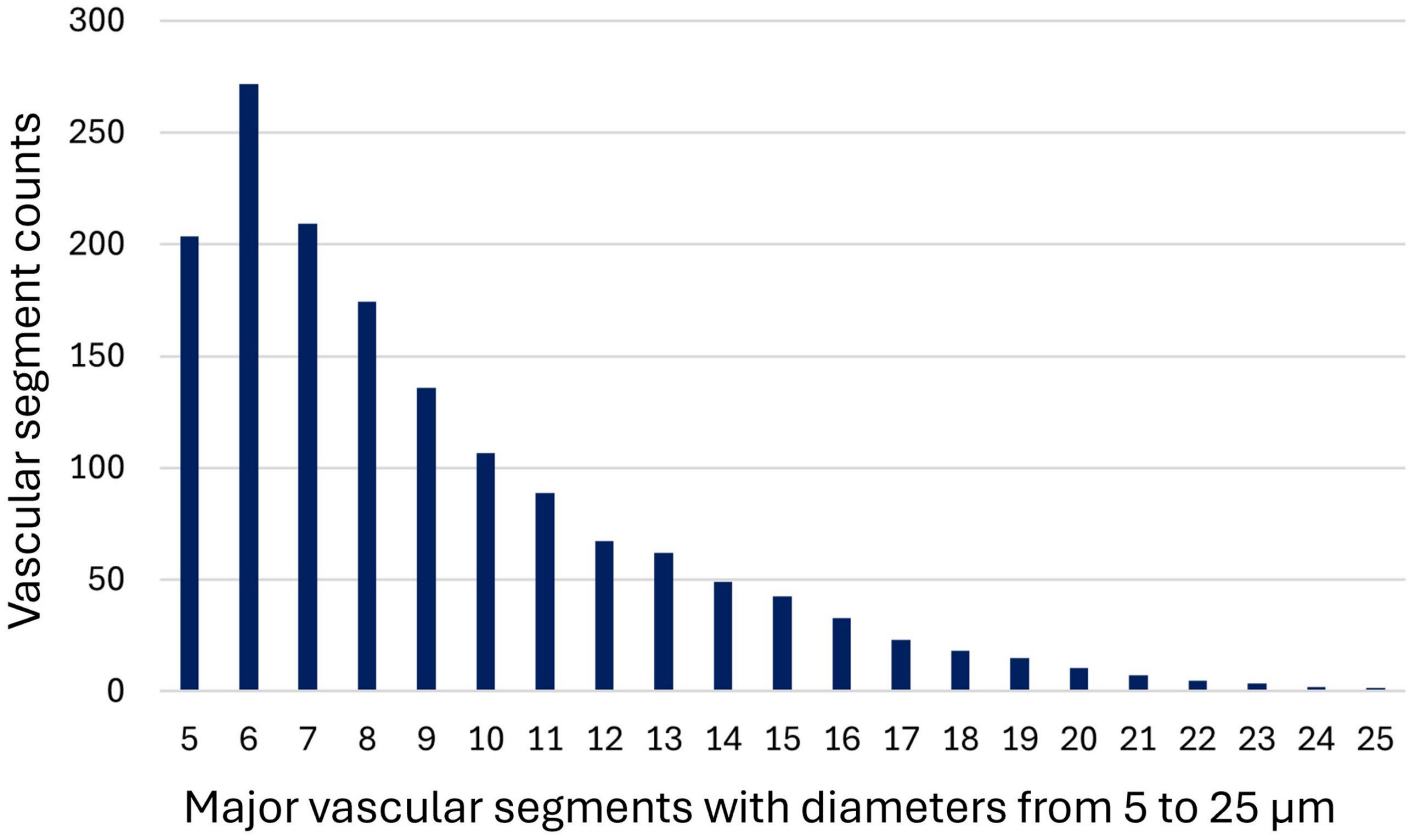
Distribution of major vascular segment counts by vessel diameter (5–25 m) as measured using GlycoCheck™ analysis in 19 cats with naturally occurring disease (trauma, *n* = 11; non-traumatic illness, *n* = 8). Bars represent the number of valid vascular segments identified per diameter subgroup.

The count for vascular segments with diameters 5–25 μm was 1529.42 (±356.04), and for the subgroups with diameter 5–9 μm, 994.42 (±350.76); 10–19 μm, 505.05 (± 171.65) and 20–25 μm 29.94 (±19.38), respectively. The distribution of vascular segment counts from 5 to 25 μm is presented in Figure 3. There was a statistically significant difference between all the vascular count subgroups (*p <* 0.001 for all comparisons).

## Discussion

This exploratory study demonstrates that SDFV image acquisition and EG thickness estimation using GlycoCheck™ can be reliably performed in critically ill cats. Clinical variables reflecting illness severity, APPLE_fast_ and ATT scores, LOH and type of illness (trauma vs. non-traumatic) had no effects on GlycoCheck™ parameters. Minor and inconsistent effects of IV fluid variables on source GlycoCheck™ parameters were observed. Additionally, a significant difference was found in the distribution of vascular segment subgroups contributing the overall PBR 5–25 μm measurement, highlighting the importance of examining vessel size breakdown.

The study cohort comprised 19 critically ill cats, including a relatively homogeneous trauma group and a more heterogeneous non-traumatic illness group. Trauma cases predominantly involved blunt or penetrating injury of varying severity, whereas non-traumatic cases encompassed a broad range of systemic illnesses with differing pathophysiology and illness severity, including sepsis, reproductive disease, renal dysfunction, and neoplasia. This heterogeneity reflects the case mix of a tertiary referral hospital and was an inherent feature of this exploratory feasibility study. While this diversity enhances generalisability, it may also have contributed to variability in microcirculatory measurements and limited the detection of condition-specific effects.

No associations were found between GlycoCheck™ parameters and selected clinical outcome variables. This lack of detectable effects is likely multifactorial. First, the small sample size limited the statistical power to identify significant associations. *Post hoc* sample size calculations confirmed that this study was underpowered to detect meaningful effects of selected clinical variables, such as APPLE_fast_ and ATT scores, LOH, PCV, IV fluids rates on PBR 5–25 μm - the summary parameter for estimated EG thickness across vascular diameters. These calculations provide useful illustrative guidance for future study design but should be interpreted with caution, as they were based on simplified models. Furthermore, patient characteristics, disease severity, hospitalization practices, and outcomes may differ across institutions and regions affecting such calculations.

Another possible explanation for the lack of effects, is that the enrolled cats, either affected by trauma or non-traumatic illness, did not experience sufficient severity of illness to cause significant damage to the EG. The mean APPLE_fast_ score of 17 in this cohort corresponds to an estimated mortality rate of 6.35% (59), and the median ATT score of 2 in the trauma subgroup aligns with a predicted mortality rate of approximately 7% (60), based on the original score validation studies. It is plausible that more severely ill cats were either too unstable to undergo general anesthesia or were euthanized shortly after admission due to poor prognosis and financial constraints. This selection bias is supported by previous feline trauma studies reporting early euthanasia decisions driven by financial limitations (69–71). Moreover, global data consistently show that owners spend less on cats than dogs in veterinary care, which may further explain the inclusion of less severely ill cats (with potentially better prognosis for a given expenditure) in this cohort (72–74). The center where the study was performed is in a region facing socio-economic challenges at the time of data collection, potentially exacerbating a financial bias. Therefore, the relatively low severity of illness among enrolled patients in this study likely reflects both medical and financial considerations.

Finally, the lack of effects of severity of illness (or injury) on GlycoCheck™ parameters, may reflect limitations inherent to the severity of illness scores themselves. Severity of illness scores require extensive external validation to ensure performance outside their original development setting. While the ATT score has undergone such validation (60), the APPLE score has not, and its use in other populations should be interpreted with caution. Together, these factors highlight the complexity of applying standardized illness metrics to GlycoCheck™ data and the need for larger, multicenter studies with robust case diversity or focusing on specific cohorts with higher illness severity (for example septic cases).

In contrast with the absence of effects of severity of illness variables on GlycoCheck™ parameters, this study demonstrated some minor and varying effects of IV fluid administration on source GlycoCheck parameters VVD and median P50, without a consistent directional pattern. The significance of these remains unknown. They may represent type I errors arising from model instability associated with small sample size (only 10 cats received IV fluids) and the inclusion of multiple covariates. Notably, the confidence intervals around the effect estimates were relatively narrow, suggesting that any true effects of IV fluid variables on GlycoCheck™ parameters are likely small in magnitude.

In this study IV fluid administration variables had no effect on PBR. This contrasts human medicine studies where IV fluid-related increases in PBR have been observed in both stable surgical patients (33) and critically ill patients (7). However, results are largely inconsistent and changes often marginal. In addition, most studies in people, have focused on EG shedding biomarkers, (such as syndecan-1), rather than GlycoCheck™ analysis, complicating comparisons.

In veterinary medicine, published studies evaluating the impact of IV fluids on GlycoCheck™ parameters are limited to the experimental setting with reports from rabbits (53, 54), dogs (29, 52) and cats ((55), abstract version (57), full manuscript in review). These generally demonstrate changes in PBR only when large volumes are administered, typically following a prior insult, such as hemorrhage. In a series of studies in heathy anesthetized cats conducted by this group, IV fluid administration was associated with a marginal protective effect on estimated EG thickness, with lower PBR values observed in the intervention groups, compared to controls ((55), abstract version (57), full manuscript in review). The absence of a detectable effect of IV fluids on PBR in the present study should be interpreted with caution, as limited statistical power may have masked subtle associations. In addition, measurements were obtained at a single timepoint, which constrains interpretability compared with more robust before-and-after designs. Taken together, these findings underscore the complexity of the relationship between IV fluid administration and EG injury, for which no single explanatory model is likely to apply. Observed effects may be species-specific and dependent on patient population, study design, and research setting; therefore, direct extrapolation should be avoided. To specifically investigate the effects of IV fluids in sick cats, future prospective, adequately powered studies focusing on before-and-after effects and incorporating species-specific volume kinetics principles are required.

Building on the interpretation of GlycoCheck™ data, this study also highlighted important methodological considerations related to vascular segment distribution within the analysis. The GlycoCheck™ software detects vessels up to 30 μm in diameter, subdividing them into major vascular segments of 10 μm length based on sufficient image contrast. Up to 3,000 segments may be analyzed per scan, with only those passing internal quality checks contributing to the calculation of PBR. In the current study, these 10 μm vascular segments (vascular segment counts) were unevenly distributed across diameter ranges with a marked overrepresentation (~60%) of vessels measuring 5–9 μm in diameter. This disproportion was statistically significant compared to segments from 10–19 μm and 20–25 μm vessels, a finding consistent with previous reports in both feline studies (57) and human cohorts, including patients with pre-eclampsia (75) and type 2 diabetes (76).

This uneven representation is noteworthy for two key reasons. First, the PBR values for 5–9 μm vessels are approximately twofold smaller than the other PBRs in cats, dogs and people (56). When this subgroup dominates the overall PBR 5–25 μm calculation, the resulting average may underrepresent actual EG thinning, potentially skewing interpretation. Second, different vessel sizes may respond differently to physiological and pathological stimuli. For instance, in a previous feline study from this research group, PBR 5–9 μm decreased following transient volume overload in cats, while other PBRs did not (57). These variations suggest that interpreting only the average across all vessel sizes may obscure meaningful subgroup-specific changes.

Despite this, most veterinary studies continue to report only the overall PBR 5–25 μm, potentially overlooking important microvascular dynamics (29, 38, 52–54). Since the distribution of vascular diameters reflects the inherent anatomy of the sublingual mucosa, this discrepancy is an unavoidable feature of the analysis. As such, methodological approaches should account for it. Future studies are encouraged to report PBR values stratified by vessel diameter to provide a more detailed understanding of EG responses and enhance clinical interpretation.

Another potential methodological consideration is the effects of PCV on GlycoCheck™ parameters. In this study lower PCV was associated with decrease in VVD. This finding aligns with previous experimental work from this group, which demonstrated associations between decreasing PCV and changes in a number of GlycoCheck™ parameters, including increases in PBR values ((55), abstract version (57), full manuscript in review). Similar trends have been observed in human studies; one in healthy individuals with different ethnicities reported lower PBR with higher PCV (77), while a study in neonates, found increased PBR in the pre-term cohort with lower PCV (78).

All GlycoCheck™ parameters are derived based on identification of RBCs on a contrasting background. Therefore, hemodilution from anemia may reduce contrast and affect measurement reliability. In the present study, only VVD was significantly affected by PCV. This may reflect the limited number of anemic cats (*n* = 6), reducing power to detect changes in other parameters. In contrast, the anesthetic protocol and IV fluid administration in the above-mentioned feline experimental studies induced more pronounced hemodilution. Notably, VVD appeared to be the most consistently PCV-sensitive parameter across studies ((55), abstract version (57), full manuscript in review). Additionally, PCV and GlycoCheck™ measurements were precisely paired in the experimental work, whereas timing differed in the clinical setting. These factors may explain the discrepancy in findings between the experimental and clinical studies. Further clinical studies are needed to define the magnitude and clinical relevance of PCV effects on GlycoCheck™ parameters and to guide appropriate interpretation.

The GlycoCheck™ software was developed for use in human subjects. The analyzed range of vascular diameters (5–25 μm) broadly corresponds to what is defined by experts as “microvessels” (<20 μm), encompassing arterioles, capillaries, and venules, with capillaries measuring <10 μm in diameter (63, 79, 80). However, this classification is based on human microvascular anatomy, and does not explicitly account for anatomic or functional inter-species differences. Such inter-species differences may influence both the analytical performance of the system and the interpretation of parameters related to capillary function. One example is valid vessel density (VVD), which appears to be substantially lower in cats compared with dogs and people (44, 56, 81). Whether this reflects true anatomical and physiological differences or limitations in software performance when applied to feline microcirculatory images remains unclear. In support of genuine inter-species differences, cats have a lower blood volume than dogs and people (approximately 55 mL/kg lean body weight) (82) and subjectively appear to have a less dense sublingual capillary network under a videomicroscope (personal observation). Differences in capillary bed density have been reported across mammalian species (83) including studies using SDFV to assess perfused vessel density in cats, dogs, and horses (84–87). Conversely, analytical performance may also contribute. Feline hemoglobin exhibits lower oxygen affinity (88), while maintaining similar oxygen content compared with other species (89). Although subtle, these differences may influence image-based software analysis. In addition, lower capillary density would result in fewer detectable vessel segments and datapoints, potentially increasing measurement noise and variability. Consistent with this, moderate intra- and inter-subject variability has been reported in a large cohort of healthy cats (*n* = 101) (56). However, since human studies have also demonstrated variability, this may be an analytical limitation rather than a species-specific difference (61, 62, 90). Accordingly, until comparative validation data across species become available, results should be interpreted with caution and direct inter-species comparisons avoided. Large samples sizes and robust study designs, allowing replicability would improve within-species result interpretation and therefore, integration of knowledge into practice.

Circulating biomarkers represent an alternative approach for assessing EG damage in the clinical setting, considering the above-mentioned potential limitations of the GlycoCheck™ software. However, these were not measured in this study. To the authors’ knowledge, hyaluronan is the only biomarker validated for use in feline patients (91). However, it lacks specificity for the EG, as it is abundant in the interstitium (92). This limits its interpretability, particularly in conditions associated with IV fluid administration where interstitial “washout” leads to increased plasma concentrations (93). Two studies have reported the use of “feline” syndecan-1 enzyme-linked immunosorbent assays (36, 37). However, these assays do not appear to be species-specific and are labelled as feline based on assumed homogeneity (personal communication), and publicly available information regarding their validation in cats is limited.

These limitations reflect a broader challenge associated with EG biomarkers, as studies in people and dogs frequently report inconsistent or marginal associations. Experts have questioned their utility given the variability in biomarker synthesis, shedding, turnover, renal clearance, and the extent to which circulating concentrations accurately reflect biologically relevant EG injury (7, 39–43). For example, surface markers such as syndecan-1 may indicate structural disruption (94), whereas junctional markers such as vascular endothelial cadtherin-1 may reflect functional impairment (95). Taken together, these considerations warrant caution when designing or interpreting feline studies relying on EG damage biomarkers. Development of species-specific assays, robust assay validation and mostly improved understanding of the composition and behaviour of the feline EG should precede their broader application in clinical research.

This study assessed the feasibility of prospectively collecting GlycoCheck™ data in critically ill cats. Image acquisition and analysis proved practical and safe, and data collection could be integrated into the clinical workflow. However, several pragmatic adaptations to the study design were necessary to ensure successful data collection. A relatively broad time window for image acquisition was permitted, which may have introduced variability in the degree of microcirculatory alteration and PBR measurements. Measurements obtained closer to admission might have better captured EG injury in sick cats. However, this was not feasible due to the need for initial patient stabilisation, multidisciplinary planning, obtaining owner consent among other considerations.

Only cats requiring sedation or general anesthesia were eligible for inclusion, resulting in the exclusion of a substantial proportion of admitted patients, potentially those that were more severely ill. This resulted in a relatively homogeneous trauma cohort and a more heterogenous non-traumatic cohort, reflecting the facility’s caseload. In addition, longitudinal data collection was not feasible, largely due to owner reluctance to consent to sedation or general anesthesia solely for image acquisition when no additional interventions were planned. Although efforts were made to acquire images at the onset of sedation whenever possible, this could not always be achieved, as urgent clinical procedures (often emergency department–led imaging or wound care) necessarily took priority. In contrast, image acquisition proved more practical at the start of general anesthesia, while the patient was being prepared and instrumented.

Based on these observations, future studies should aim to streamline image acquisition by targeting timepoints that are both clinically relevant to disease progression and as close to admission as feasible, standardising timing in relation to sedation and anesthesia, and exploring approaches that allow enrolment of patients irrespective of whether sedation or general anesthesia is planned, while including longitudinal data collection.

As a result of this adaptive exploratory design, this study has several limitations. The 24 h window for obtaining data was arbitrarily selected and not reflective of disease progression. Moreover, critically ill patients are often subject to multiple interventions during the initial 24 h, which may have influenced the measurements. The inclusion restricted to cats requiring sedation or general anesthesia may have introduced selection bias. Trauma patients are more likely to undergo anesthesia for imaging or surgery, further skewing representation. The true composition of the broader clinical cohort is unknown as records for non-enrolled cats, including those euthanized early, too unstable for anesthesia, or not pursued due to financial constraints were not kept. This potentially introduces a survival bias. As a result, a survival analysis could not be performed (the only non-survivor was a result of a financially driven euthanasia). The study cohort, particularly the non-traumatic group, was heterogenous with respect to illness severity. In combination with the arbitrary timing of sample collection, this heterogeneity may have increased variability and obscured potential effects. While all GlycoCheck™ parameters remained within established tolerance intervals for the species, these remain descriptive rather than determinative of clinical relevance. Lastly, only single measurements were obtained, not allowing for exploration of longitudinal effects.

The small sample size limited statistical power, and furthermore, the target of 40 subjects could not be reached for the duration of the study. This could have resulted in type II errors. Therefore, results from this study should be interpreted with caution and direct extrapolations to other populations of sick cats – avoided. Generalized linear modelling was selected based on the characteristics of the outcome variables, with predictors grouped into smaller, biologically and clinically plausible models rather than a single data-driven model, and proportional rather than absolute effects were plausible. Profile-likelihood inference was used to derive the reported 95% confidence intervals to reduce instability associated with the small sample size. Strictly mathematically, Gamma specification pertains to the assumed conditional mean–variance relationship, and not to the marginal distribution of the outcome (96). However, the limited sample size precluded formal modelling of the variance function, residual diagnostics and comparison with linear models supported the use of a Gamma–log framework in this exploratory setting (96). While this approach reduces model complexity, it may still carry a risk of overfitting in a small exploratory dataset. In larger datasets with longitudinal sampling, alternative analytical approaches such as generalized linear mixed-effects models, linear mixed models, and principal component analysis or trajectory-based modelling could be applied to better capture group differences and temporal patterns in microcirculatory parameters.

Anesthetic effects represent a potential confounder in small animal microcirculatory imaging studies, as deep sedation or general anesthesia is required for image acquisition. Anesthetic influences were deliberately not included within the aims of the present study, as the dataset was not suited to reliably address hypotheses related to anesthetic effects. Consequently, some degree of variability may have arisen from differences in anesthetic protocols. Previous studies have demonstrated no effect of alpha-2 agonists when added to a butorphanol and propofol containing protocol (56). However, two recent studies in feline patients undergoing a fluid challenge (55) and transient volume overload (abstract version (57), full manuscript in review) demonstrated increases in PBR over time in the control group receiving only propofol anesthesia. The effects of other anesthetic agents remain unknown.

The timing of image acquisition relative to anesthetic induction represents an additional potential source of bias. Measurements were obtained at variable timepoints during anesthesia, depending on clinical stabilisation and procedural priorities, and could not be standarized. Early measurements may have coincided with hemodynamic transitions and intravascular volume shifts, whereas later measurements may have reflected more stable but pharmacologically altered conditions. This variability in timing may have introduced additional noise and masked subtle microcirculatory changes, and its direction and magnitude could not be assessed within the current study design. Future research should therefore aim to establish protocols that minimise anesthetic effects and optimize timing of anesthesia in relation to image acquisition (e.g., intra-vascular volume effects may be more pronounced at the start (97, 98), while other effects may be more visible at the end of general anesthesia).

Finally, two operators performed GlycoCheck™ measurements. Although inter-operator variability for GlycoCheck™ measurements appears minimal in human studies (99), this has not been investigated in cats. It is also important to acknowledge that microvascular beds vary in composition and response to stimuli (100) and sublingual measurements may not fully represent systemic microcirculatory changes. Therefore, assessments from the sublingual capillary bed should be extrapolated to the remainder of the microcirculation with caution.

## Conclusion

This exploratory study demonstrates that obtaining GlycoCheck™ measurements is feasible in a diverse cohort of critically ill cats and can be integrated into routine clinical workflows. The largely neutral findings should not be interpreted as evidence against biological relevance, but rather as defining important limitations of sensitivity in clinically complex populations assessed at a single, variable timepoint. While minor associations with IV fluid variables and PCV were observed, the study primarily highlights how disease heterogeneity, timing of measurements, and study design constraints may obscure subtle microcirculatory effects. As such, GlycoCheck™ appears better suited, at present, as a research tool within controlled study designs than as a standalone diagnostic or prognostic instrument in feline critical care. Importantly, these findings provide practical design insights that will inform future studies aimed at translating GlycoCheck™ from the research setting into clinical practice.

## Data Availability

The raw data supporting the conclusions of this article will be made available by the authors, without undue reservation.

## Author’s note

Preliminary results from this study were presented as an oral abstract at the 30th International Veterinary Emergency and Critical Care Symposium in 2024.

## Supplementary material

SUPPLEMENTARY DATA SHEET 1 Statistical report - unformatted.

SUPPLEMENTARY TABLE 1 Timing and type of anaesthetic interventions and associated procedures.

SUPPLEMENTARY TABLE 2 Demographic variables and GlycoCheck-TM parameters per group.
