# Supplementary material for: Endothelial glycocalyx thickness in cats with naturally occurring trauma or non-traumatic illness: an exploratory study
Source: Front Vet Sci. 2026 Feb 23;13:1751034. doi: 10.3389/fvets.2026.1751034 (PMC12967996; doi:10.3389/fvets.2026.1751034)
Supplement: SUPPLEMENTARY DATA SHEET 1 — Statistical report - unformatted. [file Data_Sheet_1.pdf]

GlycoCheck-TM in critically ill cats
Statistical report

Distribution of data (normality)

Demographic and severity of illness variables

```
> variables <- c("Total_ATT", "Short_ATT", "Age_years", "Weight", "LOH",
+                "APPLE_fast",
+                "IV_fluid_challenge", "IV_fluids_rate", "IV_fluids_durati
on", "IV_fluids_total")
>
> for (var in variables) {
+   shapiro_test <- shapiro.test(Glycoheck_clinical_study_dataset_for_stat
s[[var]])
+
+   cat("Shapiro-Wilk test for variable", var, ":\n\n")
+   print(shapiro_test)
+   cat("\n\n")
+   ggplot(Glycoheck_clinical_study_dataset_for_stats, aes_string(x = var)
) +
+     geom_histogram(binwidth = 10, fill = "blue", color = "black", alpha
= 0.7) +
+     labs(title = paste("Histogram of", var), x = var, y = "Frequency") +
+     theme_minimal() +
+     theme(plot.title = element_text(hjust = 0.5))
+ }

Shapiro-Wilk test for variable Age_years :

        Shapiro-Wilk normality test

data:  Glycoheck_clinical_study_dataset_for_stats[[var]]
W = 0.81139, p-value = 0.001284



Shapiro-Wilk test for variable Weight :

        Shapiro-Wilk normality test

data:  Glycoheck_clinical_study_dataset_for_stats[[var]]
W = 0.92006, p-value = 0.1134



Shapiro-Wilk test for variable LOH :

        Shapiro-Wilk normality test

data:  Glycoheck_clinical_study_dataset_for_stats[[var]]
W = 0.92649, p-value = 0.1492



Shapiro-Wilk test for variable APPLE_fast :

        Shapiro-Wilk normality test

data:  Glycoheck_clinical_study_dataset_for_stats[[var]]
W = 0.95194, p-value = 0.426
```

Trauma subset

```
> shapiro_test <- shapiro.test(Glycoheck_clinical_study_trauma_dataset_for
_stats$Total_ATT)
>
> print(shapiro_test)

	Shapiro-Wilk normality test

data:  Glycoheck_clinical_study_trauma_dataset_for_stats$Total_ATT
W = 0.88213, p-value = 0.1107

> shapiro_test <- shapiro.test(Glycoheck_clinical_study_trauma_dataset_for
_stats$Short_ATT)
>
> print(shapiro_test)

	Shapiro-Wilk normality test

data:  Glycoheck_clinical_study_trauma_dataset_for_stats$Short_ATT
W = 0.94729, p-value = 0.6095
```

IV fluid variables subset

```
> filtered_data <- Glycoheck_clinical_study_dataset_for_stats[

+   Glycoheck_clinical_study_dataset_for_stats$IV_fluid_challenge > 0 |

+     Glycoheck_clinical_study_dataset_for_stats$IV_fluids_rate > 0 |

+     Glycoheck_clinical_study_dataset_for_stats$IV_fluids_duration > 0 |

+     Glycoheck_clinical_study_dataset_for_stats$IV_fluids_total > 0, ]

>

> iv_fluid_vars <- c("IV_fluid_challenge", "IV_fluids_rate",
"IV_fluids_duration", "IV_fluids_total")

> for (var in iv_fluid_vars) {

+   shapiro_test <- shapiro.test(filtered_data[[var]])

+   cat("Shapiro-Wilk test for variable", var, ":\n\n")

+   print(shapiro_test)

+   cat("\n\n")

+ }

Shapiro-Wilk test for variable IV_fluid_challenge :

      Shapiro-Wilk normality test

data:  filtered_data[[var]]

W = 0.7106, p-value = 0.001184


Shapiro-Wilk test for variable IV_fluids_rate :

      Shapiro-Wilk normality test

data:  filtered_data[[var]]

W = 0.82212, p-value = 0.02689
```

```
Shapiro-Wilk test for variable IV_fluids_duration :

      Shapiro-Wilk normality test

data:  filtered_data[[var]]

W = 0.9226, p-value = 0.3792


Shapiro-Wilk test for variable IV_fluids_total :

      Shapiro-Wilk normality test

data:  filtered_data[[var]]

W = 0.7628, p-value = 0.005109


> ggplot(filtered_data, aes(x = IV_fluid_challenge)) +

+   geom_histogram(binwidth = 1, fill = "blue", color = "black", alpha =
0.7) +

+   labs(title = "Histogram of IV_fluid_challenge (Filtered)", x =
"IV_fluid_challenge", y = "Frequency") +

+   theme_minimal() +

+   theme(plot.title = element_text(hjust = 0.5))

Warning message:

Removed 1 row containing non-finite outside the scale range
(`stat_bin()`).

> ggplot(filtered_data, aes(x = IV_fluids_rate)) +

+   geom_histogram(binwidth = 10, fill = "green", color = "black", alpha =
0.7) +

+   labs(title = "Histogram of IV_fluids_rate (Filtered)", x =
"IV_fluids_rate", y = "Frequency") +

+   theme_minimal() +

+   theme(plot.title = element_text(hjust = 0.5))

Warning message:

Removed 1 row containing non-finite outside the scale range
(`stat_bin()`).

> ggplot(filtered_data, aes(x = IV_fluids_duration)) +

+   geom_histogram(binwidth = 10, fill = "red", color = "black", alpha =
0.7) +

+   labs(title = "Histogram of IV_fluids_duration (Filtered)", x =
"IV_fluids_duration", y = "Frequency") +

+   theme_minimal() +

+   theme(plot.title = element_text(hjust = 0.5))

Warning message:

Removed 1 row containing non-finite outside the scale range
(`stat_bin()`).
```

```
> ggplot(filtered_data, aes(x = IV_fluids_total)) +
+   geom_histogram(binwidth = 50, fill = "purple", color = "black", alpha
= 0.7) +
+   labs(title = "Histogram of IV_fluids_total (Filtered)", x =
"IV_fluids_total", y = "Frequency") +
+   theme_minimal() +
+   theme(plot.title = element_text(hjust = 0.5))
Warning message:
Removed 1 row containing non-finite outside the scale range
(`stat_bin()`).
```

IV fluid variables summary statistics

```
> library(dplyr)
> library(openxlsx)
>
> iv_fluid_vars <- c("IV_fluid_challenge", "IV_fluids_rate",
"IV_fluids_duration", "IV_fluids_total")
> summary_stats <- data.frame()
>
> for (var in iv_fluid_vars) {
+   filtered_data <- Glycoheck_clinical_study_dataset_for_stats %>%
+     filter(.data[[var]] > 0)
+   stats <- filtered_data %>%
+     summarise(
+       Variable = var,
+       Mean = mean(.data[[var]], na.rm = TRUE),
+       SD = sd(.data[[var]], na.rm = TRUE),
+       Min = min(.data[[var]], na.rm = TRUE),
+       Max = max(.data[[var]], na.rm = TRUE),
+       Median = median(.data[[var]], na.rm = TRUE),
+       IQR = IQR(.data[[var]], na.rm = TRUE)
+     )
+   summary_stats <- bind_rows(summary_stats, stats)
+ }
> print(summary_stats)
            Variable  Mean        SD Min Max Median    IQR
1 IV_fluid_challenge 48.25 27.427176  13  80     50 16.750
2     IV_fluids_rate 11.65  7.378836   4  25      9  6.625
3 IV_fluids_duration 18.70 11.898179   2  40     17  7.000
```

```
4    IV_fluids_total 221.90 190.776454  50 620    126 218.25

> write.xlsx(summary_stats, file =
"Filtered_IV_Fluids_Summary_Statistics.xlsx", row.names = FALSE)

Warning message:

Please use 'rowNames' instead of 'row.names'

> cat("Summary statistics exported to
'Filtered_IV_Fluids_Summary_Statistics.xlsx'\n")

Summary statistics exported to
'Filtered_IV_Fluids_Summary_Statistics.xlsx'


# List of IV fluid-related variables

iv_fluid_vars <- c("IV_fluid_challenge", "IV_fluids_rate",
"IV_fluids_duration", "IV_fluids_total")

summary_stats <- data.frame()


for (var in iv_fluid_vars) {

  # Filter the data where the variable is greater than 0

  filtered_data <- Glycoheck_clinical_study_dataset_for_stats %>%

    filter(.data[[var]] > 0)


  stats <- filtered_data %>%

    summarise(

      Variable = var,

      Mean = mean(.data[[var]], na.rm = TRUE),

      SD = sd(.data[[var]], na.rm = TRUE),

      Min = min(.data[[var]], na.rm = TRUE),

      Max = max(.data[[var]], na.rm = TRUE),

      Median = median(.data[[var]], na.rm = TRUE),

      Q1 = quantile(.data[[var]], 0.25, na.rm = TRUE),

      Q3 = quantile(.data[[var]], 0.75, na.rm = TRUE)

    )

  summary_stats <- bind_rows(summary_stats, stats)

}

print(summary_stats)

write.xlsx(summary_stats, file =
"Filtered_IV_Fluids_Summary_Statistics.xlsx", row.names = FALSE)

cat("Summary statistics exported to
'Filtered_IV_Fluids_Summary_Statistics.xlsx'\n")
```

Glycocheck-TM Variables data distribution (normality)

```
> library(ggplot2)

>

> variables <- c("VVD", "RBC_filling", "PBR525", "PBR59", "PBR1019",
"PBR2025", "Median_P50")

>

> for (var in variables) {

+   shapiro_test <-
shapiro.test(Glycoheck_clinical_study_dataset_for_stats[[var]])

+   cat("Shapiro-Wilk test for variable", var, ":\n\n")

+   print(shapiro_test)

+   cat("\n\n")

+ }

Shapiro-Wilk test for variable VVD :

      Shapiro-Wilk normality test

data:  Glycoheck_clinical_study_dataset_for_stats[[var]]

W = 0.94935, p-value = 0.3852


Shapiro-Wilk test for variable RBC_filling :

      Shapiro-Wilk normality test

data:  Glycoheck_clinical_study_dataset_for_stats[[var]]

W = 0.97286, p-value = 0.8318


Shapiro-Wilk test for variable PBR525 :

      Shapiro-Wilk normality test

data:  Glycoheck_clinical_study_dataset_for_stats[[var]]

W = 0.93572, p-value = 0.2206


Shapiro-Wilk test for variable PBR59 :

      Shapiro-Wilk normality test

data:  Glycoheck_clinical_study_dataset_for_stats[[var]]

W = 0.91309, p-value = 0.08437


Shapiro-Wilk test for variable PBR1019 :

      Shapiro-Wilk normality test

data:  Glycoheck_clinical_study_dataset_for_stats[[var]]

W = 0.96107, p-value = 0.5936
```

```
Shapiro-Wilk test for variable PBR2025 :

	Shapiro-Wilk normality test

data:  Glycoheck_clinical_study_dataset_for_stats[[var]]
W = 0.96418, p-value = 0.657


Shapiro-Wilk test for variable Median_P50 :

	Shapiro-Wilk normality test

data:  Glycoheck_clinical_study_dataset_for_stats[[var]]
W = 0.94466, p-value = 0.3193
 ggplot(Glycoheck_clinical_study_dataset_for_stats, aes(x = VVD)) +
+   geom_histogram(binwidth = 0.1, fill = "blue", color = "black", alpha =
0.7) +
+   labs(title = "Histogram of VVD", x = "VVD", y = "Frequency") +
+   theme_minimal() +
+   theme(plot.title = element_text(hjust = 0.5))
Warning message:
Removed 1 row containing non-finite outside the scale range
(`stat_bin()`).
 ggplot(Glycoheck_clinical_study_dataset_for_stats, aes(x = RBC_filling))
+
+   geom_histogram(binwidth = 0.1, fill = "green", color = "black", alpha
= 0.7) +
+   labs(title = "Histogram of RBC_filling", x = "RBC_filling", y =
"Frequency") +
+   theme_minimal() +
+   theme(plot.title = element_text(hjust = 0.5))
Warning message:
Removed 1 row containing non-finite outside the scale range
(`stat_bin()`).


> ggplot(Glycoheck_clinical_study_dataset_for_stats, aes(x = PBR525)) +
+   geom_histogram(binwidth = 0.1, fill = "red", color = "black", alpha =
0.7) +
+   labs(title = "Histogram of PBR525", x = "PBR525", y = "Frequency") +
+   theme_minimal() +
+   theme(plot.title = element_text(hjust = 0.5))
Warning message:
Removed 1 row containing non-finite outside the scale range
(`stat_bin()`).
> ggplot(Glycoheck_clinical_study_dataset_for_stats, aes(x = PBR59)) +
```

```
+   geom_histogram(binwidth = 0.1, fill = "purple", color = "black", alpha
= 0.7) +
+   labs(title = "Histogram of PBR59", x = "PBR59", y = "Frequency") +
+   theme_minimal() +
+   theme(plot.title = element_text(hjust = 0.5))
Warning message:
Removed 1 row containing non-finite outside the scale range
(`stat_bin()`).


> ggplot(Glycoheck_clinical_study_dataset_for_stats, aes(x = PBR1019)) +
+   geom_histogram(binwidth = 0.1, fill = "orange", color = "black", alpha
= 0.7) +
+   labs(title = "Histogram of PBR1019", x = "PBR1019", y = "Frequency") +
+   theme_minimal() +
+   theme(plot.title = element_text(hjust = 0.5))
Warning message:
Removed 1 row containing non-finite outside the scale range
(`stat_bin()`).
 ggplot(Glycoheck_clinical_study_dataset_for_stats, aes(x = PBR2025)) +
+   geom_histogram(binwidth = 0.1, fill = "cyan", color = "black", alpha =
0.7) +
+   labs(title = "Histogram of PBR2025", x = "PBR2025", y = "Frequency") +
+   theme_minimal() +
+   theme(plot.title = element_text(hjust = 0.5))
Warning message:
Removed 1 row containing non-finite outside the scale range
(`stat_bin()`).
 ggplot(Glycoheck_clinical_study_dataset_for_stats, aes(x = Median_P50)) +
+   geom_histogram(binwidth = 0.1, fill = "pink", color = "black", alpha =
0.7) +
+   labs(title = "Histogram of Median_P50", x = "Median_P50", y =
"Frequency") +
+   theme_minimal() +
+   theme(plot.title = element_text(hjust = 0.5))
Warning message:
Removed 1 row containing non-finite outside the scale range
(`stat_bin()`).
```

GlycoCheck-TM variables summary statistics

(Results in Excel table)

```
summary_fun <- function(x) {
stats <- cbind(
mean = mean(x),
sd = sd(x),
median = median(x),
 min = min(x),
 max = max(x),
Q1 = quantile(x, probs = 0.25),
Q3 = quantile(x, probs = 0.75)
 )
return(stats)
}
var_to_analyse<-c("VVD", "RBC_filling", "PBR525", "PBR59", "PBR1019",
"PBR2025", "Median_P50")
descriptive_stats <- Glycoheck_clinical_study_dataset_for_stats %>%
summarize(across(all_of(var_to_analyse), summary_fun))

View(descriptive_stats)

library(tidyr)

tidy_descriptive_stats <- descriptive_stats %>%
pivot_longer(cols = everything(),
names_to = "Variable",
values_to = "Statistics")
var_to_analyse_patient<-c("Age_years", "Weight", "LOH",
"APPLE_fast",,"Total_ATT", "Short_ATT", "IV_fluid_challenge",
"IV_fluids_rate",
"IV_fluids_duration","IV_fluids_total")
descriptive_stats <- Glycoheck_clinical_study_dataset_for_stats %>%
summarize(across(all_of(var_to_analyse_patient), summary_fun))
View(descriptive_stats_patient)
tidy_descriptive_stats_patient <- descriptive_stats_patient %>%
pivot_longer(cols = everything(),
names_to = "Variable",
```

```
values_to = "Statistics")
View(tidy_descriptive_stats_patient)

#Descriptive per group#


data<-Glycocheck_data_clinical_average_tidy
summary_fun <- function(x) {
stats <- cbind(
mean = mean(x),
sd = sd(x),
median = median(x),
 min = min(x),
max = max(x),
Q1 = quantile(x, probs = 0.25),
Q3 = quantile(x, probs = 0.75)
 )
return(stats)
}
var_to_analyse<-c("VVD", "RBC_filling", "PBR525", "PBR59", "PBR1019",
"PBR2025", "Median_P50")



descriptive_stats <- Glycoheck_clinical_study_dataset_for_stats %>%
  +     group_by(Group) %>%
  +     summarize(across(all_of(vars_to_analyse), summary_fun))
View(descriptive_stats)



library(tidyr)


tidy_descriptive_stats <- descriptive_stats %>%
  pivot_longer(cols = -Group,
               names_to = "Variable",
               values_to = "Statistics")

print(tidy_descriptive_stats)
```

Confidence intervals Glycocheck-TM parameters

```
> t.test(Glycoheck_clinical_study_dataset_for_stats$PBR525)
      One Sample t-test
data:  Glycoheck_clinical_study_dataset_for_stats$PBR525
t = 51.57, df = 18, p-value < 2.2e-16
alternative hypothesis: true mean is not equal to 0
95 percent confidence interval:
 2.496771 2.708843
sample estimates:
mean of x
 2.602807

> clinidata<-Glycoheck_clinical_study_dataset_for_stats
> t.test(clinidata$RBC_filling)
      One Sample t-test
data:  clinidata$RBC_filling
t = 32.239, df = 18, p-value < 2.2e-16
alternative hypothesis: true mean is not equal to 0
95 percent confidence interval:
 0.5476972 0.6240572
sample estimates:
mean of x
0.5858772

> t.test(clinidata$VVD)
      One Sample t-test
data:  clinidata$VVD
t = 14.989, df = 18, p-value = 1.305e-11
alternative hypothesis: true mean is not equal to 0
95 percent confidence interval:
 331.6850 439.8237
sample estimates:
mean of x
 385.7544

> t.test(clinidata$PBR59)
      One Sample t-test
```

```
data:  clinidata$PBR59
t = 40.108, df = 18, p-value < 2.2e-16
alternative hypothesis: true mean is not equal to 0
95 percent confidence interval:
 1.306218 1.450624
sample estimates:
mean of x
 1.378421

> t.test(clinidata$PBR1019)
      One Sample t-test
data:  clinidata$PBR1019
t = 59.179, df = 18, p-value < 2.2e-16
alternative hypothesis: true mean is not equal to 0
95 percent confidence interval:
 2.909571 3.123762
sample estimates:
mean of x
 3.016667

> t.test(clinidata$PBR2025)
      One Sample t-test
data:  clinidata$PBR2025
t = 35.01, df = 18, p-value < 2.2e-16
alternative hypothesis: true mean is not equal to 0
95 percent confidence interval:
 2.851802 3.215918
sample estimates:
mean of x
  3.03386

> t.test(clinidata$Median_P50)
      One Sample t-test
data:  clinidata$Median_P50
t = 31.986, df = 18, p-value < 2.2e-16
alternative hypothesis: true mean is not equal to 0
95 percent confidence interval:
```

```
 7.713032 8.797494
sample estimates:
mean of x
 8.255263
```

Vascular segments analysis

Data distribution (Normality)

```
> for (variable in variables) {
+   shapiro_test <- 
shapiro.test(Glycoheck_clinical_study_dataset_for_stats[[variable]])
+   cat("Shapiro-Wilk test for", variable, ":\n")
+   cat("Test Statistic =", shapiro_test$statistic, ", p-value =", 
shapiro_test$p.value, "\n\n")
+   bin_size <- 20
+   hist(Glycoheck_clinical_study_dataset_for_stats[[variable]],
+        breaks = bin_size,
+        main = paste("Distribution of", variable),
+        xlab = variable,
+        ylab = "Frequency",
+        col = "lightblue",
+        border = "black")}
Shapiro-Wilk test for Count_59 :
Test Statistic = 0.957989 , p-value = 0.5334377

Shapiro-Wilk test for Count_1019 :
Test Statistic = 0.9212702 , p-value = 0.119458

Shapiro-Wilk test for Count_2025 :
Test Statistic = 0.9149542 , p-value = 0.09131407

Shapiro-Wilk test for Count_525 :
Test Statistic = 0.9860171 , p-value = 0.989094

> hist(Glycoheck_clinical_study_dataset_for_stats$Count_59,
+      breaks = 5, # Specify the number of breaks directly
+      main = "Distribution of Count_59",
+      xlab = "Count_59",
+      ylab = "Frequency",
+      col = "lightblue",
+      border = "black",
```

```
+       prob = TRUE) # Set to TRUE to show density instead of frequency

> lines(density(Glycoheck_clinical_study_dataset_for_stats$Count_59, na.rm
= TRUE),

+        col = "red", lwd = 2) # Add a density line

> hist(Glycoheck_clinical_study_dataset_for_stats$Count_1019,

+       breaks = 5, # Specify the number of breaks directly

+       main = "Distribution of Count_1019",

+       xlab = "Count_1019",

+       ylab = "Frequency",

+       col = "lightblue",

+       border = "black",

+       prob = TRUE) # Set to TRUE to show density instead of frequency

> lines(density(Glycoheck_clinical_study_dataset_for_stats$Count_1019,
na.rm = TRUE),

+        col = "red", lwd = 2) # Add a density line

> hist(Glycoheck_clinical_study_dataset_for_stats$Count_2025,

+       breaks = 5, # Specify the number of breaks directly

+       main = "Distribution of Count_2025",

+       xlab = "Count_2025",

+       ylab = "Frequency",

+       col = "lightblue",

+       border = "black",

+       prob = TRUE) # Set to TRUE to show density instead of frequency

> lines(density(Glycoheck_clinical_study_dataset_for_stats$Count_2025,
na.rm = TRUE),

+        col = "red", lwd = 2) # Add a density line

>  hist(Glycoheck_clinical_study_dataset_for_stats$Count_525,

+       breaks = 5, # Specify the number of breaks directly

+       main = "Distribution of Count_525",

+       xlab = "Count_525",

+       ylab = "Frequency",

+       col = "lightblue",

+       border = "black",

+       prob = TRUE) # Set to TRUE to show density instead of frequency

> lines(density(Glycoheck_clinical_study_dataset_for_stats$Count_525,
na.rm = TRUE),

+        col = "red", lwd = 2) # Add a density line
```

Vascular segment count summary statistics

```
>
> library(openxlsx)
Warning message:
package ‘openxlsx’ was built under R version 4.3.3
>
> summary_stats <- data.frame(
+   Variable = c("Count_59", "Count_1019", "Count_2025", "Count_525"),
+   Mean = c(mean(Glycoheck_clinical_study_dataset_for_stats$Count_59,
na.rm = TRUE),
+            mean(Glycoheck_clinical_study_dataset_for_stats$Count_1019,
na.rm = TRUE),
+            mean(Glycoheck_clinical_study_dataset_for_stats$Count_2025,
na.rm = TRUE),
+            mean(Glycoheck_clinical_study_dataset_for_stats$Count_525,
na.rm = TRUE)),
+   Median = c(median(Glycoheck_clinical_study_dataset_for_stats$Count_59,
na.rm = TRUE),
+
median(Glycoheck_clinical_study_dataset_for_stats$Count_1019, na.rm =
TRUE),
+
median(Glycoheck_clinical_study_dataset_for_stats$Count_2025, na.rm =
TRUE),
+
median(Glycoheck_clinical_study_dataset_for_stats$Count_525, na.rm =
TRUE)),
+   SD = c(sd(Glycoheck_clinical_study_dataset_for_stats$Count_59, na.rm =
TRUE),
+          sd(Glycoheck_clinical_study_dataset_for_stats$Count_1019, na.rm
= TRUE),
+          sd(Glycoheck_clinical_study_dataset_for_stats$Count_2025, na.rm
= TRUE),
+          sd(Glycoheck_clinical_study_dataset_for_stats$Count_525, na.rm
= TRUE)),
+   IQR = c(IQR(Glycoheck_clinical_study_dataset_for_stats$Count_59, na.rm
= TRUE),
+           IQR(Glycoheck_clinical_study_dataset_for_stats$Count_1019,
na.rm = TRUE),
+           IQR(Glycoheck_clinical_study_dataset_for_stats$Count_2025,
na.rm = TRUE),
+           IQR(Glycoheck_clinical_study_dataset_for_stats$Count_525,
na.rm = TRUE))
+ )
>
```

```
> write.xlsx(summary_stats, file = "Summary_Statistics.xlsx")
>
> print(summary_stats)
    Variable       Mean Median        SD   IQR
1   Count_59  994.42105    910 350.76224 515.0
2 Count_1019  505.05263    505 171.65096 285.5
3 Count_2025   29.94737     28  19.38348  21.0
4  Count_525 1529.42105   1513 356.04218 471.0
```

Comparison between segment groups and subgroups

```
> friedman_data <- data.frame(
+   Count_59 = Glycoheck_clinical_study_dataset_for_stats$Count_59,
+   Count_1019 = Glycoheck_clinical_study_dataset_for_stats$Count_1019,
+   Count_2025 = Glycoheck_clinical_study_dataset_for_stats$Count_2025,
+   Count_525 = Glycoheck_clinical_study_dataset_for_stats$Count_525
+ )
>
> friedman_data_long <- data.frame(
+   Value = c(
+     friedman_data$Count_59,
+     friedman_data$Count_1019,
+     friedman_data$Count_2025,
+     friedman_data$Count_525
+   ),
+   Group = factor(rep(c("Count_59", "Count_1019", "Count_2025",
"Count_525"),
+                      each = nrow(friedman_data))),
+   Subject = factor(rep(1:nrow(friedman_data), times = 4))
+ )
> friedman_result <- friedman.test(Value ~ Group | Subject, data =
friedman_data_long)
> print(friedman_result)


      Friedman rank sum test


data:  Value and Group and Subject
Friedman chi-squared = 54.853, df = 3, p-value = 7.382e-12
```

```
> pairwise_results <- pairwise.wilcox.test(friedman_data_long$Value,
friedman_data_long$Group,
+                                            paired = TRUE, p.adjust.method
= "bonferroni")
> print(pairwise_results)
```

```
Pairwise comparisons using Wilcoxon signed rank exact test

data:  friedman_data_long$Value and friedman_data_long$Group

           Count_1019 Count_2025 Count_525
Count_2025 2.3e-05    -          -
Count_525  2.3e-05    2.3e-05    -
Count_59   0.00023    2.3e-05    2.3e-05

P value adjustment method: bonferroni
```

```
> hist(residuals_PBR2025,
+      main = "Histogram of Residuals with Density Line for PBR2025",
+      xlab = "Residuals",
+      ylab = "Frequency",
+      col = "lightblue",
+      border = "black",
+      prob = TRUE)
> lines(density(residuals_PBR2025, na.rm = TRUE), col = "red", lwd = 2)
```

Vascular segment distribution per diameter from 5 to 25 bar plot

```
library(ggplot2)
ggplot(data = CS_vascular_segment_counts, aes(x = Diameter, y =
N_segments)) +
  geom_bar(stat = "identity", fill = "navy", width = 0.6) +
  labs(
    title = "Vascular segment distribution",
    x = "Major vascular segments with diameters from 5 to 25 µm",
    y = "Vascular segment counts"
```

```
  ) +
  scale_x_continuous(
    breaks = 5:25,
    labels = 5:25
  ) +
  geom_vline(xintercept = c(9.5, 19.5), linetype = "dashed", color =
"grey40") +
  theme_minimal() +
  theme(
    panel.grid = element_blank(),                    # Remove all grid
lines
    axis.line = element_line(color = "black"),      # Add axis lines
    axis.text.x = element_text(size = 10),
    axis.text.y = element_text(size = 10),
    axis.title = element_text(size = 12, face = "bold"),
    plot.title = element_text(size = 14, face = "bold", hjust = 0.5)
  )
```

Packed cell volume

PCV data distribution (normality)

```
> data <- Glycoheck_clinical_study_dataset_for_stats
>
> shapiro_test <- shapiro.test(data$PCV)
> print(shapiro_test)

      Shapiro-Wilk normality test

data:  data$PCV
W = 0.95942, p-value = 0.5905
```

PCV summary statistics

```
> summary_stats <- data %>%
+   summarise(
+     Mean = mean(PCV, na.rm = TRUE),
+     SD = sd(PCV, na.rm = TRUE),
+     Min = min(PCV, na.rm = TRUE),
+     Max = max(PCV, na.rm = TRUE),
+     Median = median(PCV, na.rm = TRUE),
+     Q1 = quantile(PCV, 0.25, na.rm = TRUE),
+     Q3 = quantile(PCV, 0.75, na.rm = TRUE)
+   )
>
> print(summary_stats)
# A tibble: 1 × 7
   Mean    SD   Min   Max Median    Q1    Q3
  <dbl> <dbl> <dbl> <dbl>  <dbl> <dbl> <dbl>
1  32.8  10.1    13    48     35    25    39
>
> ggplot(data, aes(x = PCV)) +
+   geom_histogram(aes(y = ..density..), binwidth = 2, fill = "blue",
color = "black", alpha = 0.7) +
+   geom_density(color = "red", size = 1) +
+   labs(title = "Distribution of PCV", x = "PCV", y = "Density") +
```

```
+   theme_minimal() +
+   theme(plot.title = element_text(hjust = 0.5))
Warning messages:
1: Removed 1 row containing non-finite outside the scale range
(`stat_bin()`).
2: Removed 1 row containing non-finite outside the scale range
(`stat_density()`).
```

Glycocheck-TM vs Clinical variables
Generalized linear models

VVD

```
> glm_VVD <- glm(VVD ~ Group + APPLE_fast + LOH,
+                 data = Glycoheck_clinical_study_dataset_for_stats,
+                 family = Gamma(link = "log"))
> summary(glm_VVD)

Call:
glm(formula = VVD ~ Group + APPLE_fast + LOH, family = Gamma(link =
"log"),
    data = Glycoheck_clinical_study_dataset_for_stats)

Coefficients:
            Estimate Std. Error t value Pr(>|t|)
(Intercept)  5.89568    0.28397  20.762 1.83e-12 ***
GroupT      -0.25724    0.13497  -1.906   0.0760 .
APPLE_fast   0.02089    0.01532   1.364   0.1928
LOH         -0.04092    0.02254  -1.815   0.0895 .
---
Signif. codes:  0 ‘***’ 0.001 ‘**’ 0.01 ‘*’ 0.05 ‘.’ 0.1 ‘ ’ 1

(Dispersion parameter for Gamma family taken to be 0.0757046)

    Null deviance: 1.6208  on 18  degrees of freedom
Residual deviance: 1.1832  on 15  degrees of freedom
AIC: 236.05

Number of Fisher Scoring iterations: 6

>
> residuals_VVD <- residuals(glm_VVD, type = "pearson")
>
> hist(residuals_VVD,
+      main = "Histogram of Residuals for VVD",
+      xlab = "Residuals",
+      ylab = "Frequency",
+      col = "lightblue",
+      border = "black",
+      prob = TRUE)
> curve(dnorm(x, mean = mean(residuals_VVD, na.rm = TRUE),
+             sd = sd(residuals_VVD, na.rm = TRUE)),
+       col = "blue", lwd = 2, add = TRUE)

#CI (Exp Est)
               Estimate       2.5 %
(Intercept) 363.4625984 203.6256500
GroupT        0.7731851   0.5896762
APPLE_fast    1.0211142   0.9876256
LOH           0.9599069   0.9168381
                97.5 %
(Intercept) 656.149667
GroupT        1.008563
APPLE_fast    1.055806
LOH           1.004616
```

RBC_filling

```
> glm_RBC_filling <- glm(RBC_filling ~ Group + APPLE_fast + LOH,
+                         data =
Glycoheck_clinical_study_dataset_for_stats,
+                         family = Gamma(link = "log"))
> summary(glm_RBC_filling)

Call:
```

```
glm(formula = RBC_filling ~ Group + APPLE_fast + LOH, family = Gamma(link
= "log"),
    data = Glycoheck_clinical_study_dataset_for_stats)

Coefficients:
             Estimate Std. Error t value Pr(>|t|)
(Intercept) -0.523342   0.152263  -3.437  0.00367 **
GroupT      -0.019639   0.072373  -0.271  0.78981
APPLE_fast   0.001664   0.008216   0.203  0.84222
LOH         -0.007324   0.012086  -0.606  0.55358
---
Signif. codes:  0 ‘***’ 0.001 ‘**’ 0.01 ‘*’ 0.05 ‘.’ 0.1 ‘ ’ 1

(Dispersion parameter for Gamma family taken to be 0.02176571)

    Null deviance: 0.33552  on 18  degrees of freedom
Residual deviance: 0.32725  on 15  degrees of freedom
AIC: -33.846

Number of Fisher Scoring iterations: 5

>
> residuals_RBC_filling <- residuals(glm_RBC_filling, type = "pearson")
>
> hist(residuals_RBC_filling,
+      main = "Histogram of Residuals for RBC_filling",
+      xlab = "Residuals",
+      ylab = "Frequency",
+      col = "lightblue",
+      border = "black",
+      prob = TRUE)
> curve(dnorm(x, mean = mean(residuals_RBC_filling, na.rm = TRUE),
+              sd = sd(residuals_RBC_filling, na.rm = TRUE)),
+       col = "blue", lwd = 2, add = TRUE)
>
> exp(cbind(
+   Estimate = coef(glm_RBC_filling),
+   confint(glm_RBC_filling)
+ ))
Estimate     2.5 %    97.5 %
(Intercept) 0.5925372 0.4352328 0.8091344
GroupT      0.9805523 0.8508274 1.1285740
APPLE_fast  1.0016653 0.9849083 1.0187305
LOH         0.9927028 0.9692344 1.0166863
```

Median_P50

```
> glm_Median_P50 <- glm(Median_P50 ~ Group + APPLE_fast + LOH,
+                       data = Glycoheck_clinical_study_dataset_for_stats,
+                       family = Gamma(link = "log"))
> summary(glm_Median_P50)

Call:
glm(formula = Median_P50 ~ Group + APPLE_fast + LOH, family = Gamma(link =
"log"),
    data = Glycoheck_clinical_study_dataset_for_stats)

Coefficients:
              Estimate Std. Error t value Pr(>|t|)
(Intercept)  2.1377140  0.1528324  13.987 5.19e-10 ***
GroupT       0.0244554  0.0726436   0.337    0.741
APPLE_fast  -0.0025952  0.0082463  -0.315    0.757
LOH          0.0008008  0.0121310   0.066    0.948
---
Signif. codes:  0 ‘***’ 0.001 ‘**’ 0.01 ‘*’ 0.05 ‘.’ 0.1 ‘ ’ 1

(Dispersion parameter for Gamma family taken to be 0.02192885)
```

```
    Null deviance: 0.33378  on 18  degrees of freedom
Residual deviance: 0.32940  on 15  degrees of freedom
AIC: 66.81

Number of Fisher Scoring iterations: 4

>
> residuals_Median_P50 <- residuals(glm_Median_P50, type = "pearson")
>
> hist(residuals_Median_P50,
+      main = "Histogram of Residuals for Median_P50",
+      xlab = "Residuals",
+      ylab = "Frequency",
+      col = "lightblue",
+      border = "black",
+      prob = TRUE)
> curve(dnorm(x, mean = mean(residuals_Median_P50, na.rm = TRUE),
+             sd = sd(residuals_Median_P50, na.rm = TRUE)),
+       col = "blue", lwd = 2, add = TRUE)


> exp(cbind(
+   Estimate = coef(glm_Median_P50),
+   confint.default(glm_Median_P50)
+ ))
             Estimate     2.5 %    97.5 %
(Intercept) 8.4800301 6.2850129 11.441649
GroupT      1.0247569 0.8887641  1.181558
APPLE_fast  0.9974081 0.9814171  1.013660
LOH         1.0008011 0.9772864  1.024882
```

PBR525

```
> glm_PBR525 <- glm(PBR525 ~ Group + APPLE_fast + LOH,
+                   data = Glycoheck_clinical_study_dataset_for_stats,
+                   family = Gamma(link = "log"))
> summary(glm_PBR525)

Call:
glm(formula = PBR525 ~ Group + APPLE_fast + LOH, family = Gamma(link =
"log"),
    data = Glycoheck_clinical_study_dataset_for_stats)

Coefficients:
            Estimate Std. Error t value Pr(>|t|)
(Intercept) 0.897154   0.094021   9.542 9.22e-08 ***
GroupT      0.001523   0.044689   0.034    0.973
APPLE_fast  0.003157   0.005073   0.622    0.543
LOH         0.001185   0.007463   0.159    0.876
---
Signif. codes:  0 ‘***’ 0.001 ‘**’ 0.01 ‘*’ 0.05 ‘.’ 0.1 ‘ ’ 1

(Dispersion parameter for Gamma family taken to be 0.008299109)

    Null deviance: 0.12737  on 18  degrees of freedom
Residual deviance: 0.12346  on 15  degrees of freedom
AIC: 4.4733

Number of Fisher Scoring iterations: 3

>
> residuals_PBR525 <- residuals(glm_PBR525, type = "pearson")
>
> hist(residuals_PBR525,
+      main = "Histogram of Residuals for PBR525",
+      xlab = "Residuals",
+      ylab = "Frequency",
+      col = "lightblue",
```

```
+      border = "black",
+      prob = TRUE)
> curve(dnorm(x, mean = mean(residuals_PBR525, na.rm = TRUE),
+              sd = sd(residuals_PBR525, na.rm = TRUE)),
+       col = "blue", lwd = 2, add = TRUE)


> exp(cbind(
+   Estimate = coef(glm_PBR525),
+   confint.default(glm_PBR525)
+ ))
            Estimate     2.5 %   97.5 %
(Intercept) 2.452614 2.0398521 2.948898
GroupT      1.001524 0.9175332 1.093204
APPLE_fast  1.003162 0.9932370 1.013186
LOH         1.001186 0.9866482 1.015938
```

PBR59

```
> glm_PBR59 <- glm(PBR59 ~ Group + APPLE_fast + LOH,
+                  data = Glycoheck_clinical_study_dataset_for_stats,
+                  family = Gamma(link = "log"))
> summary(glm_PBR59)

Call:
glm(formula = PBR59 ~ Group + APPLE_fast + LOH, family = Gamma(link =
"log"),
    data = Glycoheck_clinical_study_dataset_for_stats)

Coefficients:
              Estimate Std. Error t value Pr(>|t|)
(Intercept)  0.3773970  0.1195573   3.157  0.00652 **
GroupT      -0.0645504  0.0568274  -1.136  0.27382
APPLE_fast  -0.0006166  0.0064509  -0.096  0.92512
LOH         -0.0023202  0.0094898  -0.244  0.81016
---
Signif. codes:  0 ‘***’ 0.001 ‘**’ 0.01 ‘*’ 0.05 ‘.’ 0.1 ‘ ’ 1

(Dispersion parameter for Gamma family taken to be 0.01341952)

    Null deviance: 0.23410  on 18  degrees of freedom
Residual deviance: 0.21647  on 15  degrees of freedom
AIC: -9.1025

Number of Fisher Scoring iterations: 4

>
> residuals_PBR59 <- residuals(glm_PBR59, type = "pearson")
>
> hist(residuals_PBR59,
+      main = "Histogram of Residuals for PBR59",
+      xlab = "Residuals",
+      ylab = "Frequency",
+      col = "lightblue",
+      border = "black",
+      prob = TRUE)
> curve(dnorm(x, mean = mean(residuals_PBR59, na.rm = TRUE),
+              sd = sd(residuals_PBR59, na.rm = TRUE)),
+       col = "blue", lwd = 2, add = TRUE)
>

> exp(cbind(
+   Estimate = coef(glm_PBR59),
+   confint.default(glm_PBR59)
+ ))
             Estimate     2.5 %   97.5 %
(Intercept) 1.4584832 1.1538094 1.843609
GroupT      0.9374889 0.8386766 1.047943
```

```
APPLE_fast  0.9993836 0.9868274 1.012100
LOH         0.9976825 0.9792974 1.016413
```

PBR1019

```
> glm_PBR1019 <- glm(PBR1019 ~ Group + APPLE_fast + LOH,
+                     data = Glycoheck_clinical_study_dataset_for_stats,
+                     family = Gamma(link = "log"))
> summary(glm_PBR1019)

Call:
glm(formula = PBR1019 ~ Group + APPLE_fast + LOH, family = Gamma(link = 
"log"), 
    data = Glycoheck_clinical_study_dataset_for_stats)

Coefficients:
             Estimate Std. Error t value Pr(>|t|)    
(Intercept) 1.0702963  0.0821361  13.031 1.39e-09 ***
GroupT      0.0111420  0.0390405   0.285    0.779    
APPLE_fast  0.0008394  0.0044318   0.189    0.852    
LOH         0.0033455  0.0065195   0.513    0.615    
---
Signif. codes:  0 ‘***’ 0.001 ‘**’ 0.01 ‘*’ 0.05 ‘.’ 0.1 ‘ ’ 1

(Dispersion parameter for Gamma family taken to be 0.00633363)

    Null deviance: 0.096152  on 18  degrees of freedom
Residual deviance: 0.093832  on 15  degrees of freedom
AIC: 4.894

Number of Fisher Scoring iterations: 4

> 
> residuals_PBR1019 <- residuals(glm_PBR1019, type = "pearson")
> 
> hist(residuals_PBR1019,
+      main = "Histogram of Residuals for PBR1019",
+      xlab = "Residuals",
+      ylab = "Frequency",
+      col = "lightblue",
+      border = "black",
+      prob = TRUE)
> curve(dnorm(x, mean = mean(residuals_PBR1019, na.rm = TRUE),
+              sd = sd(residuals_PBR1019, na.rm = TRUE)),
+       col = "blue", lwd = 2, add = TRUE)
```

PBR2025

```
> glm_PBR2025 <- glm(PBR2025 ~ Group + APPLE_fast + LOH,
+                     data = Glycoheck_clinical_study_dataset_for_stats,
+                     family = Gamma(link = "log"))
> summary(glm_PBR2025)

Call:
glm(formula = PBR2025 ~ Group + APPLE_fast + LOH, family = Gamma(link = 
"log"), 
    data = Glycoheck_clinical_study_dataset_for_stats)

Coefficients:
             Estimate Std. Error t value Pr(>|t|)    
(Intercept) 0.9539468  0.1350891   7.062 3.86e-06 ***
GroupT      0.0189200  0.0642099   0.295    0.772    
APPLE_fast  0.0082903  0.0072889   1.137    0.273    
LOH         0.0007412  0.0107226   0.069    0.946    
---
Signif. codes:  0 ‘***’ 0.001 ‘**’ 0.01 ‘*’ 0.05 ‘.’ 0.1 ‘ ’ 1
```

```
(Dispersion parameter for Gamma family taken to be 0.01713268)

    Null deviance: 0.28782  on 18  degrees of freedom
Residual deviance: 0.26206  on 15  degrees of freedom
AIC: 24.461

Number of Fisher Scoring iterations: 4

>
> residuals_PBR2025 <- residuals(glm_PBR2025, type = "pearson")
>
> hist(residuals_PBR2025,
+      main = "Histogram of Residuals for PBR2025",
+      xlab = "Residuals",
+      ylab = "Frequency",
+      col = "lightblue",
+      border = "black",
+      prob = TRUE)
> curve(dnorm(x, mean = mean(residuals_PBR2025, na.rm = TRUE),
+             sd = sd(residuals_PBR2025, na.rm = TRUE)),
+       col = "blue", lwd = 2, add = TRUE)

> exp(cbind(
+   Estimate = coef(glm_PBR2025),
+   confint.default(glm_PBR2025)
+ ))
            Estimate     2.5 %   97.5 %
(Intercept) 2.595935 1.9920755 3.382843
GroupT      1.019100 0.8985894 1.155773
APPLE_fast  1.008325 0.9940222 1.022833
LOH         1.000741 0.9799293 1.021996
```

Wald-based confidence intervals

VVD

```
> glm_VVD <- glm(VVD ~ Group + APPLE_fast + LOH,data =
Glycocheck_clinical_study_dataset_for_stats,family = Gamma(link = "log"))
> exp(cbind(
+   Estimate = coef(glm_VVD),
+   confint(glm_VVD)
+ ))
Waiting for profiling to be done...              Estimate       2.5 %
(Intercept) 363.4625984 203.6256500
GroupT        0.7731851   0.5896762
APPLE_fast    1.0211142   0.9876256
LOH           0.9599069   0.9168381
                97.5 %
(Intercept) 656.149667
GroupT        1.008563
APPLE_fast    1.055806
LOH           1.004616
```

%RBC filling

```
> glm_RBC_filling <- glm(RBC_filling ~ Group + APPLE_fast + LOH,data =
Glycocheck_clinical_study_dataset_for_stats,family = Gamma(link = "log"))
> exp(cbind(
+   Estimate = coef(glm_RBC_filling),
+   confint(glm_RBC_filling)
+ ))
Waiting for profiling to be done...             Estimate     2.5 %    97.5
%
(Intercept) 0.5925372 0.4352328 0.8091344
GroupT      0.9805523 0.8508274 1.1285740
APPLE_fast  1.0016653 0.9849083 1.0187305
LOH         0.9927028 0.9692344 1.0166863
> glm_Median_P50 <- glm(
+   Median_P50 ~ Group + APPLE_fast + LOH,
+   data = Glycocheck_clinical_study_dataset_for_stats,
+   family = Gamma(link = "log")
```

```
+ )
>
> exp(cbind(
+   Estimate = coef(glm_Median_P50),
+   confint.default(glm_Median_P50)
+ ))
             Estimate     2.5 %    97.5 %
(Intercept) 8.4800301 6.2850129 11.441649
GroupT      1.0247569 0.8887641  1.181558
APPLE_fast  0.9974081 0.9814171  1.013660
LOH         1.0008011 0.9772864  1.024882
```

PBR525

```
> glm_PBR525 <- glm(
+   PBR525 ~ Group + APPLE_fast + LOH,
+   data = Glycocheck_clinical_study_dataset_for_stats,
+   family = Gamma(link = "log")
+ )
>
> exp(cbind(
+   Estimate = coef(glm_PBR525),
+   confint.default(glm_PBR525)
+ ))
            Estimate     2.5 %   97.5 %
(Intercept) 2.452614 2.0398521 2.948898
GroupT      1.001524 0.9175332 1.093204
APPLE_fast  1.003162 0.9932370 1.013186
LOH         1.001186 0.9866482 1.015938
```

PBR59

```
> glm_PBR59 <- glm(
+   PBR59 ~ Group + APPLE_fast + LOH,
+   data = Glycocheck_clinical_study_dataset_for_stats,
+   family = Gamma(link = "log")
+ )
>
> exp(cbind(
+   Estimate = coef(glm_PBR59),
+   confint.default(glm_PBR59)
+ ))
             Estimate     2.5 %   97.5 %
(Intercept) 1.4584832 1.1538094 1.843609
GroupT      0.9374889 0.8386766 1.047943
APPLE_fast  0.9993836 0.9868274 1.012100
LOH         0.9976825 0.9792974 1.016413
```

PBR1019

```
> glm_PBR1019 <- glm(
+   PBR1019 ~ Group + APPLE_fast + LOH,
+   data = Glycocheck_clinical_study_dataset_for_stats,
+   family = Gamma(link = "log")
+ )
>
> exp(cbind(
+   Estimate = coef(glm_PBR1019),
+   confint.default(glm_PBR1019)
+ ))
            Estimate     2.5 %   97.5 %
(Intercept) 2.916243 2.4826152 3.425612
GroupT      1.011204 0.9367151 1.091617
APPLE_fast  1.000840 0.9921840 1.009571
LOH         1.003351 0.9906119 1.016254
```

Glycocheck-TM vs IV fluid variables – generalized linear models

```
> subset_data <- subset(Glycoheck_clinical_study_dataset_for_stats,
IV_fluids_Y_N == "Y")
>
```

VVD

```
> glm_VVD <- glm(VVD ~ IV_fluids_rate + IV_fluids_duration +
IV_fluids_total,
+                data = subset_data,
+                family = Gamma(link = "log"))
> summary(glm_VVD)

Call:
glm(formula = VVD ~ IV_fluids_rate + IV_fluids_duration + IV_fluids_total,
    family = Gamma(link = "log"), data = subset_data)

Coefficients:
                    Estimate Std. Error t value Pr(>|t|)
(Intercept)         4.804478   0.432182  11.117 3.16e-05 ***
IV_fluids_rate      0.071514   0.027046   2.644   0.0383 *
IV_fluids_duration  0.051562   0.024356   2.117   0.0786 .
IV_fluids_total    -0.003184   0.001613  -1.974   0.0958 .
---
Signif. codes:  0 '***' 0.001 '**' 0.01 '*' 0.05 '.' 0.1 ' ' 1

(Dispersion parameter for Gamma family taken to be 0.0955655)

    Null deviance: 1.28050  on 9  degrees of freedom
Residual deviance: 0.55961  on 6  degrees of freedom
AIC: 126.99

Number of Fisher Scoring iterations: 5

>
> residuals_VVD <- residuals(glm_VVD, type = "pearson")
>
> hist(residuals_VVD,
+      main = "Histogram of Residuals for VVD (Group Y)",
+      xlab = "Residuals",
+      ylab = "Frequency",
+      col = "lightblue",
+      border = "black",
+      prob = TRUE)
> curve(dnorm(x, mean = mean(residuals_VVD, na.rm = TRUE),
+              sd = sd(residuals_VVD, na.rm = TRUE)),
+       col = "blue", lwd = 2, add = TRUE)

                      Estimate      2.5 %
(Intercept)        122.0557430 52.3219452
IV_fluids_rate       1.0741327  1.0186765
IV_fluids_duration   1.0529142  1.0038331
IV_fluids_total      0.9968212  0.9936753
                        97.5 %
(Intercept)        284.7295593
IV_fluids_rate       1.1326079
IV_fluids_duration   1.1043951
IV_fluids_total      0.9999771


> confint(glm_VVD)
Waiting for profiling to be done...                         2.5 %
(Intercept)         3.913403929
IV_fluids_rate      0.019292015
IV_fluids_duration  0.003048313
IV_fluids_total    -0.006367730
                         97.5 %
(Intercept)         5.694266e+00
```

```
IV_fluids_rate      1.260561e-01
IV_fluids_duration  1.030987e-01
IV_fluids_total    -4.779418e-05
```

RBC_filling

```
> glm_RBC_filling <- glm(RBC_filling ~ IV_fluids_rate + IV_fluids_duration
+ IV_fluids_total,
+                        data = subset_data,
+                        family = Gamma(link = "log"))
> summary(glm_RBC_filling)

Call:
glm(formula = RBC_filling ~ IV_fluids_rate + IV_fluids_duration +
    IV_fluids_total, family = Gamma(link = "log"), data = subset_data)

Coefficients:
                     Estimate Std. Error t value Pr(>|t|)
(Intercept)        -0.8171511  0.2320649  -3.521   0.0125 *
IV_fluids_rate      0.0221445  0.0145227   1.525   0.1781
IV_fluids_duration  0.0099166  0.0130780   0.758   0.4770
IV_fluids_total    -0.0007713  0.0008660  -0.891   0.4074
---
Signif. codes:  0 ‘***’ 0.001 ‘**’ 0.01 ‘*’ 0.05 ‘.’ 0.1 ‘ ’ 1

(Dispersion parameter for Gamma family taken to be 0.02755406)

    Null deviance: 0.23969  on 9  degrees of freedom
Residual deviance: 0.15058  on 6  degrees of freedom
AIC: -14.603

Number of Fisher Scoring iterations: 4

>
> residuals_RBC_filling <- residuals(glm_RBC_filling, type = "pearson")
>
> hist(residuals_RBC_filling,
+      main = "Histogram of Residuals for RBC_filling (Group Y)",
+      xlab = "Residuals",
+      ylab = "Frequency",
+      col = "lightblue",
+      border = "black",
+      prob = TRUE)
> curve(dnorm(x, mean = mean(residuals_RBC_filling, na.rm = TRUE),
+             sd = sd(residuals_RBC_filling, na.rm = TRUE)),
+       col = "blue", lwd = 2, add = TRUE)

> exp(cbind(
+   Estimate = coef(glm_RBC_filling),
+   confint.default(glm_RBC_filling)
+ ))
                    Estimate     2.5 %
(Intercept)        0.4416882 0.2802734
IV_fluids_rate     1.0223915 0.9937005
IV_fluids_duration 1.0099660 0.9844071
IV_fluids_total    0.9992290 0.9975344
                      97.5 %
(Intercept)        0.6960649
IV_fluids_rate     1.0519109
IV_fluids_duration 1.0361884
IV_fluids_total    1.0009264

>
```

Median_P50

```
> glm_Median_P50 <- glm(Median_P50 ~ IV_fluids_rate + IV_fluids_duration +
IV_fluids_total,
+                       data = subset_data,
+                       family = Gamma(link = "log"))
```

```
> summary(glm_Median_P50)

Call:
glm(formula = Median_P50 ~ IV_fluids_rate + IV_fluids_duration + 
    IV_fluids_total, family = Gamma(link = "log"), data = subset_data)

Coefficients:
                    Estimate Std. Error t value Pr(>|t|)    
(Intercept)         2.435107   0.113352  21.483 6.64e-07 ***
IV_fluids_rate     -0.019890   0.007094  -2.804   0.0310 *  
IV_fluids_duration -0.020748   0.006388  -3.248   0.0175 *  
IV_fluids_total     0.001226   0.000423   2.897   0.0274 *  
---
Signif. codes:  0 ‘***’ 0.001 ‘**’ 0.01 ‘*’ 0.05 ‘.’ 0.1 ‘ ’ 1

(Dispersion parameter for Gamma family taken to be 0.006573887)

    Null deviance: 0.110072  on 9  degrees of freedom
Residual deviance: 0.040195  on 6  degrees of freedom
AIC: 24.926

Number of Fisher Scoring iterations: 3

> 
> residuals_Median_P50 <- residuals(glm_Median_P50, type = "pearson")
> 
> hist(residuals_Median_P50,
+      main = "Histogram of Residuals for Median_P50 (Group Y)",
+      xlab = "Residuals",
+      ylab = "Frequency",
+      col = "lightblue",
+      border = "black",
+      prob = TRUE)
> curve(dnorm(x, mean = mean(residuals_Median_P50, na.rm = TRUE),
+              sd = sd(residuals_Median_P50, na.rm = TRUE)),
+       col = "blue", lwd = 2, add = TRUE)
> 

> exp(cbind(
+   Estimate = coef(glm_Median_P50),
+   confint.default(glm_Median_P50)
+ ))
                     Estimate     2.5 %
(Intercept)        11.4170398 9.1425734
IV_fluids_rate      0.9803061 0.9667711
IV_fluids_duration  0.9794659 0.9672794
IV_fluids_total     1.0012263 1.0003965
                       97.5 %
(Intercept)        14.2573421
IV_fluids_rate      0.9940306
IV_fluids_duration  0.9918060
IV_fluids_total     1.0020567


> confint(glm_Median_P50)
Waiting for profiling to be done...                          2.5 %
(Intercept)         2.2129127406
IV_fluids_rate     -0.0338446531
IV_fluids_duration -0.0331266641
IV_fluids_total     0.0003955371
                          97.5 %
(Intercept)         2.657575617
IV_fluids_rate     -0.005777280
IV_fluids_duration -0.008204790
IV_fluids_total     0.002054768
```

PBR525

```
> glm_PBR525 <- glm(PBR525 ~ IV_fluids_rate + IV_fluids_duration +
IV_fluids_total,
+                    data = subset_data,
+                    family = Gamma(link = "log"))
> summary(glm_PBR525)

Call:
glm(formula = PBR525 ~ IV_fluids_rate + IV_fluids_duration +
    IV_fluids_total, family = Gamma(link = "log"), data = subset_data)

Coefficients:
                     Estimate Std. Error t value Pr(>|t|)
(Intercept)         1.0454014  0.1273782   8.207 0.000177 ***
IV_fluids_rate     -0.0089994  0.0079714  -1.129 0.302017
IV_fluids_duration -0.0031983  0.0071784  -0.446 0.671550
IV_fluids_total     0.0003238  0.0004753   0.681 0.521115
---
Signif. codes:  0 '***' 0.001 '**' 0.01 '*' 0.05 '.' 0.1 ' ' 1

(Dispersion parameter for Gamma family taken to be 0.008301516)

    Null deviance: 0.066967  on 9  degrees of freedom
Residual deviance: 0.049954  on 6  degrees of freedom
AIC: 4.3969

Number of Fisher Scoring iterations: 4

>
> residuals_PBR525 <- residuals(glm_PBR525, type = "pearson")
>
> hist(residuals_PBR525,
+      main = "Histogram of Residuals for PBR525 (Group Y)",
+      xlab = "Residuals",
+      ylab = "Frequency",
+      col = "lightblue",
+      border = "black",
+      prob = TRUE)
> curve(dnorm(x, mean = mean(residuals_PBR525, na.rm = TRUE),
+              sd = sd(residuals_PBR525, na.rm = TRUE)),
+       col = "blue", lwd = 2, add = TRUE)
>

> exp(cbind(
+   Estimate = coef(glm_PBR525),
+   confint.default(glm_PBR525)
+ ))
                    Estimate     2.5 %
(Intercept)        2.8445401 2.2160906
IV_fluids_rate     0.9910409 0.9756777
IV_fluids_duration 0.9968068 0.9828806
IV_fluids_total    1.0003239 0.9993924
                      97.5 %
(Intercept)        3.651208
IV_fluids_rate     1.006646
IV_fluids_duration 1.010930
IV_fluids_total    1.001256
```

PBR59

```
> glm_PBR59 <- glm(PBR59 ~ IV_fluids_rate + IV_fluids_duration +
IV_fluids_total,
+                   data = subset_data,
+                   family = Gamma(link = "log"))
> summary(glm_PBR59)

Call:
glm(formula = PBR59 ~ IV_fluids_rate + IV_fluids_duration +
IV_fluids_total,
```

```
    family = Gamma(link = "log"), data = subset_data)

Coefficients:
                     Estimate Std. Error t value Pr(>|t|)
(Intercept)         2.891e-01  2.048e-01   1.411    0.208
IV_fluids_rate     -1.157e-03  1.282e-02  -0.090    0.931
IV_fluids_duration  2.343e-03  1.154e-02   0.203    0.846
IV_fluids_total    -1.367e-05  7.643e-04  -0.018    0.986

(Dispersion parameter for Gamma family taken to be 0.02146437)

    Null deviance: 0.15329  on 9  degrees of freedom
Residual deviance: 0.14564  on 6  degrees of freedom
AIC: 2.2926

Number of Fisher Scoring iterations: 4

>
> residuals_PBR59 <- residuals(glm_PBR59, type = "pearson")
>
> hist(residuals_PBR59,
+      main = "Histogram of Residuals for PBR59 (Group Y)",
+      xlab = "Residuals",
+      ylab = "Frequency",
+      col = "lightblue",
+      border = "black",
+      prob = TRUE)
> curve(dnorm(x, mean = mean(residuals_PBR59, na.rm = TRUE),
+             sd = sd(residuals_PBR59, na.rm = TRUE)),
+       col = "blue", lwd = 2, add = TRUE)

> exp(cbind(
+   Estimate = coef(glm_PBR59),
+   confint.default(glm_PBR59)
+ ))
                    Estimate     2.5 %
(Intercept)        1.3351887 0.8937134
IV_fluids_rate     0.9988436 0.9740629
IV_fluids_duration 1.0023456 0.9799239
IV_fluids_total    0.9999863 0.9984894
                     97.5 %
(Intercept)        1.994743
IV_fluids_rate     1.024255
IV_fluids_duration 1.025280
IV_fluids_total    1.001485
```

PBR1019

```
> glm_PBR1019 <- glm(PBR1019 ~ IV_fluids_rate + IV_fluids_duration +
IV_fluids_total,
+                      data = subset_data,
+                      family = Gamma(link = "log"))
> summary(glm_PBR1019)

Call:
glm(formula = PBR1019 ~ IV_fluids_rate + IV_fluids_duration +
    IV_fluids_total, family = Gamma(link = "log"), data = subset_data)

Coefficients:
                     Estimate Std. Error t value Pr(>|t|)
(Intercept)         1.180e+00  1.116e-01  10.581 4.19e-05 ***
IV_fluids_rate     -6.571e-03  6.981e-03  -0.941    0.383
IV_fluids_duration -4.092e-04  6.287e-03  -0.065    0.950
IV_fluids_total     4.764e-05  4.163e-04   0.114    0.913
---
Signif. codes:  0 ‘***’ 0.001 ‘**’ 0.01 ‘*’ 0.05 ‘.’ 0.1 ‘ ’ 1

(Dispersion parameter for Gamma family taken to be 0.00636716)
```

```
    Null deviance: 0.055371  on 9  degrees of freedom
Residual deviance: 0.038076  on 6  degrees of freedom
AIC: 4.7736

Number of Fisher Scoring iterations: 4

>
> residuals_PBR1019 <- residuals(glm_PBR1019, type = "pearson")
>
> hist(residuals_PBR1019,
+      main = "Histogram of Residuals for PBR1019 (Group Y)",
+      xlab = "Residuals",
+      ylab = "Frequency",
+      col = "lightblue",
+      border = "black",
+      prob = TRUE)
> curve(dnorm(x, mean = mean(residuals_PBR1019, na.rm = TRUE),
+              sd = sd(residuals_PBR1019, na.rm = TRUE)),
+       col = "blue", lwd = 2, add = TRUE)

# PBR1019
glm_PBR1019 <- glm(
  PBR1019 ~ IV_fluids_rate + IV_fluids_duration + IV_fluids_total,
  data = subset_data,
  family = Gamma(link = "log")
)

exp(cbind(
  Estimate = coef(glm_PBR1019),
  confint.default(glm_PBR1019)
))
```

PBR2025

```
> glm_PBR2025 <- glm(PBR2025 ~ IV_fluids_rate + IV_fluids_duration +
IV_fluids_total,
+                    data = subset_data,
+                    family = Gamma(link = "log"))
> summary(glm_PBR2025)

Call:
glm(formula = PBR2025 ~ IV_fluids_rate + IV_fluids_duration +
    IV_fluids_total, family = Gamma(link = "log"), data = subset_data)

Coefficients:
                     Estimate Std. Error t value Pr(>|t|)
(Intercept)         1.2541281  0.1888149   6.642 0.000562 ***
IV_fluids_rate     -0.0172953  0.0118161  -1.464 0.193607
IV_fluids_duration -0.0084374  0.0106406  -0.793 0.458011
IV_fluids_total     0.0009359  0.0007046   1.328 0.232373
---
Signif. codes:  0 ‘***’ 0.001 ‘**’ 0.01 ‘*’ 0.05 ‘.’ 0.1 ‘ ’ 1

(Dispersion parameter for Gamma family taken to be 0.01824061)

    Null deviance: 0.17771  on 9  degrees of freedom
Residual deviance: 0.10785  on 6  degrees of freedom
AIC: 15.044

Number of Fisher Scoring iterations: 4

>
> residuals_PBR2025 <- residuals(glm_PBR2025, type = "pearson")
>
> hist(residuals_PBR2025,
+      main = "Histogram of Residuals for PBR2025 (Group Y)",
+      xlab = "Residuals",
+      ylab = "Frequency",
+      col = "lightblue",
```

```
+      border = "black",
+      prob = TRUE)
> curve(dnorm(x, mean = mean(residuals_PBR2025, na.rm = TRUE),
+              sd = sd(residuals_PBR2025, na.rm = TRUE)),
+       col = "blue", lwd = 2, add = TRUE)

> exp(cbind(
+   Estimate = coef(glm_PBR2025),
+   confint.default(glm_PBR2025)
+ ))
                    Estimate     2.5 %
(Intercept)        3.5047813 2.4207024
IV_fluids_rate     0.9828534 0.9603530
IV_fluids_duration 0.9915981 0.9711322
IV_fluids_total    1.0009363 0.9995550
                     97.5 %
(Intercept)        5.074350
IV_fluids_rate     1.005881
IV_fluids_duration 1.012495
IV_fluids_total    1.002320
```

GlycoCheck-TM Trauma subset

```
> subset_data_T <- subset(Glycoheck_clinical_study_dataset_for_stats,
Group == "T")
```

VVD

```
> glm_VVD <- glm(VVD ~ Total_ATT,
+               data = subset_data_T,
+               family = Gamma(link = "log"))
> summary(glm_VVD)

Call:
glm(formula = VVD ~ Total_ATT, family = Gamma(link = "log"),
    data = subset_data_T)

Coefficients:
            Estimate Std. Error t value Pr(>|t|)    
(Intercept)  6.00058    0.22627  26.519 7.45e-10 ***
Total_ATT   -0.03111    0.05216  -0.597    0.566    
---
Signif. codes:  0 ‘***’ 0.001 ‘**’ 0.01 ‘*’ 0.05 ‘.’ 0.1 ‘ ’ 1

(Dispersion parameter for Gamma family taken to be 0.0623289)

    Null deviance: 0.65028  on 10  degrees of freedom
Residual deviance: 0.62564  on  9  degrees of freedom
AIC: 134.37

Number of Fisher Scoring iterations: 5

> 
> residuals_VVD <- residuals(glm_VVD, type = "pearson")
> 
> hist(residuals_VVD,
+      main = "Histogram of Residuals for VVD (Group T)",
+      xlab = "Residuals",
+      ylab = "Frequency",
+      col = "lightblue",
+      border = "black",
+      prob = TRUE)
> curve(dnorm(x, mean = mean(residuals_VVD, na.rm = TRUE),
+              sd = sd(residuals_VVD, na.rm = TRUE)),
+       col = "blue", lwd = 2, add = TRUE)

> exp(cbind(
+   Estimate = coef(glm_VVD),
+   confint.default(glm_VVD)
```

```
+ ))
              Estimate       2.5 %
(Intercept) 403.6646461 259.0706237
Total_ATT     0.9693651   0.8751618
               97.5 %
(Intercept) 628.960336
Total_ATT     1.073708
```

RBC_filling

```
> glm_RBC_filling <- glm(RBC_filling ~ Total_ATT,
+                         data = subset_data_T,
+                         family = Gamma(link = "log"))
> summary(glm_RBC_filling)

Call:
glm(formula = RBC_filling ~ Total_ATT, family = Gamma(link = "log"),
    data = subset_data_T)

Coefficients:
            Estimate Std. Error t value Pr(>|t|)
(Intercept) -0.59168    0.12805  -4.621  0.00125 **
Total_ATT    0.01322    0.02952   0.448  0.66492
---
Signif. codes:  0 ‘***’ 0.001 ‘**’ 0.01 ‘*’ 0.05 ‘.’ 0.1 ‘ ’ 1

(Dispersion parameter for Gamma family taken to be 0.01996133)

    Null deviance: 0.17699  on 10  degrees of freedom
Residual deviance: 0.17322  on  9  degrees of freedom
AIC: -20.417

Number of Fisher Scoring iterations: 4

>
> residuals_RBC_filling <- residuals(glm_RBC_filling, type = "pearson")
>
> hist(residuals_RBC_filling,
+      main = "Histogram of Residuals for RBC_filling (Group T)",
+      xlab = "Residuals",
+      ylab = "Frequency",
+      col = "lightblue",
+      border = "black",
+      prob = TRUE)
> curve(dnorm(x, mean = mean(residuals_RBC_filling, na.rm = TRUE),
+             sd = sd(residuals_RBC_filling, na.rm = TRUE)),
+       col = "blue", lwd = 2, add = TRUE)
>

> exp(cbind(
+   Estimate = coef(glm_RBC_filling),
+   confint.default(glm_RBC_filling)
+ ))
            Estimate     2.5 %    97.5 %
(Intercept) 0.553398 0.4305674 0.7112691
Total_ATT   1.013304 0.9563434 1.0736577
```

Median_P50

```
> glm_Median_P50 <- glm(Median_P50 ~ Total_ATT,
+                        data = subset_data_T,
+                        family = Gamma(link = "log"))
> summary(glm_Median_P50)

Call:
glm(formula = Median_P50 ~ Total_ATT, family = Gamma(link = "log"),
    data = subset_data_T)

Coefficients:
```

```
             Estimate Std. Error t value Pr(>|t|)    
(Intercept)  2.27506    0.13229  17.197 3.42e-08 ***
Total_ATT   -0.03826    0.03050  -1.255    0.241    
---
Signif. codes:  0 ‘***’ 0.001 ‘**’ 0.01 ‘*’ 0.05 ‘.’ 0.1 ‘ ’ 1

(Dispersion parameter for Gamma family taken to be 0.02130641)

    Null deviance: 0.23340  on 10  degrees of freedom
Residual deviance: 0.19776  on  9  degrees of freedom
AIC: 39.455

Number of Fisher Scoring iterations: 4

> 
> residuals_Median_P50 <- residuals(glm_Median_P50, type = "pearson")
> 
> hist(residuals_Median_P50,
+      main = "Histogram of Residuals for Median_P50 (Group T)",
+      xlab = "Residuals",
+      ylab = "Frequency",
+      col = "lightblue",
+      border = "black",
+      prob = TRUE)
> curve(dnorm(x, mean = mean(residuals_Median_P50, na.rm = TRUE),
+             sd = sd(residuals_Median_P50, na.rm = TRUE)),
+       col = "blue", lwd = 2, add = TRUE)
> 

> exp(cbind(
+   Estimate = coef(glm_Median_P50),
+   confint.default(glm_Median_P50)
+ ))
             Estimate     2.5 %    97.5 %
(Intercept) 9.7284791 7.5064761 12.608220
Total_ATT   0.9624615 0.9066186  1.021744
```

PBR525

```
> glm_PBR525 <- glm(PBR525 ~ Total_ATT,
+                   data = subset_data_T,
+                   family = Gamma(link = "log"))
> summary(glm_PBR525)

Call:
glm(formula = PBR525 ~ Total_ATT, family = Gamma(link = "log"), 
    data = subset_data_T)

Coefficients:
             Estimate Std. Error t value Pr(>|t|)    
(Intercept)  0.993916   0.079714  12.468 5.55e-07 ***
Total_ATT   -0.009211   0.018376  -0.501    0.628    
---
Signif. codes:  0 ‘***’ 0.001 ‘**’ 0.01 ‘*’ 0.05 ‘.’ 0.1 ‘ ’ 1

(Dispersion parameter for Gamma family taken to be 0.007735741)

    Null deviance: 0.072208  on 10  degrees of freedom
Residual deviance: 0.070175  on  9  degrees of freedom
AIC: 2.594

Number of Fisher Scoring iterations: 4

> 
> residuals_PBR525 <- residuals(glm_PBR525, type = "pearson")
> 
> hist(residuals_PBR525,
+      main = "Histogram of Residuals for PBR525 (Group T)",
+      xlab = "Residuals",
```

```
+      ylab = "Frequency",
+      col = "lightblue",
+      border = "black",
+      prob = TRUE)
> curve(dnorm(x, mean = mean(residuals_PBR525, na.rm = TRUE),
+              sd = sd(residuals_PBR525, na.rm = TRUE)),
+       col = "blue", lwd = 2, add = TRUE)
>

> exp(cbind(
+   Estimate = coef(glm_PBR525),
+   confint.default(glm_PBR525)
+ ))
             Estimate     2.5 %   97.5 %
(Intercept) 2.7017950 2.3109977 3.158677
Total_ATT   0.9908312 0.9557804 1.027167
```

PBR59

```
> glm_PBR59 <- glm(PBR59 ~ Total_ATT,
+                  data = subset_data_T,
+                  family = Gamma(link = "log"))
> summary(glm_PBR59)

Call:
glm(formula = PBR59 ~ Total_ATT, family = Gamma(link = "log"),
    data = subset_data_T)

Coefficients:
             Estimate Std. Error t value Pr(>|t|)
(Intercept)  0.310531   0.124237   2.500   0.0339 *
Total_ATT   -0.003707   0.028639  -0.129   0.8998
---
Signif. codes:  0 ‘***’ 0.001 ‘**’ 0.01 ‘*’ 0.05 ‘.’ 0.1 ‘ ’ 1

(Dispersion parameter for Gamma family taken to be 0.01879031)

    Null deviance: 0.18533  on 10  degrees of freedom
Residual deviance: 0.18499  on  9  degrees of freedom
AIC: -1.3786

Number of Fisher Scoring iterations: 4

>
> residuals_PBR59 <- residuals(glm_PBR59, type = "pearson")
>
> hist(residuals_PBR59,
+      main = "Histogram of Residuals for PBR59 (Group T)",
+      xlab = "Residuals",
+      ylab = "Frequency",
+      col = "lightblue",
+      border = "black",
+      prob = TRUE)
> curve(dnorm(x, mean = mean(residuals_PBR59, na.rm = TRUE),
+              sd = sd(residuals_PBR59, na.rm = TRUE)),
+       col = "blue", lwd = 2, add = TRUE)
>

> exp(cbind(
+   Estimate = coef(glm_PBR59),
+   confint.default(glm_PBR59)
+ ))
             Estimate     2.5 %   97.5 %
(Intercept) 1.3641495 1.0693282 1.740255
Total_ATT   0.9962994 0.9419156 1.053823
```

PBR1019

```
> glm_PBR1019 <- glm(PBR1019 ~ Total_ATT,
```

```
+                    data = subset_data_T,
+                    family = Gamma(link = "log"))
> summary(glm_PBR1019)

Call:
glm(formula = PBR1019 ~ Total_ATT, family = Gamma(link = "log"),
    data = subset_data_T)

Coefficients:
            Estimate Std. Error t value Pr(>|t|)
(Intercept)  1.12828    0.05791  19.483 1.14e-08 ***
Total_ATT   -0.00544    0.01335  -0.407    0.693
---
Signif. codes:  0 ‘***’ 0.001 ‘**’ 0.01 ‘*’ 0.05 ‘.’ 0.1 ‘ ’ 1

(Dispersion parameter for Gamma family taken to be 0.0040827)

    Null deviance: 0.037542  on 10  degrees of freedom
Residual deviance: 0.036842  on  9  degrees of freedom
AIC: -1.1705

Number of Fisher Scoring iterations: 4

>
> residuals_PBR1019 <- residuals(glm_PBR1019, type = "pearson")
>
> hist(residuals_PBR1019,
+      main = "Histogram of Residuals for PBR1019 (Group T)",
+      xlab = "Residuals",
+      ylab = "Frequency",
+      col = "lightblue",
+      border = "black",
+      prob = TRUE)
> curve(dnorm(x, mean = mean(residuals_PBR1019, na.rm = TRUE),
+             sd = sd(residuals_PBR1019, na.rm = TRUE)),
+       col = "blue", lwd = 2, add = TRUE)
>

> exp(cbind(
+   Estimate = coef(glm_PBR1019),
+   confint.default(glm_PBR1019)
+ ))
            Estimate     2.5 %   97.5 %
(Intercept) 3.090340 2.7587519 3.461784
Total_ATT   0.994575 0.9688896 1.020941
```

PBR2025

```
> glm_PBR2025 <- glm(PBR2025 ~ Total_ATT,
+                    data = subset_data_T,
+                    family = Gamma(link = "log"))
> summary(glm_PBR2025)

Call:
glm(formula = PBR2025 ~ Total_ATT, family = Gamma(link = "log"),
    data = subset_data_T)

Coefficients:
            Estimate Std. Error t value Pr(>|t|)
(Intercept)  1.18286    0.11615  10.184 3.07e-06 ***
Total_ATT   -0.01594    0.02677  -0.595    0.566
---
Signif. codes:  0 ‘***’ 0.001 ‘**’ 0.01 ‘*’ 0.05 ‘.’ 0.1 ‘ ’ 1

(Dispersion parameter for Gamma family taken to be 0.01642222)

    Null deviance: 0.15475  on 10  degrees of freedom
Residual deviance: 0.14862  on  9  degrees of freedom
AIC: 14.334
```

```
Number of Fisher Scoring iterations: 4

>
> residuals_PBR2025 <- residuals(glm_PBR2025, type = "pearson")
>
> hist(residuals_PBR2025,
+      main = "Histogram of Residuals for PBR2025 (Group T)",
+      xlab = "Residuals",
+      ylab = "Frequency",
+      col = "lightblue",
+      border = "black",
+      prob = TRUE)
> curve(dnorm(x, mean = mean(residuals_PBR2025, na.rm = TRUE),
+             sd = sd(residuals_PBR2025, na.rm = TRUE)),
+       col = "blue", lwd = 2, add = TRUE)

> exp(cbind(
+   Estimate = coef(glm_PBR2025),
+   confint.default(glm_PBR2025)
+ ))
             Estimate    2.5 %   97.5 %
(Intercept) 3.2636920 2.599239 4.098001
Total_ATT   0.9841843 0.933870 1.037209
```

Glycocheck-TM vs PCV - Generalized linear models

```
> Glycoheck_clinical_study_dataset_for_stats$PCV <-
as.numeric(as.character(Glycoheck_clinical_study_dataset_for_stats$PCV))
Warning message:
NAs introduced by coercion
```

VVD

```
> glm_VVD <- glm(VVD ~ PCV,
+                data = Glycoheck_clinical_study_dataset_for_stats,
+                family = Gamma(link = "log"))
> summary(glm_VVD)

Call:
glm(formula = VVD ~ PCV, family = Gamma(link = "log"), data =
Glycoheck_clinical_study_dataset_for_stats)

Coefficients:
            Estimate Std. Error t value Pr(>|t|)
(Intercept) 5.451805   0.235677  23.133    1e-13 ***
PCV         0.015037   0.006879   2.186    0.044 *
---
Signif. codes:  0 ‘***’ 0.001 ‘**’ 0.01 ‘*’ 0.05 ‘.’ 0.1 ‘ ’ 1

(Dispersion parameter for Gamma family taken to be 0.08142635)

    Null deviance: 1.6208  on 17  degrees of freedom
Residual deviance: 1.2648  on 16  degrees of freedom
  (1 observation deleted due to missingness)
AIC: 222.27

Number of Fisher Scoring iterations: 4

>
> residuals_VVD <- residuals(glm_VVD, type = "pearson")
>
> hist(residuals_VVD,
+      main = "Histogram of Residuals for VVD",
+      xlab = "Residuals",
+      ylab = "Frequency",
+      col = "lightblue",
+      border = "black",
```

```
+      prob = TRUE)
> curve(dnorm(x, mean = mean(residuals_VVD, na.rm = TRUE),
+              sd = sd(residuals_VVD, na.rm = TRUE)),
+       col = "blue", lwd = 2, add = TRUE)

> exp(cbind(
+   Estimate = coef(glm_VVD),
+   confint.default(glm_VVD)
+ ))
              Estimate      2.5 %
(Intercept) 233.178637 146.919725
PCV           1.015151   1.001555
                97.5 %
(Intercept) 370.081532
PCV           1.028931

> confint(glm_VVD)
Waiting for profiling to be done...                      2.5 %     97.5 %
(Intercept) 4.9837266953 5.94099603
PCV         0.0009473079 0.02897528
```

RBC_filling

```
> glm_RBC_filling <- glm(RBC_filling ~ PCV,
+                        data =
Glycoheck_clinical_study_dataset_for_stats,
+                        family = Gamma(link = "log"))
> summary(glm_RBC_filling)

Call:
glm(formula = RBC_filling ~ PCV, family = Gamma(link = "log"),
    data = Glycoheck_clinical_study_dataset_for_stats)

Coefficients:
             Estimate Std. Error t value Pr(>|t|)
(Intercept) -0.675719   0.114263  -5.914 2.18e-05 ***
PCV          0.004197   0.003335   1.258    0.226
---
Signif. codes:  0 '***' 0.001 '**' 0.01 '*' 0.05 '.' 0.1 ' ' 1

(Dispersion parameter for Gamma family taken to be 0.01913982)

    Null deviance: 0.33353  on 17  degrees of freedom
Residual deviance: 0.30408  on 16  degrees of freedom
  (1 observation deleted due to missingness)
AIC: -35.991

Number of Fisher Scoring iterations: 4

>
> residuals_RBC_filling <- residuals(glm_RBC_filling, type = "pearson")
>
> hist(residuals_RBC_filling,
+      main = "Histogram of Residuals for RBC_filling",
+      xlab = "Residuals",
+      ylab = "Frequency",
+      col = "lightblue",
+      border = "black",
+      prob = TRUE)
> curve(dnorm(x, mean = mean(residuals_RBC_filling, na.rm = TRUE),
+              sd = sd(residuals_RBC_filling, na.rm = TRUE)),
+       col = "blue", lwd = 2, add = TRUE)

> exp(cbind(
+   Estimate = coef(glm_RBC_filling),
+   confint.default(glm_RBC_filling)
+ ))
             Estimate     2.5 %    97.5 %
```

```
(Intercept) 0.5087902 0.4067039 0.6365011
PCV         1.0042060 0.9976628 1.0107922

>
```

Median_P50

```
> glm_Median_P50 <- glm(Median_P50 ~ PCV,
+                       data = Glycoheck_clinical_study_dataset_for_stats,
+                       family = Gamma(link = "log"))
> summary(glm_Median_P50)

Call:
glm(formula = Median_P50 ~ PCV, family = Gamma(link = "log"),
    data = Glycoheck_clinical_study_dataset_for_stats)

Coefficients:
             Estimate Std. Error t value Pr(>|t|)
(Intercept)  2.239665   0.113415   19.75 1.16e-12 ***
PCV         -0.004007   0.003311   -1.21    0.244
---
Signif. codes:  0 ‘***’ 0.001 ‘**’ 0.01 ‘*’ 0.05 ‘.’ 0.1 ‘ ’ 1

(Dispersion parameter for Gamma family taken to be 0.01885681)

    Null deviance: 0.33247  on 17  degrees of freedom
Residual deviance: 0.30543  on 16  degrees of freedom
  (1 observation deleted due to missingness)
AIC: 59.344

Number of Fisher Scoring iterations: 4

>
> residuals_Median_P50 <- residuals(glm_Median_P50, type = "pearson")
>
> hist(residuals_Median_P50,
+      main = "Histogram of Residuals for Median_P50",
+      xlab = "Residuals",
+      ylab = "Frequency",
+      col = "lightblue",
+      border = "black",
+      prob = TRUE)
> curve(dnorm(x, mean = mean(residuals_Median_P50, na.rm = TRUE),
+             sd = sd(residuals_Median_P50, na.rm = TRUE)),
+       col = "blue", lwd = 2, add = TRUE)

> exp(cbind(
+   Estimate = coef(glm_Median_P50),
+   confint.default(glm_Median_P50)
+ ))
             Estimate     2.5 %    97.5 %
(Intercept) 9.3901887 7.5185770 11.727704
PCV         0.9960014 0.9895596  1.002485

>
```

PBR525

```
> glm_PBR525 <- glm(PBR525 ~ PCV,
+                   data = Glycoheck_clinical_study_dataset_for_stats,
+                   family = Gamma(link = "log"))
> summary(glm_PBR525)

Call:
glm(formula = PBR525 ~ PCV, family = Gamma(link = "log"), data =
Glycoheck_clinical_study_dataset_for_stats)

Coefficients:
             Estimate Std. Error t value Pr(>|t|)
(Intercept)  0.987703   0.073182  13.497 3.68e-10 ***
PCV         -0.000882   0.002136  -0.413    0.685
```

```
---
Signif. codes:  0 ‘***’ 0.001 ‘**’ 0.01 ‘*’ 0.05 ‘.’ 0.1 ‘ ’ 1

(Dispersion parameter for Gamma family taken to be 0.007851244)

    Null deviance: 0.12568  on 17  degrees of freedom
Residual deviance: 0.12433  on 16  degrees of freedom
  (1 observation deleted due to missingness)
AIC: 1.9399

Number of Fisher Scoring iterations: 3

>
> residuals_PBR525 <- residuals(glm_PBR525, type = "pearson")
>
> hist(residuals_PBR525,
+      main = "Histogram of Residuals for PBR525",
+      xlab = "Residuals",
+      ylab = "Frequency",
+      col = "lightblue",
+      border = "black",
+      prob = TRUE)
> curve(dnorm(x, mean = mean(residuals_PBR525, na.rm = TRUE),
+             sd = sd(residuals_PBR525, na.rm = TRUE)),
+       col = "blue", lwd = 2, add = TRUE)
>

> exp(cbind(
+   Estimate = coef(glm_PBR525),
+   confint.default(glm_PBR525)
+ ))
             Estimate    2.5 %   97.5 %
(Intercept) 2.6850608 2.326278 3.099179
PCV         0.9991184 0.994944 1.003310
```

PBR59

```
> glm_PBR59 <- glm(PBR59 ~ PCV,
+                  data = Glycoheck_clinical_study_dataset_for_stats,
+                  family = Gamma(link = "log"))
> summary(glm_PBR59)

Call:
glm(formula = PBR59 ~ PCV, family = Gamma(link = "log"), data =
Glycoheck_clinical_study_dataset_for_stats)

Coefficients:
              Estimate Std. Error t value Pr(>|t|)
(Intercept)  3.219e-01  9.519e-02   3.382  0.00381 **
PCV         -7.221e-05  2.779e-03  -0.026  0.97959
---
Signif. codes:  0 ‘***’ 0.001 ‘**’ 0.01 ‘*’ 0.05 ‘.’ 0.1 ‘ ’ 1

(Dispersion parameter for Gamma family taken to be 0.01328458)

    Null deviance: 0.23343  on 17  degrees of freedom
Residual deviance: 0.23342  on 16  degrees of freedom
  (1 observation deleted due to missingness)
AIC: -9.8248

Number of Fisher Scoring iterations: 4

>
> residuals_PBR59 <- residuals(glm_PBR59, type = "pearson")
>
> hist(residuals_PBR59,
+      main = "Histogram of Residuals for PBR59",
+      xlab = "Residuals",
+      ylab = "Frequency",
```

```
+      col = "lightblue",
+      border = "black",
+      prob = TRUE)
> curve(dnorm(x, mean = mean(residuals_PBR59, na.rm = TRUE),
+             sd = sd(residuals_PBR59, na.rm = TRUE)),
+       col = "blue", lwd = 2, add = TRUE)

> exp(cbind(
+   Estimate = coef(glm_PBR59),
+   confint.default(glm_PBR59)
+ ))
             Estimate     2.5 %   97.5 %
(Intercept) 1.3797484 1.1449085 1.662758
PCV         0.9999278 0.9944968 1.005388

>
```

PBR1019

```
> glm_PBR1019 <- glm(PBR1019 ~ PCV,
+                    data = Glycoheck_clinical_study_dataset_for_stats,
+                    family = Gamma(link = "log"))
> summary(glm_PBR1019)

Call:
glm(formula = PBR1019 ~ PCV, family = Gamma(link = "log"), data =
Glycoheck_clinical_study_dataset_for_stats)

Coefficients:
              Estimate Std. Error t value Pr(>|t|)
(Intercept)  1.1154077  0.0643281  17.339 8.54e-12 ***
PCV         -0.0003093  0.0018778  -0.165    0.871
---
Signif. codes:  0 ‘***’ 0.001 ‘**’ 0.01 ‘*’ 0.05 ‘.’ 0.1 ‘ ’ 1

(Dispersion parameter for Gamma family taken to be 0.006066403)

    Null deviance: 0.095729  on 17  degrees of freedom
Residual deviance: 0.095562  on 16  degrees of freedom
  (1 observation deleted due to missingness)
AIC: 2.5009

Number of Fisher Scoring iterations: 4

>
> residuals_PBR1019 <- residuals(glm_PBR1019, type = "pearson")
>
> hist(residuals_PBR1019,
+      main = "Histogram of Residuals for PBR1019",
+      xlab = "Residuals",
+      ylab = "Frequency",
+      col = "lightblue",
+      border = "black",
+      prob = TRUE)
> curve(dnorm(x, mean = mean(residuals_PBR1019, na.rm = TRUE),
+             sd = sd(residuals_PBR1019, na.rm = TRUE)),
+       col = "blue", lwd = 2, add = TRUE)
>
> exp(cbind(
+   Estimate = coef(glm_PBR1019),
+   confint.default(glm_PBR1019)
+ ))
             Estimate     2.5 %   97.5 %
(Intercept) 3.0508116 2.6894239 3.460760
PCV         0.9996907 0.9960183 1.003377
```

PBR2025

```
> glm_PBR2025 <- glm(PBR2025 ~ PCV,
+                    data = Glycoheck_clinical_study_dataset_for_stats,
```

```
+                   family = Gamma(link = "log"))
> summary(glm_PBR2025)

Call:
glm(formula = PBR2025 ~ PCV, family = Gamma(link = "log"), data =
Glycoheck_clinical_study_dataset_for_stats)

Coefficients:
             Estimate Std. Error t value Pr(>|t|)
(Intercept)  1.196738   0.103576  11.554 3.55e-09 ***
PCV         -0.002453   0.003023  -0.811    0.429
---
Signif. codes:  0 '***' 0.001 '**' 0.01 '*' 0.05 '.' 0.1 ' ' 1

(Dispersion parameter for Gamma family taken to be 0.0157271)

    Null deviance: 0.27138  on 17  degrees of freedom
Residual deviance: 0.26104  on 16  degrees of freedom
  (1 observation deleted due to missingness)
AIC: 20.845

Number of Fisher Scoring iterations: 4

>
> residuals_PBR2025 <- residuals(glm_PBR2025, type = "pearson")
>
> hist(residuals_PBR2025,
+      main = "Histogram of Residuals for PBR2025",
+      xlab = "Residuals",
+      ylab = "Frequency",
+      col = "lightblue",
+      border = "black",
+      prob = TRUE)
> curve(dnorm(x, mean = mean(residuals_PBR2025, na.rm = TRUE),
+             sd = sd(residuals_PBR2025, na.rm = TRUE)),
+       col = "blue", lwd = 2, add = TRUE)

> exp(cbind(
+   Estimate = coef(glm_PBR2025),
+   confint.default(glm_PBR2025)
+ ))
             Estimate    2.5 %   97.5 %
(Intercept) 3.3093045 2.701299 4.054159
PCV         0.9975497 0.991656 1.003479
```

Glycocheck-TM vs clinical variables - linear models

```
> Glycoheck_clinical_study_dataset_for_stats <- read_excel("~/Scientific p
rojects/Glycocalyx/Glycocheck 2023/Glycocalyx clinical study/Raw data/Glyc
oheck_clinical_study_dataset_for_stats.xlsx")
> View(Glycoheck_clinical_study_dataset_for_stats)
```

VVD

```
> lm_VVD <- lm(VVD ~ Group + APPLE_fast + LOH,
+              data = Glycoheck_clinical_study_dataset_for_stats)
> summary(lm_VVD)

Call:
lm(formula = VVD ~ Group + APPLE_fast + LOH, data = Glycoheck_clinical_stu
dy_dataset_for_stats)

Residuals:
     Min       1Q   Median       3Q      Max 
-146.731  -44.282   -3.931   56.662  243.659 

Coefficients:
            Estimate Std. Error t value Pr(>|t|)   
(Intercept)  394.969    107.891   3.661  0.00232 **
GroupT       -99.710     51.282  -1.944  0.07085 . 
APPLE_fast     6.200      5.821   1.065  0.30368   
LOH          -14.692      8.564  -1.716  0.10682   
---
Signif. codes:  0 ‘***’ 0.001 ‘**’ 0.01 ‘*’ 0.05 ‘.’ 0.1 ‘ ’ 1

Residual standard error: 104.5 on 15 degrees of freedom
Multiple R-squared:  0.2763,	Adjusted R-squared:  0.1316 
F-statistic: 1.909 on 3 and 15 DF,  p-value: 0.1715


> residuals_VVD <- residuals(lm_VVD)
> shapiro.test(residuals_VVD)

	Shapiro-Wilk normality test

data:  residuals_VVD
W = 0.92867, p-value = 0.1637

> 
> hist(residuals_VVD,
+      main = "Histogram of Residuals for VVD",
+      xlab = "Residuals",
+      ylab = "Frequency",
+      col = "lightblue",
+      border = "black",
+      prob = TRUE)
> curve(dnorm(x, mean = mean(residuals_VVD, na.rm = TRUE),
+             sd = sd(residuals_VVD, na.rm = TRUE)),
+       col = "blue", lwd = 2, add = TRUE)

> #CI
> confint(lm_VVD, level = 0.95)
                  2.5 %     97.5 %
(Intercept)  165.004906 624.932837
GroupT      -209.014965   9.595799
APPLE_fast    -6.207729  18.608359
LOH          -32.944797   3.561772
```

RBC filling

```
> lm_RBC_filling <- lm(RBC_filling ~ Group + APPLE_fast + LOH,
+              data = Glycoheck_clinical_study_dataset_for_stats)
> summary(lm_RBC_filling)

Call:
```

```
lm(formula = RBC_filling ~ Group + APPLE_fast + LOH, data = Glycoheck_clin
ical_study_dataset_for_stats)

Residuals:
      Min        1Q    Median        3Q       Max 
-0.142852 -0.056215  0.001938  0.045186  0.146359 

Coefficients:
              Estimate Std. Error t value Pr(>|t|)    
(Intercept)  0.5939571  0.0884358   6.716 6.91e-06 ***
GroupT      -0.0120553  0.0420349  -0.287    0.778    
APPLE_fast   0.0009034  0.0047717   0.189    0.852    
LOH         -0.0042379  0.0070195  -0.604    0.555    
---
Signif. codes:  0 ‘***’ 0.001 ‘**’ 0.01 ‘*’ 0.05 ‘.’ 0.1 ‘ ’ 1

Residual standard error: 0.08569 on 15 degrees of freedom
Multiple R-squared:  0.02489,	Adjusted R-squared:  -0.1701 
F-statistic: 0.1276 on 3 and 15 DF,  p-value: 0.9422

> residuals_RBC_filling <- residuals(lm_RBC_filling)
> shapiro.test(residuals_RBC_filling)

	Shapiro-Wilk normality test

data:  residuals_RBC_filling
W = 0.96963, p-value = 0.769

> 
> hist(residuals_RBC_filling,
+      main = "Histogram of Residuals for RBC_filling",
+      xlab = "Residuals",
+      ylab = "Frequency",
+      col = "lightblue",
+      border = "black",
+      prob = TRUE)
> curve(dnorm(x, mean = mean(residuals_RBC_filling, na.rm = TRUE),
+              sd = sd(residuals_RBC_filling, na.rm = TRUE)),
+       col = "blue", lwd = 2, add = TRUE)

> #CI
> confint(lm_RBC_filling, level = 0.95)
                   2.5 %     97.5 %
(Intercept)  0.405460694 0.78245346
GroupT      -0.101650469 0.07753995
APPLE_fast  -0.009267218 0.01107398
LOH         -0.019199727 0.01072390
```

Median P50

```
> lm_Median_P50 <- lm(Median_P50 ~ Group + APPLE_fast + LOH,
+              data = Glycoheck_clinical_study_dataset_for_stats)
> summary(lm_Median_P50)

Call:
lm(formula = Median_P50 ~ Group + APPLE_fast + LOH, data = Glycoheck_clini
cal_study_dataset_for_stats)

Residuals:
     Min       1Q   Median       3Q      Max 
-1.75279 -0.80006  0.04269  0.32181  2.18400 

Coefficients:
             Estimate Std. Error t value Pr(>|t|)    
(Intercept)  8.466383   1.263868   6.699 7.12e-06 ***
GroupT       0.194759   0.600736   0.324    0.750    
APPLE_fast  -0.020149   0.068194  -0.295    0.772    
LOH          0.005065   0.100319   0.050    0.960    
---
```

```
Signif. codes:  0 ‘***’ 0.001 ‘**’ 0.01 ‘*’ 0.05 ‘.’ 0.1 ‘ ’ 1

Residual standard error: 1.225 on 15 degrees of freedom
Multiple R-squared:  0.01258, Adjusted R-squared:  -0.1849
F-statistic: 0.06368 on 3 and 15 DF,  p-value: 0.9782

> residuals_Median_P50 <- residuals(lm_Median_P50)
> shapiro.test(residuals_Median_P50)

        Shapiro-Wilk normality test

data:  residuals_Median_P50
W = 0.94892, p-value = 0.3787

>
> hist(residuals_Median_P50,
+      main = "Histogram of Residuals for Median_P50",
+      xlab = "Residuals",
+      ylab = "Frequency",
+      col = "lightblue",
+      border = "black",
+      prob = TRUE)
> curve(dnorm(x, mean = mean(residuals_Median_P50, na.rm = TRUE),
+             sd = sd(residuals_Median_P50, na.rm = TRUE)),
+       col = "blue", lwd = 2, add = TRUE)

> #CI
> confint(lm_Median_P50, level = 0.95)
                 2.5 %     97.5 %
(Intercept)  5.7725125 11.1602534
GroupT      -1.0856792  1.4751963
APPLE_fast  -0.1655012  0.1252023
LOH         -0.2087599  0.2188896
```

PBR525

```
> lm_PBR525 <- lm(PBR525 ~ Group + APPLE_fast + LOH,
+             data = Glycoheck_clinical_study_dataset_for_stats)
> summary(lm_PBR525)

Call:
lm(formula = PBR525 ~ Group + APPLE_fast + LOH, data = Glycoheck_clinical_
study_dataset_for_stats)

Residuals:
     Min       1Q   Median       3Q      Max
-0.32125 -0.14881 -0.07358  0.14838  0.41612

Coefficients:
            Estimate Std. Error t value Pr(>|t|)
(Intercept) 2.443161   0.244775   9.981 5.12e-08 ***
GroupT      0.004385   0.116345   0.038    0.970
APPLE_fast  0.008402   0.013207   0.636    0.534
LOH         0.003550   0.019429   0.183    0.857
---
Signif. codes:  0 ‘***’ 0.001 ‘**’ 0.01 ‘*’ 0.05 ‘.’ 0.1 ‘ ’ 1

Residual standard error: 0.2372 on 15 degrees of freedom
Multiple R-squared:  0.0315,  Adjusted R-squared:  -0.1622
F-statistic: 0.1626 on 3 and 15 DF,  p-value: 0.9198


> residuals_PBR525 <- residuals(lm_PBR525)
> shapiro.test(residuals_PBR525)

        Shapiro-Wilk normality test

data:  residuals_PBR525
W = 0.93523, p-value = 0.216
```

```
>
> hist(residuals_PBR525,
+      main = "Histogram of Residuals for PBR525",
+      xlab = "Residuals",
+      ylab = "Frequency",
+      col = "lightblue",
+      border = "black",
+      prob = TRUE)
> curve(dnorm(x, mean = mean(residuals_PBR525, na.rm = TRUE),
+             sd = sd(residuals_PBR525, na.rm = TRUE)),
+       col = "blue", lwd = 2, add = TRUE)


> #CI
> confint(lm_PBR525, level = 0.95)
                  2.5 %     97.5 %
(Intercept)  1.92143596 2.96488547
GroupT      -0.24359911 0.25236836
APPLE_fast  -0.01974811 0.03655274
LOH         -0.03786167 0.04496166
```

PBR 59

```
> lm_PBR59 <- lm(PBR59 ~ Group + APPLE_fast + LOH,
+              data = Glycoheck_clinical_study_dataset_for_stats)
> summary(lm_PBR59)

Call:
lm(formula = PBR59 ~ Group + APPLE_fast + LOH, data = Glycoheck_clinical_s
tudy_dataset_for_stats)

Residuals:
     Min       1Q   Median       3Q      Max 
-0.36125 -0.05652  0.01796  0.10011  0.22059 

Coefficients:
              Estimate Std. Error t value Pr(>|t|)    
(Intercept)  1.4562480  0.1622467   8.976 2.03e-07 ***
GroupT      -0.0877153  0.0771183  -1.137    0.273    
APPLE_fast  -0.0009651  0.0087543  -0.110    0.914    
LOH         -0.0027183  0.0128783  -0.211    0.836    
---
Signif. codes:  0 ‘***’ 0.001 ‘**’ 0.01 ‘*’ 0.05 ‘.’ 0.1 ‘ ’ 1

Residual standard error: 0.1572 on 15 degrees of freedom
Multiple R-squared:  0.08228,	Adjusted R-squared:  -0.1013 
F-statistic: 0.4483 on 3 and 15 DF,  p-value: 0.7222


> residuals_PBR59 <- residuals(lm_PBR59)
> shapiro.test(residuals_PBR59)

	Shapiro-Wilk normality test

data:  residuals_PBR59
W = 0.95038, p-value = 0.4012

>
> hist(residuals_PBR59,
+      main = "Histogram of Residuals for PBR59",
+      xlab = "Residuals",
+      ylab = "Frequency",
+      col = "lightblue",
+      border = "black",
+      prob = TRUE)
> curve(dnorm(x, mean = mean(residuals_PBR59, na.rm = TRUE),
+             sd = sd(residuals_PBR59, na.rm = TRUE)),
+       col = "blue", lwd = 2, add = TRUE)
```

```
> #CI
> confint(lm_PBR59, level = 0.95)
                  2.5 %     97.5 %
(Intercept)  1.11042729 1.80206865
GroupT      -0.25208916 0.07665853
APPLE_fast  -0.01962436 0.01769417
LOH         -0.03016762 0.02473109
```

PBR1019

```
> lm_PBR1019 <- lm(PBR1019 ~ Group + APPLE_fast + LOH,
+              data = Glycoheck_clinical_study_dataset_for_stats)
> summary(lm_PBR1019)

Call:
lm(formula = PBR1019 ~ Group + APPLE_fast + LOH, data = Glycoheck_clinical
_study_dataset_for_stats)

Residuals:
     Min       1Q   Median       3Q      Max
-0.30367 -0.17069 -0.02806  0.12863  0.41671

Coefficients:
            Estimate Std. Error t value Pr(>|t|)
(Intercept) 2.909292   0.248114  11.726 5.93e-09 ***
GroupT      0.033054   0.117932   0.280    0.783
APPLE_fast  0.002825   0.013387   0.211    0.836
LOH         0.010286   0.019694   0.522    0.609
---
Signif. codes:  0 ‘***’ 0.001 ‘**’ 0.01 ‘*’ 0.05 ‘.’ 0.1 ‘ ’ 1

Residual standard error: 0.2404 on 15 degrees of freedom
Multiple R-squared:  0.0245,  Adjusted R-squared:  -0.1706
F-statistic: 0.1256 on 3 and 15 DF,  p-value: 0.9435


> residuals_PBR1019 <- residuals(lm_PBR1019)
> shapiro.test(residuals_PBR1019)

        Shapiro-Wilk normality test

data:  residuals_PBR1019
W = 0.94168, p-value = 0.2827

>
> hist(residuals_PBR1019,
+      main = "Histogram of Residuals for PBR1019",
+      xlab = "Residuals",
+      ylab = "Frequency",
+      col = "lightblue",
+      border = "black",
+      prob = TRUE)
> curve(dnorm(x, mean = mean(residuals_PBR1019, na.rm = TRUE),
+             sd = sd(residuals_PBR1019, na.rm = TRUE)),
+       col = "blue", lwd = 2, add = TRUE)


> #CI
> confint(lm_PBR1019, level = 0.95)
                  2.5 %     97.5 %
(Intercept)  2.38045050 3.43813428
GroupT      -0.21831270 0.28442054
APPLE_fast  -0.02570923 0.03135966
LOH         -0.03169079 0.05226238
```

PBR525

```
> lm_PBR2025 <- lm(PBR2025 ~ Group + APPLE_fast + LOH,
+               data = Glycoheck_clinical_study_dataset_for_stats)
> summary(lm_PBR2025)

Call:
lm(formula = PBR2025 ~ Group + APPLE_fast + LOH, data = Glycoheck_clinical
_study_dataset_for_stats)

Residuals:
     Min       1Q   Median       3Q      Max 
-0.61557 -0.35139  0.01117  0.29533  0.66117 

Coefficients:
            Estimate Std. Error t value Pr(>|t|)    
(Intercept) 2.542722   0.406091   6.261 1.52e-05 ***
GroupT      0.062643   0.193021   0.325    0.750    
APPLE_fast  0.025895   0.021911   1.182    0.256    
LOH         0.003411   0.032233   0.106    0.917    
---
Signif. codes:  0 ‘***’ 0.001 ‘**’ 0.01 ‘*’ 0.05 ‘.’ 0.1 ‘ ’ 1

Residual standard error: 0.3935 on 15 degrees of freedom
Multiple R-squared:  0.09573,	Adjusted R-squared:  -0.08512 
F-statistic: 0.5293 on 3 and 15 DF,  p-value: 0.6689

> residuals_PBR2025 <- residuals(lm_PBR2025)
> shapiro.test(residuals_PBR2025)

	Shapiro-Wilk normality test

data:  residuals_PBR2025
W = 0.95122, p-value = 0.4144

> 
> hist(residuals_PBR2025,
+      main = "Histogram of Residuals for PBR2025",
+      xlab = "Residuals",
+      ylab = "Frequency",
+      col = "lightblue",
+      border = "black",
+      prob = TRUE)
> curve(dnorm(x, mean = mean(residuals_PBR2025, na.rm = TRUE),
+             sd = sd(residuals_PBR2025, na.rm = TRUE)),
+       col = "blue", lwd = 2, add = TRUE)

> #CI
> confint(lm_PBR2025, level = 0.95)
                  2.5 %     97.5 %
(Intercept)  1.67715987 3.40828332
GroupT      -0.34877128 0.47405812
APPLE_fast  -0.02080728 0.07259803
LOH         -0.06529215 0.07211498
```

Confidence intervals for linear models

```
> Glycocheck_clinical_study_dataset_for_stats <- read_excel("~/Scientific
projects/Glycocalyx/Glycocheck 2023/Glycocalyx clinical study/Raw
data/Glycocheck_clinical_study_dataset_for_stats.xlsx")
> View(Glycoheck_clinical_study_dataset_for_stats)
Error in View : object 'Glycoheck_clinical_study_dataset_for_stats' not
found
> View(Glycocheck_clinical_study_dataset_for_stats)

> #VVD
```

```
> lm_VVD <- lm(VVD ~ Group + APPLE_fast + LOH, data =
Glycocheck_clinical_study_dataset_for_stats)
> summary(lm_VVD)

Call:
lm(formula = VVD ~ Group + APPLE_fast + LOH, data =
Glycocheck_clinical_study_dataset_for_stats)

Residuals:
     Min       1Q   Median       3Q
-146.731  -44.282   -3.931   56.662
     Max
 243.659

Coefficients:
            Estimate Std. Error t value
(Intercept)  394.969    107.891   3.661
GroupT       -99.710     51.282  -1.944
APPLE_fast     6.200      5.821   1.065
LOH          -14.692      8.564  -1.716
            Pr(>|t|)
(Intercept)  0.00232 **
GroupT       0.07085 .
APPLE_fast   0.30368
LOH          0.10682
---
Signif. codes:
  0 ‘***’ 0.001 ‘**’ 0.01 ‘*’ 0.05 ‘.’
  0.1 ‘ ’ 1

Residual standard error: 104.5 on 15 degrees of freedom
Multiple R-squared:  0.2763,  Adjusted R-squared:  0.1316
F-statistic: 1.909 on 3 and 15 DF,  p-value: 0.1715

> #CI
> confint(lm_VVD, level = 0.95)
                  2.5 %     97.5 %
(Intercept)  165.004906 624.932837
GroupT      -209.014965   9.595799
APPLE_fast    -6.207729  18.608359
LOH          -32.944797   3.561772
> confint(lm_RBC_filling, level = 0.95)
Error: object 'lm_RBC_filling' not found

> #RBC filling
> lm_RBC_filling <- lm(RBC_filling ~ Group + APPLE_fast + LOH,data =
Glycocheck_clinical_study_dataset_for_stats)
> confint(lm_RBC_filling, level = 0.95)
                   2.5 %     97.5 %
(Intercept)  0.405460694 0.78245346
GroupT      -0.101650469 0.07753995
APPLE_fast  -0.009267218 0.01107398
LOH         -0.019199727 0.01072390
> #Median P50
> lm_Median_P50 <- lm(Median_P50 ~ Group + APPLE_fast + LOH,data =
Glycocheck_clinical_study_dataset_for_stats)
> confint)lm_Median_P50, level = 0.95)
Error: unexpected ')' in "confint)"

> confint(lm_Median_P50, level = 0.95)
                 2.5 %     97.5 %
(Intercept)  5.7725125 11.1602534
GroupT      -1.0856792  1.4751963
APPLE_fast  -0.1655012  0.1252023
LOH         -0.2087599  0.2188896
> #PBR 5-25
> lm_PBR525 <- lm(PBR525 ~ Group + APPLE_fast + LOH,data =
Glycocheck_clinical_study_dataset_for_stats)
> confint(lm_PBR525, level = 0.95)
```

```
                  2.5 %     97.5 %
(Intercept)  1.92143596 2.96488547
GroupT      -0.24359911 0.25236836
APPLE_fast  -0.01974811 0.03655274
LOH         -0.03786167 0.04496166
>
> #PBR 5-9
> lm_PBR59 <- lm(PBR59 ~ Group + APPLE_fast + LOH,data =
Glycocheck_clinical_study_dataset_for_stats)
> confint(lm_PBR59, level = 0.95)
                  2.5 %     97.5 %
(Intercept)  1.11042729 1.80206865
GroupT      -0.25208916 0.07665853
APPLE_fast  -0.01962436 0.01769417
LOH         -0.03016762 0.02473109
> #PBR 10-19
> lm_PBR1019 <- lm(PBR1019 ~ Group + APPLE_fast + LOH,data =
Glycocheck_clinical_study_dataset_for_stats)
> confint(lm_PBR1019, level = 0.95)
                  2.5 %     97.5 %
(Intercept)  2.38045050 3.43813428
GroupT      -0.21831270 0.28442054
APPLE_fast  -0.02570923 0.03135966
LOH         -0.03169079 0.05226238
> #PBR 20-25
> lm_PBR2025 <- lm(PBR2025 ~ Group + APPLE_fast + LOH,data =
Glycocheck_clinical_study_dataset_for_stats)
> confint(lm_PBR2025, level = 0.95)
                  2.5 %     97.5 %
(Intercept)  1.67715987 3.40828332
GroupT      -0.34877128 0.47405812
APPLE_fast  -0.02080728 0.07259803
LOH         -0.06529215 0.07211498
```

Glycocheck-TM vs IV fluid variables - Linear models

```
> subset_data <- subset(Glycoheck_clinical_study_dataset_for_stats,
IV_fluids_Y_N == "Y")
```

VVD

```
> lm_VVD <- lm(VVD ~ IV_fluids_rate + IV_fluids_duration +
IV_fluids_total,
+              data = subset_data)
>
> summary(lm_VVD)

Call:
lm(formula = VVD ~ IV_fluids_rate + IV_fluids_duration + IV_fluids_total,
    data = subset_data)

Residuals:
    Min      1Q  Median      3Q     Max
-107.15  -59.05  -12.19   67.21  134.03

Coefficients:
                   Estimate Std. Error t value Pr(>|t|)
(Intercept)        -33.1305   145.9701  -0.227   0.8280
IV_fluids_rate      28.7590     9.1348   3.148   0.0199 *
IV_fluids_duration  19.3932     8.2261   2.358   0.0565 .
IV_fluids_total     -1.2974     0.5447  -2.382   0.0546 .
---
Signif. codes:  0 ‘***’ 0.001 ‘**’ 0.01 ‘*’ 0.05 ‘.’ 0.1 ‘ ’ 1

Residual standard error: 104.4 on 6 degrees of freedom
Multiple R-squared:  0.6303,  Adjusted R-squared:  0.4454
F-statistic: 3.409 on 3 and 6 DF,  p-value: 0.09389
```

```
> residuals_VVD <- residuals(lm_VVD)
>
> shapiro.test(residuals_VVD)

      Shapiro-Wilk normality test

data:  residuals_VVD
W = 0.92205, p-value = 0.3744


> hist(residuals_VVD,
+      main = "Histogram of Residuals for VVD (Group Y)",
+      xlab = "Residuals",
+      ylab = "Frequency",
+      col = "lightblue",
+      border = "black",
+      prob = TRUE)
>
> curve(dnorm(x, mean = mean(residuals_VVD, na.rm = TRUE),
+             sd = sd(residuals_VVD, na.rm = TRUE)),
+       col = "blue", lwd = 2, add = TRUE)
```

RBC filling

```
> lm_RBC_filling <- lm(RBC_filling ~ IV_fluids_rate + IV_fluids_duration +
IV_fluids_total,
+              data = subset_data)
> summary(lm_RBC_filling)

Call:
lm(formula = RBC_filling ~ IV_fluids_rate + IV_fluids_duration +
    IV_fluids_total, data = subset_data)

Residuals:
     Min       1Q   Median       3Q      Max
-0.08624 -0.04170 -0.01628  0.01068  0.17852

Coefficients:
                     Estimate Std. Error t value Pr(>|t|)
(Intercept)         0.4214946  0.1260183   3.345   0.0155 *
IV_fluids_rate      0.0133548  0.0078863   1.693   0.1413
IV_fluids_duration  0.0058195  0.0071017   0.819   0.4439
IV_fluids_total    -0.0004660  0.0004703  -0.991   0.3600
---
Signif. codes:  0 ‘***’ 0.001 ‘**’ 0.01 ‘*’ 0.05 ‘.’ 0.1 ‘ ’ 1

Residual standard error: 0.09014 on 6 degrees of freedom
Multiple R-squared:  0.3981,  Adjusted R-squared:  0.09721
F-statistic: 1.323 on 3 and 6 DF,  p-value: 0.3514

> residuals_RBC_filling <- residuals(lm_RBC_filling)
>
> shapiro.test(residuals_RBC_filling)

      Shapiro-Wilk normality test

data:  residuals_RBC_filling
W = 0.84966, p-value = 0.05755

> hist(residuals_RBC_filling,
+      main = "Histogram of Residuals for RBC_filling (Group Y)",
+      xlab = "Residuals",
+      ylab = "Frequency",
+      col = "lightblue",
+      border = "black",
+      prob = TRUE)
>
> curve(dnorm(x, mean = mean(residuals_RBC_filling, na.rm = TRUE),
+             sd = sd(residuals_RBC_filling, na.rm = TRUE)),
```

```
+       col = "blue", lwd = 2, add = TRUE)
```

Median P50

```
> lm_Median_P50 <- lm(Median_P50 ~ IV_fluids_rate + IV_fluids_duration +
IV_fluids_total,
+               data = subset_data)
> summary(lm_Median_P50)

Call:
lm(formula = Median_P50 ~ IV_fluids_rate + IV_fluids_duration +
    IV_fluids_total, data = subset_data)

Residuals:
     Min       1Q   Median       3Q      Max
-1.09432 -0.26607 -0.05857  0.29582  0.83716

Coefficients:
                    Estimate Std. Error t value Pr(>|t|)
(Intercept)        10.816740   0.945579  11.439 2.68e-05 ***
IV_fluids_rate     -0.155693   0.059175  -2.631   0.0390 *
IV_fluids_duration -0.163159   0.053288  -3.062   0.0222 *
IV_fluids_total     0.009643   0.003529   2.733   0.0341 *
---
Signif. codes:  0 ‘***’ 0.001 ‘**’ 0.01 ‘*’ 0.05 ‘.’ 0.1 ‘ ’ 1

Residual standard error: 0.6764 on 6 degrees of freedom
Multiple R-squared:  0.615,   Adjusted R-squared:  0.4225
F-statistic: 3.195 on 3 and 6 DF,  p-value: 0.1051

> residuals_Median_P50 <- residuals(lm_Median_P50)
> shapiro.test(residuals_Median_P50)

      Shapiro-Wilk normality test

data:  residuals_Median_P50
W = 0.9629, p-value = 0.8184

> hist(residuals_Median_P50,
+      main = "Histogram of Residuals for Median_P50 (Group Y)",
+      xlab = "Residuals",
+      ylab = "Frequency",
+      col = "lightblue",
+      border = "black",
+      prob = TRUE)

> curve(dnorm(x, mean = mean(residuals_Median_P50, na.rm = TRUE),
+             sd = sd(residuals_Median_P50, na.rm = TRUE)),
+       col = "blue", lwd = 2, add = TRUE)
```

PBR525

```
> lm_PBR525 <- lm(PBR525 ~ IV_fluids_rate + IV_fluids_duration +
IV_fluids_total,
+               data = subset_data)
> summary(lm_PBR525)

Call:
lm(formula = PBR525 ~ IV_fluids_rate + IV_fluids_duration +
IV_fluids_total,
    data = subset_data)

Residuals:
     Min       1Q   Median       3Q      Max
-0.35738 -0.08553 -0.02319  0.08879  0.36206

Coefficients:
                     Estimate Std. Error t value Pr(>|t|)
```

```
(Intercept)         2.8261315  0.3347259   8.443 0.000151 ***
IV_fluids_rate     -0.0225727  0.0209472  -1.078 0.322618
IV_fluids_duration -0.0081065  0.0188634  -0.430 0.682384
IV_fluids_total     0.0008251  0.0012491   0.661 0.533398
---
Signif. codes:  0 ‘***’ 0.001 ‘**’ 0.01 ‘*’ 0.05 ‘.’ 0.1 ‘ ’ 1

Residual standard error: 0.2394 on 6 degrees of freedom
Multiple R-squared:  0.2414,  Adjusted R-squared:  -0.1378
F-statistic: 0.6366 on 3 and 6 DF,  p-value: 0.6184

> residuals_PBR525 <- residuals(lm_PBR525)

> shapiro.test(residuals_PBR525)

      Shapiro-Wilk normality test

data:  residuals_PBR525
W = 0.98162, p-value = 0.9732

> hist(residuals_PBR525,
+      main = "Histogram of Residuals for PBR525 (Group Y)",
+      xlab = "Residuals",
+      ylab = "Frequency",
+      col = "lightblue",
+      border = "black",
+      prob = TRUE)

> curve(dnorm(x, mean = mean(residuals_PBR525, na.rm = TRUE),
+             sd = sd(residuals_PBR525, na.rm = TRUE)),
+       col = "blue", lwd = 2, add = TRUE)
```

PBR59

```
> lm_PBR59 <- lm(PBR59 ~ IV_fluids_rate + IV_fluids_duration +
IV_fluids_total,
+              data = subset_data)
> summary(lm_PBR59)

Call:
lm(formula = PBR59 ~ IV_fluids_rate + IV_fluids_duration +
IV_fluids_total,
    data = subset_data)

Residuals:
     Min       1Q   Median       3Q      Max
-0.38710 -0.04725  0.04770  0.07507  0.19355

Coefficients:
                     Estimate Std. Error t value Pr(>|t|)
(Intercept)         1.342e+00  2.814e-01   4.770   0.0031 **
IV_fluids_rate     -2.018e-03  1.761e-02  -0.115   0.9125
IV_fluids_duration  2.826e-03  1.586e-02   0.178   0.8644
IV_fluids_total     6.359e-06  1.050e-03   0.006   0.9954
---
Signif. codes:  0 ‘***’ 0.001 ‘**’ 0.01 ‘*’ 0.05 ‘.’ 0.1 ‘ ’ 1

Residual standard error: 0.2013 on 6 degrees of freedom
Multiple R-squared:  0.0558,  Adjusted R-squared:  -0.4163
F-statistic: 0.1182 on 3 and 6 DF,  p-value: 0.9461

> residuals_PBR59 <- residuals(lm_PBR59)
> shapiro.test(residuals_PBR59)

      Shapiro-Wilk normality test

data:  residuals_PBR59
W = 0.87729, p-value = 0.1214

> hist(residuals_PBR59,
```

```
+       main = "Histogram of Residuals for PBR59 (Group Y)",
+       xlab = "Residuals",
+       ylab = "Frequency",
+       col = "lightblue",
+       border = "black",
+       prob = TRUE)
> curve(dnorm(x, mean = mean(residuals_PBR59, na.rm = TRUE),
+               sd = sd(residuals_PBR59, na.rm = TRUE)),
+        col = "blue", lwd = 2, add = TRUE)
>
```

PBR1019

```
> lm_PBR1019 <- lm(PBR1019 ~ IV_fluids_rate + IV_fluids_duration +
IV_fluids_total,
+                data = subset_data)
>
> summary(lm_PBR1019)

Call:
lm(formula = PBR1019 ~ IV_fluids_rate + IV_fluids_duration +
    IV_fluids_total, data = subset_data)

Residuals:
     Min       1Q   Median       3Q      Max
-0.32821 -0.11378 -0.01295  0.12643  0.37616

Coefficients:
                     Estimate Std. Error t value Pr(>|t|)
(Intercept)         3.2412881  0.3441530   9.418 8.14e-05 ***
IV_fluids_rate     -0.0191422  0.0215372  -0.889    0.408
IV_fluids_duration -0.0010220  0.0193947  -0.053    0.960
IV_fluids_total     0.0001254  0.0012843   0.098    0.925
---
Signif. codes:  0 ‘***’ 0.001 ‘**’ 0.01 ‘*’ 0.05 ‘.’ 0.1 ‘ ’ 1

Residual standard error: 0.2462 on 6 degrees of freedom
Multiple R-squared:  0.2959,  Adjusted R-squared:  -0.05618
F-statistic: 0.8404 on 3 and 6 DF,  p-value: 0.5195

> residuals_PBR1019 <- residuals(lm_PBR1019)

> shapiro.test(residuals_PBR1019)

      Shapiro-Wilk normality test

data:  residuals_PBR1019
W = 0.97691, p-value = 0.9465

> hist(residuals_PBR1019,
+       main = "Histogram of Residuals for PBR1019 (Group Y)",
+       xlab = "Residuals",
+       ylab = "Frequency",
+       col = "lightblue",
+       border = "black",
+       prob = TRUE)

> curve(dnorm(x, mean = mean(residuals_PBR1019, na.rm = TRUE),
+               sd = sd(residuals_PBR1019, na.rm = TRUE)),
+        col = "blue", lwd = 2, add = TRUE)
```

PBR2025

```
> lm_PBR2025 <- lm(PBR2025 ~ IV_fluids_rate + IV_fluids_duration +
IV_fluids_total,
+                data = subset_data)
> summary(lm_PBR2025)

Call:
```

```
lm(formula = PBR2025 ~ IV_fluids_rate + IV_fluids_duration +
    IV_fluids_total, data = subset_data)

Residuals:
     Min       1Q   Median       3Q      Max
-0.48245 -0.13922 -0.04434  0.09291  0.56708

Coefficients:
                    Estimate Std. Error t value Pr(>|t|)
(Intercept)         3.428024   0.555200   6.174 0.000829 ***
IV_fluids_rate     -0.047650   0.034745  -1.371 0.219314
IV_fluids_duration -0.024080   0.031288  -0.770 0.470729
IV_fluids_total     0.002703   0.002072   1.304 0.239864
---
Signif. codes:  0 '***' 0.001 '**' 0.01 '*' 0.05 '.' 0.1 ' ' 1

Residual standard error: 0.3971 on 6 degrees of freedom
Multiple R-squared:  0.3865,  Adjusted R-squared:  0.07976
F-statistic:  1.26 on 3 and 6 DF,  p-value: 0.369

> residuals_PBR2025 <- residuals(lm_PBR2025)
> shapiro.test(residuals_PBR2025)

      Shapiro-Wilk normality test

data:  residuals_PBR2025
W = 0.94971, p-value = 0.6651

> hist(residuals_PBR2025,
+      main = "Histogram of Residuals for PBR2025 (Group Y)",
+      xlab = "Residuals",
+      ylab = "Frequency",
+      col = "lightblue",
+      border = "black",
+      prob = TRUE)
> curve(dnorm(x, mean = mean(residuals_PBR2025, na.rm = TRUE),
+              sd = sd(residuals_PBR2025, na.rm = TRUE)),
+       col = "blue", lwd = 2, add = TRUE)
```

Glycocheck-TM vs ATT for trauma subset

```
> subset_data_T <- subset(Glycoheck_clinical_study_dataset_for_stats, Grou
p == "T")
>
```

VVD

```
> lm_VVD <- lm(VVD ~ Total_ATT, data = subset_data_T)
>
> summary(lm_VVD)

Call:
lm(formula = VVD ~ Total_ATT, data = subset_data_T)

Residuals:
    Min      1Q  Median      3Q     Max
-126.94  -72.61   18.06   62.73  106.75

Coefficients:
            Estimate Std. Error t value Pr(>|t|)
(Intercept)   406.38      79.92   5.085 0.000658 ***
Total_ATT     -12.36      18.42  -0.671 0.519122
---
Signif. codes:  0 '***' 0.001 '**' 0.01 '*' 0.05 '.' 0.1 ' ' 1

Residual standard error: 88.18 on 9 degrees of freedom
```

```
Multiple R-squared:  0.04763,	Adjusted R-squared:  -0.05819 
F-statistic: 0.4501 on 1 and 9 DF,  p-value: 0.5191

> residuals_VVD <- residuals(lm_VVD)
> shapiro.test(residuals_VVD)

	Shapiro-Wilk normality test

data:  residuals_VVD
W = 0.90014, p-value = 0.1856

> hist(residuals_VVD,
+      main = "Histogram of Residuals for VVD (Group T)",
+      xlab = "Residuals",
+      ylab = "Frequency",
+      col = "lightblue",
+      border = "black",
+      prob = TRUE)
> 
> curve(dnorm(x, mean = mean(residuals_VVD, na.rm = TRUE), 
+             sd = sd(residuals_VVD, na.rm = TRUE)), 
+       col = "blue", lwd = 2, add = TRUE)
```

RBC filling

```
> lm_RBC_filling <- lm(RBC_filling ~ Total_ATT, data = subset_data_T)
> summary(lm_RBC_filling)

Call:
lm(formula = RBC_filling ~ Total_ATT, data = subset_data_T)

Residuals:
      Min        1Q    Median        3Q       Max 
-0.090833 -0.063208 -0.000917  0.042417  0.143083 

Coefficients:
            Estimate Std. Error t value Pr(>|t|)    
(Intercept)  0.55458    0.07423   7.471 3.81e-05 ***
Total_ATT    0.00725    0.01711   0.424    0.682    
---
Signif. codes:  0 ‘***’ 0.001 ‘**’ 0.01 ‘*’ 0.05 ‘.’ 0.1 ‘ ’ 1

Residual standard error: 0.0819 on 9 degrees of freedom
Multiple R-squared:  0.01956,	Adjusted R-squared:  -0.08938 
F-statistic: 0.1795 on 1 and 9 DF,  p-value: 0.6817

> residuals_RBC_filling <- residuals(lm_RBC_filling)

> shapiro.test(residuals_RBC_filling)

	Shapiro-Wilk normality test

data:  residuals_RBC_filling
W = 0.9151, p-value = 0.2799

> hist(residuals_RBC_filling,
+      main = "Histogram of Residuals for RBC_filling (Group T)",
+      xlab = "Residuals",
+      ylab = "Frequency",
+      col = "lightblue",
+      border = "black",
+      prob = TRUE)
> 
> curve(dnorm(x, mean = mean(residuals_RBC_filling, na.rm = TRUE), 
+             sd = sd(residuals_RBC_filling, na.rm = TRUE)), 
+       col = "blue", lwd = 2, add = TRUE)
```

Median P50

```
> lm_Median_P50 <- lm(Median_P50 ~ Total_ATT, data = subset_data_T)
```

```
> summary(lm_Median_P50)

Call:
lm(formula = Median_P50 ~ Total_ATT, data = subset_data_T)

Residuals:
     Min       1Q   Median       3Q      Max 
-1.69676 -1.06894 -0.02676  1.03773  1.39991 

Coefficients:
            Estimate Std. Error t value Pr(>|t|)    
(Intercept)   9.7275     1.1144   8.729  1.1e-05 ***
Total_ATT    -0.3410     0.2569  -1.327    0.217    
---
Signif. codes:  0 '***' 0.001 '**' 0.01 '*' 0.05 '.' 0.1 ' ' 1

Residual standard error: 1.23 on 9 degrees of freedom
Multiple R-squared:  0.1637,	Adjusted R-squared:  0.07082 
F-statistic: 1.762 on 1 and 9 DF,  p-value: 0.217

> residuals_Median_P50 <- residuals(lm_Median_P50)
> shapiro.test(residuals_Median_P50)

	Shapiro-Wilk normality test

data:  residuals_Median_P50
W = 0.89735, p-value = 0.1715

> hist(residuals_Median_P50,
+      main = "Histogram of Residuals for Median_P50 (Group T)",
+      xlab = "Residuals",
+      ylab = "Frequency",
+      col = "lightblue",
+      border = "black",
+      prob = TRUE)
> 
> curve(dnorm(x, mean = mean(residuals_Median_P50, na.rm = TRUE),
+              sd = sd(residuals_Median_P50, na.rm = TRUE)),
+       col = "blue", lwd = 2, add = TRUE)
```

PBR525

```
> lm_PBR525 <- lm(PBR525 ~ Total_ATT, data = subset_data_T)
> summary(lm_PBR525)

Call:
lm(formula = PBR525 ~ Total_ATT, data = subset_data_T)

Residuals:
     Min       1Q   Median       3Q      Max 
-0.32107 -0.17119 -0.05417  0.19060  0.30024 

Coefficients:
            Estimate Std. Error t value Pr(>|t|)    
(Intercept)  2.70488    0.20861  12.966 3.97e-07 ***
Total_ATT   -0.02512    0.04809  -0.522    0.614    
---
Signif. codes:  0 '***' 0.001 '**' 0.01 '*' 0.05 '.' 0.1 ' ' 1

Residual standard error: 0.2302 on 9 degrees of freedom
Multiple R-squared:  0.02942,	Adjusted R-squared:  -0.07842 
F-statistic: 0.2728 on 1 and 9 DF,  p-value: 0.614

> residuals_PBR525 <- residuals(lm_PBR525)
> shapiro.test(residuals_PBR525)

	Shapiro-Wilk normality test

data:  residuals_PBR525
```

```
W = 0.92963, p-value = 0.4071

> hist(residuals_PBR525,
+      main = "Histogram of Residuals for PBR525 (Group T)",
+      xlab = "Residuals",
+      ylab = "Frequency",
+      col = "lightblue",
+      border = "black",
+      prob = TRUE)
>
> curve(dnorm(x, mean = mean(residuals_PBR525, na.rm = TRUE),
+             sd = sd(residuals_PBR525, na.rm = TRUE)),
+       col = "blue", lwd = 2, add = TRUE)
```

PBR59

```
> lm_PBR59 <- lm(PBR59 ~ Total_ATT, data = subset_data_T)
> summary(lm_PBR59)

Call:
lm(formula = PBR59 ~ Total_ATT, data = subset_data_T)

Residuals:
     Min       1Q   Median       3Q      Max 
-0.36413 -0.06246  0.05841  0.10984  0.22587 

Coefficients:
             Estimate Std. Error t value Pr(>|t|)    
(Intercept)  1.365714   0.166987   8.179 1.85e-05 ***
Total_ATT   -0.005397   0.038494  -0.140    0.892    
---
Signif. codes:  0 ‘***’ 0.001 ‘**’ 0.01 ‘*’ 0.05 ‘.’ 0.1 ‘ ’ 1

Residual standard error: 0.1842 on 9 degrees of freedom
Multiple R-squared:  0.002179, Adjusted R-squared:  -0.1087 
F-statistic: 0.01966 on 1 and 9 DF,  p-value: 0.8916

> residuals_PBR59 <- residuals(lm_PBR59)
> shapiro.test(residuals_PBR59)

        Shapiro-Wilk normality test

data:  residuals_PBR59
W = 0.92885, p-value = 0.3993

> hist(residuals_PBR59,
+      main = "Histogram of Residuals for PBR59 (Group T)",
+      xlab = "Residuals",
+      ylab = "Frequency",
+      col = "lightblue",
+      border = "black",
+      prob = TRUE)
>
> curve(dnorm(x, mean = mean(residuals_PBR59, na.rm = TRUE),
+             sd = sd(residuals_PBR59, na.rm = TRUE)),
+       col = "blue", lwd = 2, add = TRUE)
```

PBR1019

```
> lm_PBR1019 <- lm(PBR1019 ~ Total_ATT, data = subset_data_T)
> summary(lm_PBR1019)

Call:
lm(formula = PBR1019 ~ Total_ATT, data = subset_data_T)

Residuals:
     Min       1Q   Median       3Q      Max 
-0.25397 -0.14397 -0.03333  0.15302  0.27508 

Coefficients:
            Estimate Std. Error t value Pr(>|t|)
```

```
(Intercept)  3.09190    0.17587  17.581 2.82e-08 ***
Total_ATT   -0.01698    0.04054  -0.419    0.685
---
Signif. codes:  0 ‘***’ 0.001 ‘**’ 0.01 ‘*’ 0.05 ‘.’ 0.1 ‘ ’ 1

Residual standard error: 0.194 on 9 degrees of freedom
Multiple R-squared:  0.01913,	Adjusted R-squared:  -0.08986 
F-statistic: 0.1755 on 1 and 9 DF,  p-value: 0.6851

> residuals_PBR1019 <- residuals(lm_PBR1019)
> shapiro.test(residuals_PBR1019)

	Shapiro-Wilk normality test

data:  residuals_PBR1019
W = 0.92992, p-value = 0.41

> hist(residuals_PBR1019,
+      main = "Histogram of Residuals for PBR1019 (Group T)",
+      xlab = "Residuals",
+      ylab = "Frequency",
+      col = "lightblue",
+      border = "black",
+      prob = TRUE)
> 
> curve(dnorm(x, mean = mean(residuals_PBR1019, na.rm = TRUE),
+             sd = sd(residuals_PBR1019, na.rm = TRUE)),
+       col = "blue", lwd = 2, add = TRUE)
```

PBR2025

```
> lm_PBR2025 <- lm(PBR2025 ~ Total_ATT, data = subset_data_T)
> summary(lm_PBR2025)

Call:
lm(formula = PBR2025 ~ Total_ATT, data = subset_data_T)

Residuals:
     Min       1Q   Median       3Q      Max 
-0.49316 -0.29983 -0.05172  0.29801  0.52306 

Coefficients:
            Estimate Std. Error t value Pr(>|t|)    
(Intercept)  3.26893    0.35545   9.197 7.15e-06 ***
Total_ATT   -0.05144    0.08194  -0.628    0.546    
---
Signif. codes:  0 ‘***’ 0.001 ‘**’ 0.01 ‘*’ 0.05 ‘.’ 0.1 ‘ ’ 1

Residual standard error: 0.3922 on 9 degrees of freedom
Multiple R-squared:  0.04196,	Adjusted R-squared:  -0.06449 
F-statistic: 0.3941 on 1 and 9 DF,  p-value: 0.5457

> residuals_PBR2025 <- residuals(lm_PBR2025)
> shapiro.test(residuals_PBR2025)

	Shapiro-Wilk normality test

data:  residuals_PBR2025
W = 0.92721, p-value = 0.3833

> hist(residuals_PBR2025,
+      main = "Histogram of Residuals for PBR2025 (Group T)",
+      xlab = "Residuals",
+      ylab = "Frequency",
+      col = "lightblue",
+      border = "black",
+      prob = TRUE)
> 
> curve(dnorm(x, mean = mean(residuals_PBR2025, na.rm = TRUE),
+             sd = sd(residuals_PBR2025, na.rm = TRUE)),
```

```
+       col = "blue", lwd = 2, add = TRUE)
```

Glycocheck-TM vs PCV - Linear models

```
> Glycoheck_clinical_study_dataset_for_stats$PCV <- as.numeric(as.characte
r(Glycoheck_clinical_study_dataset_for_stats$PCV))
```

VVD

```
> lm_VVD <- lm(VVD ~ PCV, data = Glycoheck_clinical_study_dataset_for_stat
s)
> summary(lm_VVD)

Call:
lm(formula = VVD ~ PCV, data = Glycoheck_clinical_study_dataset_for_stats)

Residuals:
    Min      1Q  Median      3Q     Max 
-193.33  -42.25  -16.39   35.23  243.07 

Coefficients:
            Estimate Std. Error t value Pr(>|t|)  
(Intercept)  212.806     87.294   2.438   0.0268 *
PCV            5.270      2.548   2.068   0.0552 .
---
Signif. codes:  0 ‘***’ 0.001 ‘**’ 0.01 ‘*’ 0.05 ‘.’ 0.1 ‘ ’ 1

Residual standard error: 105.7 on 16 degrees of freedom
  (1 observation deleted due to missingness)
Multiple R-squared:  0.2109,   Adjusted R-squared:  0.1616 
F-statistic: 4.277 on 1 and 16 DF,  p-value: 0.05519

> residuals_VVD <- residuals(lm_VVD)
> shapiro.test(residuals_VVD)

        Shapiro-Wilk normality test

data:  residuals_VVD
W = 0.91899, p-value = 0.1241

> hist(residuals_VVD,
+      main = "Histogram of Residuals for VVD",
+      xlab = "Residuals",
+      ylab = "Frequency",
+      col = "lightblue",
+      border = "black",
+      prob = TRUE)
> 
> curve(dnorm(x, mean = mean(residuals_VVD, na.rm = TRUE),
+             sd = sd(residuals_VVD, na.rm = TRUE)),
+       col = "blue", lwd = 2, add = TRUE)
```

Median P50

```
> lm_Median_P50 <- lm(Median_P50 ~ PCV, data = Glycoheck_clinical_study_da
taset_for_stats)
> summary(lm_Median_P50)

Call:
lm(formula = Median_P50 ~ PCV, data = Glycoheck_clinical_study_dataset_for
_stats)

Residuals:
     Min       1Q   Median       3Q      Max 
-2.05247 -0.71457 -0.02136  0.49014  2.19796 

Coefficients:
```

```
             Estimate Std. Error t value Pr(>|t|)    
(Intercept)  9.30191    0.94363   9.858 3.35e-08 ***
PCV         -0.03238    0.02754  -1.175    0.257    
---
Signif. codes:  0 ‘***’ 0.001 ‘**’ 0.01 ‘*’ 0.05 ‘.’ 0.1 ‘ ’ 1

Residual standard error: 1.143 on 16 degrees of freedom
  (1 observation deleted due to missingness)
Multiple R-squared:  0.07948,	Adjusted R-squared:  0.02195 
F-statistic: 1.382 on 1 and 16 DF,  p-value: 0.257

> residuals_Median_P50 <- residuals(lm_Median_P50)
> shapiro.test(residuals_Median_P50)

	Shapiro-Wilk normality test

data:  residuals_Median_P50
W = 0.99083, p-value = 0.9993

> hist(residuals_Median_P50,
+      main = "Histogram of Residuals for Median_P50",
+      xlab = "Residuals",
+      ylab = "Frequency",
+      col = "lightblue",
+      border = "black",
+      prob = TRUE)
> 
> curve(dnorm(x, mean = mean(residuals_Median_P50, na.rm = TRUE),
+             sd = sd(residuals_Median_P50, na.rm = TRUE)),
+       col = "blue", lwd = 2, add = TRUE)
```

RBC filling

```
> lm_RBC_filling <- lm(RBC_filling ~ PCV, data = Glycoheck_clinical_study_
dataset_for_stats)
> summary(lm_RBC_filling)

Call:
lm(formula = RBC_filling ~ PCV, data = Glycoheck_clinical_study_dataset_fo
r_stats)

Residuals:
      Min        1Q    Median        3Q       Max 
-0.108626 -0.064823  0.004637  0.048076  0.140154 

Coefficients:
            Estimate Std. Error t value Pr(>|t|)    
(Intercept) 0.506386   0.066112   7.659  9.7e-07 ***
PCV         0.002377   0.001930   1.232    0.236    
---
Signif. codes:  0 ‘***’ 0.001 ‘**’ 0.01 ‘*’ 0.05 ‘.’ 0.1 ‘ ’ 1

Residual standard error: 0.08005 on 16 degrees of freedom
  (1 observation deleted due to missingness)
Multiple R-squared:  0.08663,	Adjusted R-squared:  0.02955 
F-statistic: 1.518 on 1 and 16 DF,  p-value: 0.2358

> residuals_RBC_filling <- residuals(lm_RBC_filling)
> shapiro.test(residuals_RBC_filling)

	Shapiro-Wilk normality test

data:  residuals_RBC_filling
W = 0.9471, p-value = 0.3813

> hist(residuals_RBC_filling,
+      main = "Histogram of Residuals for RBC_filling",
+      xlab = "Residuals",
+      ylab = "Frequency",
+      col = "lightblue",
```

```
+      border = "black",
+      prob = TRUE)
>
> curve(dnorm(x, mean = mean(residuals_RBC_filling, na.rm = TRUE),
+              sd = sd(residuals_RBC_filling, na.rm = TRUE)),
+       col = "blue", lwd = 2, add = TRUE)
```

PBR525

```
> lm_PBR525 <- lm(PBR525 ~ PCV, data = Glycoheck_clinical_study_dataset_fo
r_stats)
> summary(lm_PBR525)

Call:
lm(formula = PBR525 ~ PCV, data = Glycoheck_clinical_study_dataset_for_sta
ts)

Residuals:
     Min       1Q   Median       3Q      Max
-0.29706 -0.17832 -0.04123  0.19283  0.40988

Coefficients:
             Estimate Std. Error t value Pr(>|t|)
(Intercept)  2.684429   0.190457  14.095 1.94e-10 ***
PCV         -0.002312   0.005559  -0.416    0.683
---
Signif. codes:  0 ‘***’ 0.001 ‘**’ 0.01 ‘*’ 0.05 ‘.’ 0.1 ‘ ’ 1

Residual standard error: 0.2306 on 16 degrees of freedom
  (1 observation deleted due to missingness)
Multiple R-squared:  0.01069, Adjusted R-squared:  -0.05114
F-statistic: 0.1729 on 1 and 16 DF,  p-value: 0.683

> residuals_PBR525 <- residuals(lm_PBR525)
> shapiro.test(residuals_PBR525)

        Shapiro-Wilk normality test

data:  residuals_PBR525
W = 0.93772, p-value = 0.2648

> hist(residuals_PBR525,
+      main = "Histogram of Residuals for PBR525",
+      xlab = "Residuals",
+      ylab = "Frequency",
+      col = "lightblue",
+      border = "black",
+      prob = TRUE)
>
> curve(dnorm(x, mean = mean(residuals_PBR525, na.rm = TRUE),
+              sd = sd(residuals_PBR525, na.rm = TRUE)),
+       col = "blue", lwd = 2, add = TRUE)
```

PBR 59

```
> lm_PBR59 <- lm(PBR59 ~ PCV, data = Glycoheck_clinical_study_dataset_for_
stats)
> summary(lm_PBR59)

Call:
lm(formula = PBR59 ~ PCV, data = Glycoheck_clinical_study_dataset_for_stat
s)

Residuals:
     Min       1Q   Median       3Q      Max
-0.39528 -0.06559  0.03040  0.10942  0.19489

Coefficients:
              Estimate Std. Error t value Pr(>|t|)
(Intercept)  1.380e+00  1.310e-01  10.531 1.33e-08 ***
PCV         -9.913e-05  3.824e-03  -0.026     0.98
```

```
---
Signif. codes:  0 '***' 0.001 '**' 0.01 '*' 0.05 '.' 0.1 ' ' 1

Residual standard error: 0.1586 on 16 degrees of freedom
  (1 observation deleted due to missingness)
Multiple R-squared:  4.199e-05,       Adjusted R-squared:  -0.06246
F-statistic: 0.0006719 on 1 and 16 DF,  p-value: 0.9796

> residuals_PBR59 <- residuals(lm_PBR59)
> shapiro.test(residuals_PBR59)

        Shapiro-Wilk normality test

data:  residuals_PBR59
W = 0.91774, p-value = 0.1179

> hist(residuals_PBR59,
+      main = "Histogram of Residuals for PBR59",
+      xlab = "Residuals",
+      ylab = "Frequency",
+      col = "lightblue",
+      border = "black",
+      prob = TRUE)
>
> curve(dnorm(x, mean = mean(residuals_PBR59, na.rm = TRUE),
+              sd = sd(residuals_PBR59, na.rm = TRUE)),
+       col = "blue", lwd = 2, add = TRUE)
```

PBR1019

```
> lm_PBR1019 <- lm(PBR1019 ~ PCV, data = Glycoheck_clinical_study_dataset_
for_stats)
> summary(lm_PBR1019)

Call:
lm(formula = PBR1019 ~ PCV, data = Glycoheck_clinical_study_dataset_for_st
ats)

Residuals:
     Min       1Q   Median       3Q      Max
-0.31556 -0.18877 -0.02508  0.14422  0.49109

Coefficients:
              Estimate Std. Error t value Pr(>|t|)
(Intercept)  3.0512335  0.1940603  15.723 3.76e-11 ***
PCV         -0.0009513  0.0056647  -0.168    0.869
---
Signif. codes:  0 '***' 0.001 '**' 0.01 '*' 0.05 '.' 0.1 ' ' 1

Residual standard error: 0.235 on 16 degrees of freedom
  (1 observation deleted due to missingness)
Multiple R-squared:  0.001759, Adjusted R-squared:  -0.06063
F-statistic: 0.0282 on 1 and 16 DF,  p-value: 0.8687

> residuals_PBR1019 <- residuals(lm_PBR1019)
> shapiro.test(residuals_PBR1019)

        Shapiro-Wilk normality test

data:  residuals_PBR1019
W = 0.95868, p-value = 0.5764

> hist(residuals_PBR1019,
+      main = "Histogram of Residuals for PBR1019",
+      xlab = "Residuals",
+      ylab = "Frequency",
+      col = "lightblue",
+      border = "black",
+      prob = TRUE)
>
```

```
> curve(dnorm(x, mean = mean(residuals_PBR1019, na.rm = TRUE),
+             sd = sd(residuals_PBR1019, na.rm = TRUE)),
+       col = "blue", lwd = 2, add = TRUE)
```

PBR2025

```
> lm_PBR2025 <- lm(PBR2025 ~ PCV, data = Glycoheck_clinical_study_dataset_
for_stats)
> summary(lm_PBR2025)

Call:
lm(formula = PBR2025 ~ PCV, data = Glycoheck_clinical_study_dataset_for_st
ats)

Residuals:
     Min       1Q   Median       3Q      Max 
-0.69709 -0.23743 -0.01242  0.26453  0.51974 

Coefficients:
             Estimate Std. Error t value Pr(>|t|)    
(Intercept)  3.299892   0.315311  10.466 1.45e-08 ***
PCV         -0.007487   0.009204  -0.813    0.428    
---
Signif. codes:  0 ‘***’ 0.001 ‘**’ 0.01 ‘*’ 0.05 ‘.’ 0.1 ‘ ’ 1

Residual standard error: 0.3818 on 16 degrees of freedom
  (1 observation deleted due to missingness)
Multiple R-squared:  0.03971,	Adjusted R-squared:  -0.02031 
F-statistic: 0.6617 on 1 and 16 DF,  p-value: 0.4279

> residuals_PBR2025 <- residuals(lm_PBR2025)
> shapiro.test(residuals_PBR2025)

	Shapiro-Wilk normality test

data:  residuals_PBR2025
W = 0.95595, p-value = 0.5257

> hist(residuals_PBR2025,
+      main = "Histogram of Residuals for PBR2025",
+      xlab = "Residuals",
+      ylab = "Frequency",
+      col = "lightblue",
+      border = "black",
+      prob = TRUE)
> 
> curve(dnorm(x, mean = mean(residuals_PBR2025, na.rm = TRUE),
+             sd = sd(residuals_PBR2025, na.rm = TRUE)),
+       col = "blue", lwd = 2, add = TRUE)
```

Sample size calculations for PBR 5 - 25 based on linear models of selected clinical variables - illustrative

PCV

```
> Glycoheck_clinical_study_dataset_for_stats$PCV <-
as.numeric(as.character(Glycoheck_clinical_study_dataset_for_stats$PCV))


PBR525

> lm_PBR525 <- lm(PBR525 ~ PCV, data =
Glycoheck_clinical_study_dataset_for_stats)
> summary(lm_PBR525)

Call:
lm(formula = PBR525 ~ PCV, data =
Glycoheck_clinical_study_dataset_for_stats)

Residuals:
     Min       1Q   Median       3Q      Max
-0.29706 -0.17832 -0.04123  0.19283  0.40988

Coefficients:
             Estimate Std. Error t value Pr(>|t|)
(Intercept)  2.684429   0.190457  14.095 1.94e-10 ***
PCV         -0.002312   0.005559  -0.416    0.683
---
Signif. codes:  0 '***' 0.001 '**' 0.01 '*' 0.05 '.' 0.1 ' ' 1

Residual standard error: 0.2306 on 16 degrees of freedom
  (1 observation deleted due to missingness)
Multiple R-squared:  0.01069, Adjusted R-squared:  -0.05114
F-statistic: 0.1729 on 1 and 16 DF,  p-value: 0.683

> R2 <- 0.01069
> f2 <- R2 / (1 - R2)
> u <- 1
> pwr_result <- pwr.f2.test(u = u, f2 = f2, sig.level = 0.05, power =
0.80)
> sample_size <- ceiling(pwr_result$v + u + 1)
> cat("Required total sample size:", sample_size, "\n")
Required total sample size: 729
```

LOH

```
> lm_PBR525 <- lm(PBR525 ~ LOH, data =
Glycoheck_clinical_study_dataset_for_stats)
> summary(lm_PBR525)

Call:
lm(formula = PBR525 ~ LOH, data =
Glycoheck_clinical_study_dataset_for_stats)

Residuals:
    Min      1Q  Median      3Q     Max
-0.3200 -0.1658 -0.0831  0.1874  0.3868

Coefficients:
            Estimate Std. Error t value Pr(>|t|)
(Intercept) 2.583104   0.085083  30.360 2.99e-16 ***
LOH         0.005059   0.017330   0.292    0.774
---
Signif. codes:  0 '***' 0.001 '**' 0.01 '*' 0.05 '.' 0.1 ' ' 1
```

```
Residual standard error: 0.2258 on 17 degrees of freedom
Multiple R-squared:  0.004988,      Adjusted R-squared:  -0.05354
F-statistic: 0.08522 on 1 and 17 DF,  p-value: 0.7739

> R2 <- 0.005
> f2 <- R2 / (1 - R2)
> u <- 1
> pwr_result <- pwr.f2.test(u = u, f2 = f2, sig.level = 0.05, power =
0.80)
> sample_size <- ceiling(pwr_result$v + u + 1)
> cat("Required total sample size:", sample_size, "\n")
Required total sample size: 1564
```

APPLE fast

```
> lm_PBR525 <- lm(PBR525 ~ APPLE_fast, data =
Glycoheck_clinical_study_dataset_for_stats)
> summary(lm_PBR525)

Call:
lm(formula = PBR525 ~ APPLE_fast, data =
Glycoheck_clinical_study_dataset_for_stats)

Residuals:
     Min       1Q   Median       3Q      Max
-0.31901 -0.14978 -0.08478  0.15207  0.41279

Coefficients:
            Estimate Std. Error t value Pr(>|t|)
(Intercept) 2.453009   0.215236  11.397 2.21e-09 ***
APPLE_fast  0.008784   0.012260   0.717    0.483
---
Signif. codes:  0 ‘***’ 0.001 ‘**’ 0.01 ‘*’ 0.05 ‘.’ 0.1 ‘ ’ 1

Residual standard error: 0.223 on 17 degrees of freedom
Multiple R-squared:  0.02931, Adjusted R-squared:  -0.02778
F-statistic: 0.5134 on 1 and 17 DF,  p-value: 0.4834

> R2 <- 0.03
> f2 <- R2 / (1 - R2)
> u <- 1
> pwr_result <- pwr.f2.test(u = u, f2 = f2, sig.level = 0.05, power =
0.80)
> sample_size <- ceiling(pwr_result$v + u + 1)
> cat("Required total sample size:", sample_size, "\n")
Required total sample size: 256
```

Total ATT

```
> subset_data_T <- subset(Glycoheck_clinical_study_dataset_for_stats,
Group == "T")
```

PBR 525

```
> lm_PBR525 <- lm(PBR525 ~ Total_ATT, data = subset_data_T)
> summary(lm_PBR525)

Call:
lm(formula = PBR525 ~ Total_ATT, data = subset_data_T)

Residuals:
     Min       1Q   Median       3Q      Max
-0.32107 -0.17119 -0.05417  0.19060  0.30024

Coefficients:
            Estimate Std. Error t value Pr(>|t|)
```

```
(Intercept)  2.70488    0.20861  12.966 3.97e-07 ***
Total_ATT   -0.02512    0.04809  -0.522    0.614
---
Signif. codes:  0 ‘***’ 0.001 ‘**’ 0.01 ‘*’ 0.05 ‘.’ 0.1 ‘ ’ 1

Residual standard error: 0.2302 on 9 degrees of freedom
Multiple R-squared:  0.02942, Adjusted R-squared:  -0.07842
F-statistic: 0.2728 on 1 and 9 DF,  p-value: 0.614

> R2 <- 0.03
> f2 <- R2 / (1 - R2)
> u <- 1
> pwr_result <- pwr.f2.test(u = u, f2 = f2, sig.level = 0.05, power =
0.80)
> sample_size <- ceiling(pwr_result$v + u + 1)
> cat("Required total sample size:", sample_size, "\n")
Required total sample size: 256
```

IV fluid rate

```
> lm_PBR525 <- lm(formula = PBR525 ~ IV_fluids_rate, data = subset_data)
> summary(lm_PBR525)

Call:
lm(formula = PBR525 ~ IV_fluids_rate, data = subset_data)

Residuals:
     Min       1Q   Median       3Q      Max
-0.36071 -0.10711 -0.02337  0.14225  0.32144

Coefficients:
                Estimate Std. Error t value Pr(>|t|)
(Intercept)     2.733279   0.134526  20.318  3.6e-08 ***
IV_fluids_rate -0.011898   0.009898  -1.202    0.264
---
Signif. codes:  0 ‘***’ 0.001 ‘**’ 0.01 ‘*’ 0.05 ‘.’ 0.1 ‘ ’ 1

Residual standard error: 0.2191 on 8 degrees of freedom
Multiple R-squared:  0.153,   Adjusted R-squared:  0.04711
F-statistic: 1.445 on 1 and 8 DF,  p-value: 0.2637

> R2 <- 0.153
> f2 <- R2 / (1 - R2)
> u <- 1
> pwr_result <- pwr.f2.test(u = u, f2 = f2, sig.level = 0.05, power =
0.80)
> sample_size <- ceiling(pwr_result$v + u + 1)
> cat("Required total sample size:", sample_size, "\n")
Required total sample size: 46
```
