# Supplementary material for: Endothelial glycocalyx thickness in cats with naturally occurring trauma or non-traumatic illness: an exploratory study
Source: Front Vet Sci. 2026 Feb 23;13:1751034. doi: 10.3389/fvets.2026.1751034 (PMC12967996; doi:10.3389/fvets.2026.1751034)
Supplement: SUPPLEMENTARY TABLE 1 — Timing and type of anaesthetic interventions and associated procedures. [file Table_1.pdf]

Supplementary table 1. Type of anaesthetic intervention, agents, associated procedures and sampling time in relation to procedure in 19 cats admitted with trauma or non-traumatic systemic illness

№	Intervention	Agents	Sample at:	Intervention
1	TIVA	Acepromazine, Ketamine, Midazolam, Alfaxalone	Start	Computed tomography and gastro-intestinal endoscopy
2	Sedation	Propofol	End*	Radiographs
3	Sedation	Butorphanol, Alfaxalone	End*	Chest drain placement
4	TIVA	Methadone, Midazolam, Propofol	Start	Chest drain placement
5	Sedation	Fentanyl, Propofol	End*	Radiographs
6	Sedation	Dexmedetomidine, Methadone, Midazolam, Propofol	Start*	Wound care
7	Sedation	Unrecorded	End*	Radiographs
8	Sedation	Medetomidine, Methadone, Propofol	End*	Radiographs
9	TIVA	Dexmedetomidine, Midazolam, Ketamine, Propofol	End	Fracture repair
10	TIVA	Medetomidine, Methadone, Midazolam, Propofol	Start	Radiographs and ultrasonography
11	TIVA	Midazolam, Methadone, Alfaxalone	Start	Fracture repair
12	TIVA	Medetomidine, Methadone, Midazolam, Ketamine, Propofol	Start	Fracture repair
13	Sedation	Butorphanol, Propofol	End*	Radiographs and ultrasonography
14	Sedation	Gabapentin, Butorphanol, Propofol	End	Point-of-care ultrasound
15	TIVA	Butorphanol, midazolam, alfaxalone	End*	Radiographs
16	Unknown	Unknown	Unknown	Mandibular cerclage placement
17	Sedation	Methadone, Midazolam, Propofol	End*	Urethral catheterization
18	Sedation	Methadone, Dexmedetomidine, Propofol	Start	Wound care
19	Sedation	Gabapentin, Butorphanol, Propofol	End	Point-of-care ultrasound

TIVA: Total Intravenous Anaesthesia

*Timing unrecorded, presumed based on clinical flow (sampling at the end of short procedures involving other services (e.g. imaging) intervenes less with clinical flow when performed prior to recovery)
