# Supplementary material for: Endothelial glycocalyx thickness in cats with naturally occurring trauma or non-traumatic illness: an exploratory study
Source: Front Vet Sci. 2026 Feb 23;13:1751034. doi: 10.3389/fvets.2026.1751034 (PMC12967996; doi:10.3389/fvets.2026.1751034)
Supplement: SUPPLEMENTARY TABLE 2 — Demographic variables and GlycoCheck-TM parameters per group. [file Table_2.pdf]

Supplementary Table 2. Descriptive statistics for demographic variables and GlycoCheck™ parameters in 11 cats with trauma and eight cats with non-traumatic illness presented per group

Variable	Mean	SD	Median	Min	Max	Q1	Q3
**Non-traumatic systemic illness group**							
Age (years)	6.47	4.68	7.76	0.83	13.01	1.71	9.46
Weight (kg)	4.57	1.77	4.25	2.70	8.14	3.42	5.24
LOH	5.00	3.74	5.50	1.00	10.00	1.00	7.50
$APPLE_{fast}$	17.00	5.26	16.50	8.00	24.00	14.75	19.50
VVD	426.92	136.09	418.67	195.00	666.67	378.00	488.25
%RBC filling	0.59	0.09	0.60	0.43	0.73	0.56	0.62
PBR 5-25 µm	2.60	0.23	2.56	2.33	2.98	2.43	2.76
PBR 5-9 µm	1.43	0.10	1.42	1.31	1.57	1.36	1.48
PBR10-19 µm	3.01	0.28	2.98	2.70	3.50	2.79	3.14
PBR 20-25 µm	3.00	0.40	3.09	2.30	3.41	2.75	3.33
Median P50	8.15	0.95	8.28	6.55	9.76	7.87	8.45
**Trauma group**							
Age (years)	3.67	5.38	1.00	0.33	17.02	0.69	3.13
Weight (kg)	4.24	1.28	4.67	2.50	5.91	2.90	5.31
LOH	3.09	2.34	3.00	0.00	7.00	1.50	4.50
$APPLE_{fast}$	17.09	3.70	17.00	13.00	23.00	14.00	20.00
Total_ATT	4.09	1.51	4.00	1.00	6.00	4.00	5.00
Short_ATT	2.00	1.18	2.00	0.00	4.00	1.00	3.00
VVD	355.82	85.72	381.33	225.00	455.00	284.33	421.67
%RBC filling	0.58	0.08	0.58	0.47	0.73	0.52	0.63
PBR 5-25 µm	2.60	0.22	2.51	2.28	2.98	2.46	2.80
PBR 5-9 µm	1.34	0.17	1.41	0.98	1.57	1.28	1.45
PBR10-19 µm	3.02	0.19	2.96	2.77	3.35	2.88	3.16
PBR 20-25 µm	3.06	0.38	2.96	2.57	3.61	2.76	3.38
Median P50	8.33	1.28	8.34	6.62	10.59	7.47	9.08

APPLEfast: Acute Patient Physiological and Laboratory Evaluation (fast); ATT: Animal Trauma Triage Score; LOH: length of hospitalization; Median P50: Median red blood cell column width; PBR: Perfused boundary region; %RBC filling: percentage red blood cell filling; VVD: Valid vessel density;
